# Mapping medication adherence tools in Africa: a scoping review

**DOI:** 10.1136/bmjopen-2025-112898

**Published:** 2026-09-16

**Authors:** Izaidino Jaime Muchanga, Luís Midão, Avelino Mazuze, Daniela Melo, Leovaldo Alcântara, Teodora Figueiredo, Elisio Costa, Bernardo Sousa-Pinto

**Affiliations:** 1 Department of Community Medicine, Information and Health Decision Sciences, Faculty of Medicine, University of Porto, Porto, Portugal; 2 Center for Health Technologies and Services Research, University of Porto, Porto, Portugal; 3 Xai-Xai Extension, Saint Thomas University of Mozambique, Xai-Xai, Mozambique; 4 RISE-Health, Competence Centre for Active and Healthy Ageing, Faculty of Pharmacy, University of Porto, Porto, Portugal; 5 Faculty of Health Sciences, Lúrio University, Nampula, Mozambique

**Keywords:** Chronic Disease, Clinical Decision-Making, INFECTIOUS DISEASES

## Abstract

**Objectives:**

To map medication adherence assessment tools used among African populations with chronic diseases and describe their methodological characteristics, validation status, cultural adaptation, administration methods and reported adherence rates.

**Design:**

Scoping review conducted in accordance with the Preferred Reporting Items for Systematic Reviews and Meta-Analyses extension for Scoping Reviews (PRISMA-ScR).

**Data sources:**

PubMed, Scopus and Web of Science were searched on 25 June 2026.

**Eligibility criteria:**

Studies conducted in African countries that assessed medication adherence among individuals with chronic diseases using any adherence measurement tool were eligible. Studies focusing exclusively on acute conditions, non-medication adherence behaviours, reviews, editorials, protocols and conference abstracts were excluded.

**Data extraction:**

Data were extracted using a standardised charting form and summarised descriptively. Information on study characteristics, adherence measurement tools, validation and cultural adaptation procedures, administration methods, disease-specific applicability, supplementary adherence assessment methods, and reported adherence rates was synthesised narratively.

**Results:**

98 studies conducted across 27 African countries were included. Ethiopia (n=36) and Nigeria (n=22) contributed the largest number of studies. Type 2 diabetes (n=29), hypertension (n=18) and HIV (n=13) were the most frequently investigated conditions. The 8-item Morisky Medication Adherence Scale was the most used instrument, appearing in 50 studies. Only 15 studies (15.3%) reported local validation and/or cultural adaptation of adherence instruments. Most studies relied on generic self-report measures administered by interviewers, while relatively few incorporated supplementary methods such as pharmacy refill records, pill counts, electronic monitoring, viral load assessment or biological markers. Reported medication adherence rates varied substantially across diseases, ranging from 0% to 96.8%.

**Conclusions:**

Medication adherence assessment in Africa is characterised by considerable methodological variability and limited evidence of local validation and cultural adaptation. The widespread use of generic self-report instruments and the scarcity of objective adherence measures highlight the need for greater standardisation and context-specific validation of adherence assessment tools across diverse African healthcare settings.

**STRENGTHS AND LIMITATIONS OF THIS STUDY**

- This scoping review followed the Preferred Reporting Items for Systematic Reviews and Meta-Analyses extension for Scoping Reviews (PRISMA-ScR) guidelines and systematically mapped medication adherence assessment tools used across diverse African healthcare settings and chronic disease conditions.
- The review provides a comprehensive methodological overview of adherence measurement approaches, including validation status, cultural adaptation, administration mode and the use of complementary adherence assessment methods.
- Studies from multiple African countries and a broad range of chronic diseases were included, enabling identification of methodological patterns and evidence gaps across the continent.
- As recommended for scoping reviews, no formal appraisal of methodological quality or risk of bias was conducted, limiting assessment of the robustness of individual studies.
- Restriction to studies published in English and French, together with the possibility that some studies indexed exclusively in regional databases were not captured, may have resulted in the omission of relevant evidence.

## Introduction

Medication adherence generally refers to the extent to which patients take medications as prescribed and continue treatment according to healthcare recommendations.^1 2^ Adherence to prescribed treatment regimens is essential for achieving successful therapeutic outcomes, especially in chronic disease management, where it enhances chronic disease prognosis and reduces long-term healthcare expenditures.^3 4^ Non-adherence constitutes a major public health challenge associated with poorer clinical outcomes, reduced quality of life, increased morbidity and mortality and substantial healthcare costs.^5 6^ Current evidence indicates that approximately 50% of patients receiving long-term treatment for chronic conditions do not adhere adequately to prescribed therapies, with lower adherence rates being frequently observed in low- and middle-income countries, including sub-Saharan Africa, where both communicable and non-communicable disease burdens remain high.^3 4 7^

Appropriate tools are essential for measuring medication adherence accurately. Various methods have been described, including direct methods, such as measurement of drugs or metabolites in biological samples, and indirect methods, such as pill counts, pharmacy refill records, electronic monitoring systems and self-reported questionnaires.^5 8^ The selection of an appropriate adherence assessment approach should consider the clinical context and characteristics of the target population, particularly in African settings where socioeconomic and cultural factors may significantly influence adherence behaviours and reporting accuracy.^4 9^ However, despite the importance of adherence assessment in African countries, there is limited availability of validated and culturally adapted scales. The most frequent adherence scales, such as the Morisky-Green Scale, the Morisky Medication Adherence Scale (MMAS-4 and MMAS-8), the Medication Adherence Rating Scale (MARS) and the Medication Compliance Questionnaire (MCQ),^4 8 9^ were developed and validated outside Africa, raising concerns about their direct applicability in African populations due to cultural, linguistic and socioeconomic differences.^10 11^

Although previous studies have reported medication adherence levels across different African countries and disease conditions, there remains a substantial gap in the systematic synthesis of the performance, validity and diversity of instruments used to assess medication adherence across the continent that have not been comprehensively evaluated. In fact, to date, no effort has been made to map the available adherence assessment tools while simultaneously identifying operational and methodological challenges associated with their use in African populations. Such processes are essential steps toward identifying (1) the best tools to measure adherence in the African context, and (2) gaps that need to be overcome by the development of standardised and contextually appropriate adherence assessment protocols.

Therefore, in this scoping review, we aimed to map and characterise medication adherence assessment tools used in African populations with chronic diseases, including their validation status, cultural adaptation processes, administration characteristics and reported adherence outcomes.

## Methods

This scoping review was conducted following the Preferred Reporting Items for Systematic Reviews and Meta-Analyses extension for Scoping Reviews (PRISMA-ScR) guidelines.^12^ The completed PRISMA-ScR checklist is provided as online supplemental material 1. The methodological approach was based on the framework proposed by Arksey and O’Malley,^13^ further refined by Levac *et al*,^14^ and included: identification of the research question, to explore and map the tools available for assessing medication adherence across Africa; identification of relevant studies through a systematic search strategy across multiple electronic databases and grey literature sources; selection of studies using predefined inclusion and exclusion criteria based on relevance to the topic and objectives; data extraction using a structured form to collect key characteristics, types of adherence tools, settings, populations and implementation contexts; and synthesis of results through narrative and thematic approaches, highlighting patterns, gaps and methodological diversity among the identified tools to ensure transparency and reproducibility.

10.1136/bmjopen-2025-112898.supp1 Supplementary data

A scoping review of literature was chosen because it allows for a comprehensive and detailed mapping of the various medication adherence tools used in Africa, a field that is still emerging and methodologically diverse. This approach facilitates the identification of knowledge gaps and conceptual differences, providing an essential foundation for future research and policies tailored to the complexity and heterogeneity of African contexts.

### Research question

This scoping review addressed the following research questions: (1) Which medication adherence tools have been used in African populations with chronic diseases? (2) What are the methodological characteristics of these tools, including validation status, cultural adaptation, administration mode and disease-specific applicability? (3) What medication adherence rates have been reported using these tools across African settings and chronic conditions? (4) What methodological challenges and gaps exist in medication adherence assessment in Africa?

### Eligibility criteria

Studies were included if they involved populations of any age from African countries and described the use or validation of instruments to measure medication adherence. The review considered all healthcare settings and focused exclusively on peer-reviewed articles published in English and French. Due to feasibility constraints, studies published in other languages were not included. There were no limitations on the year of publication.

Grey literature, conference abstracts, opinion pieces, editorials and other non-peer-reviewed sources were excluded.

### Information sources and search strategy

The literature search was carried out across three major databases: PubMed, Scopus and Web of Science. The final search was completed on 10 December 2024, and updated in 25 June 2026. Search terms were selected based on Medical Subject Headings (MeSH) terms and relevant keywords to ensure comprehensive coverage of the topic. The search strategy combined geographical keywords related to African countries with terms describing medication adherence (eg, “medication compliance”, “treatment nonadherence”) and terms related to measurement instruments (eg, “scale”, “tool”, “survey”, “assessment”). Boolean operators like AND, OR and NOT were used to combine and refine the search terms. No filters were applied regarding publication date or study type beyond the language and peer-review criteria. The complete search strategy is available in the online supplemental material 2.

10.1136/bmjopen-2025-112898.supp2 Supplementary data

### Study selection process

The screening process was carried out in three sequential stages: initial screening of titles, followed by abstracts and subsequently full-text articles. Two reviewers independently assessed the eligibility of studies at each stage. Any discrepancies were resolved through consultation with a third reviewer to reach consensus. The screening and selection process was facilitated using Rayyan,^15^ a web-based tool designed to streamline systematic and scoping review workflows.

### Data extraction

Data extraction was performed independently by two reviewers (IJM and LM) using a structured extraction form developed for this review. Any disagreements between the reviewers were resolved through discussion and consensus. The form was piloted to ensure clarity and consistency. Extracted data included article title, authorship and year of publication, country, study design, setting, population characteristics (including sample size, gender distribution and mean age), disease condition, adherence assessment tool, validation status, translation or cultural adaptation procedures, administration mode (self-administered or interviewer-administered), timepoint of administration when reported, disease-specific applicability of the instrument, use of additional adherence assessment methods (pill count, pharmacy refill, electronic monitoring) and reported adherence rates.

### Data analysis and synthesis

The findings were synthesised descriptively and organised into tables to facilitate interpretation and comparison. Given the scope of the review, no subgroup analyses or thematic categorisations were planned or conducted.

### Quality assessment

Consistent with PRISMA-ScR recommendations and the exploratory nature of scoping reviews, formal methodological quality appraisal or risk of bias assessment was not conducted. In fact, the objective of this study was to map evidence rather than determine intervention effectiveness.

### Patient and public involvement

None. Patients and the public were not involved in the design, conduct, reporting or dissemination plans of this scoping review.

## Results

**Figure 1:**
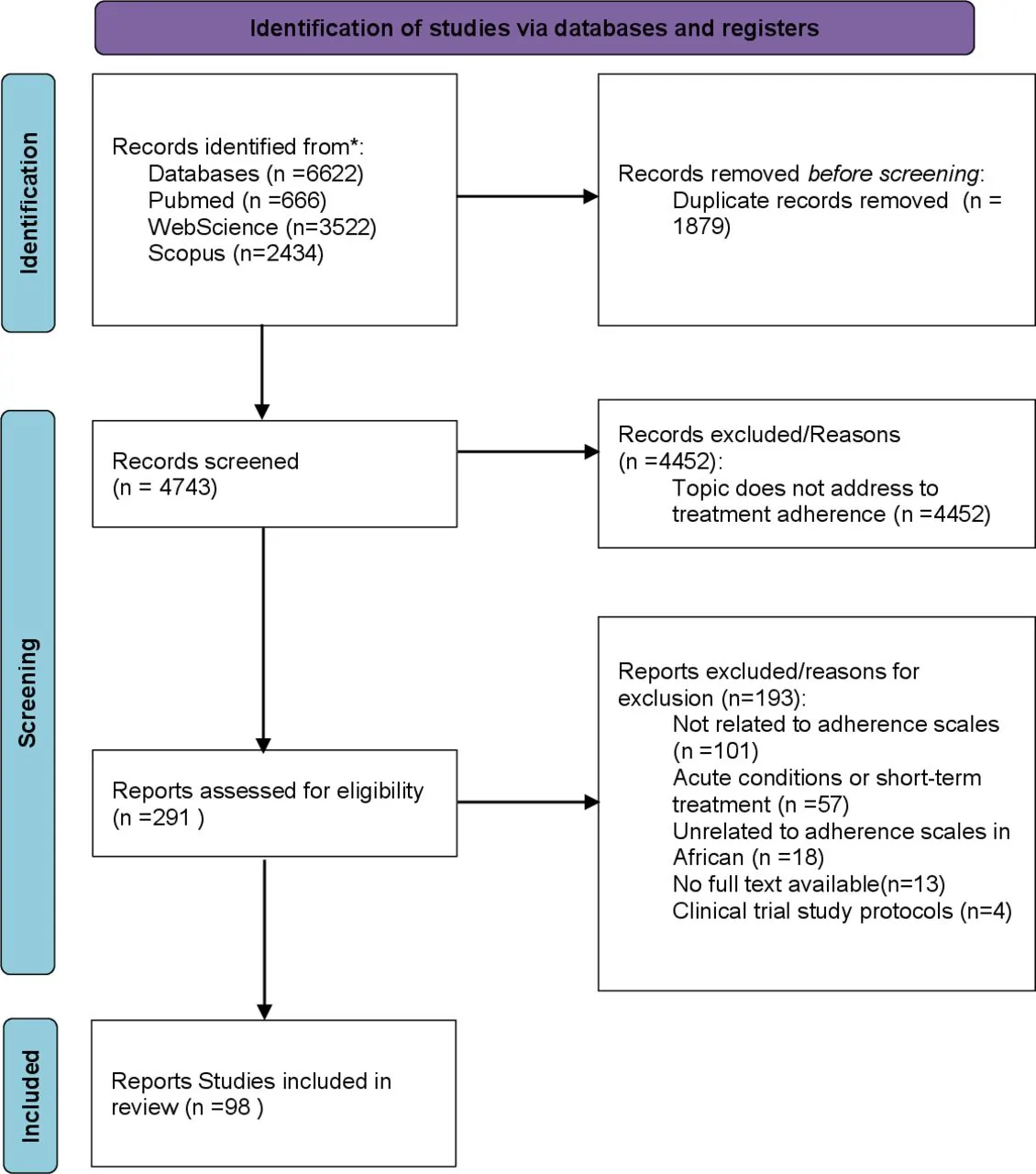
PRISMA flow chart diagram for scoping review on mapping medication adherence tools in Africa. PRISMA, Preferred Reporting Items for Systematic Reviews and Meta-Analyses.

In total, 6622 records were identified from the following databases: PubMed (n=666), Web of Science (n=3522) and Scopus (n=2434) (figure 1). Before the initial screening, 1879 duplicate records were removed, leaving 4743 records for the screening stage. No records were excluded by automation tools or for other reasons at this stage. During the screening of the 4743 records, 4452 records were excluded for not meeting the established inclusion criteria. Consequently, 291 full-text reports were selected for retrieval and detailed eligibility assessment. After this stage, 98 studies performed in 27 African countries met all eligibility criteria and were included in the review.

### Study characteristics

#### Population demographics

Demographically, the average population size across the studies was approximately 355 participants, exhibiting substantial variation (minimum of 67 and maximum of 4489). The average percentage of female participants was 59.3%. The average age of participants was approximately 46.2 years, with a range of 6.9–69.7 years. The reported medication adherence rates varied widely, with an average of 51.8%.

#### Geographical distribution

**Figure 2:**
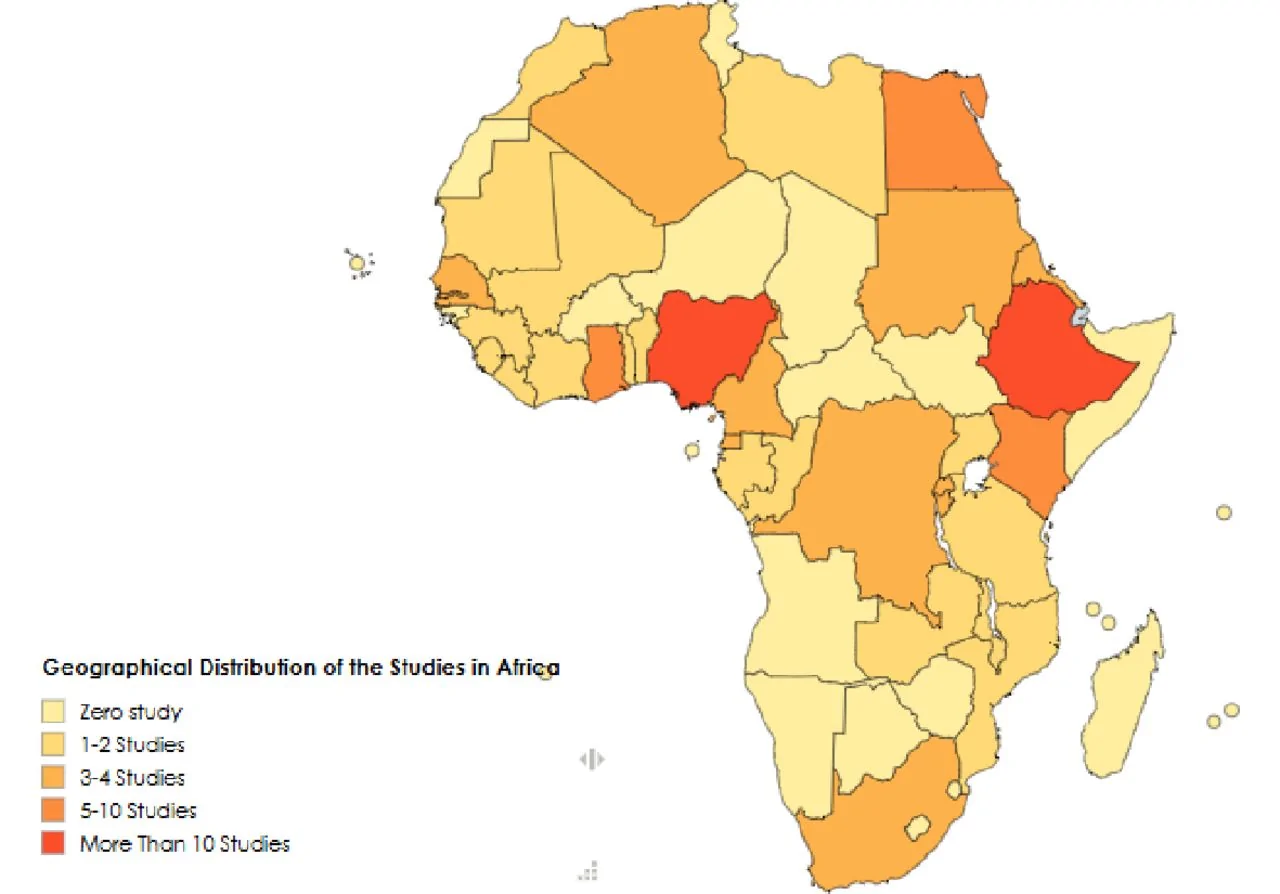
Map of geographical distribution of the studies in Africa.

The studies included in this scoping review were conducted across a range of African countries, reflecting the regional diversity of adherence research on chronic conditions. The majority of studies were conducted in Ethiopia (36 studies)^16–51^ and Nigeria (22 studies),^52–74^ highlighting these countries as key contributors to the evidence base (figure 2). Egypt accounted for five studies,^39 75–79^ while Kenya and Ghana were the setting for five studies,^80–88^ Sudan and Cameroon were the setting for four studies.^10 89–93^ South Africa was the setting for three studies,^94–96^ Uganda, Senegal, Benin, Togo and Algeria each contributed two studies.^10 84–87 89–92 95–98^ Single studies were conducted in Eritrea, Rwanda, Libya, Benin, Togo, Tanzania, Mozambique, Morocco, Gabon, Guinea, Côte d’Ivoire, Mauritania, Niger, Democratic Republic of Congo and the Republic of Congo.^11 99–106^ Additionally, one multicountry study spanned Benin, Cameroon, Congo, Democratic Republic of Congo, Gabon, Guinea, Côte d’Ivoire, Mauritania, Mozambique, Niger, Senegal and Togo.^107^

#### Disease conditions

The studies included in this scoping review covered a wide range of chronic conditions. The most frequently investigated condition was type 2 diabetes mellitus, reported in 29 studies,^11 17 28 33 34 36 37 43 47 48 60 64–66 72 76–78 83 86 87 92 97 100–102 104 108^ followed by hypertension, assessed in 18 studies.^16 22 29 35 52 54 61 62 71 73 74 81 82 89 94 98 105 107 109^ HIV was examined in 13 studies,^44 50 53 63 68 80 82 85 88 95 99 103 110^ while epilepsy was the focus of 7 studies.^18–21 25 90 96^ Other conditions addressed included type 1 diabetes mellitus (six studies),^11 28 34 37 60 108^ depressive disorders (five studies),^31 32 42 57 70^ schizophrenia (five studies),^23 24 41 59 106^ asthma (three studies),^40 49 91^ glaucoma (three studies),^45 55 84^ heart failure (three studies),^39 46 111^ rheumatoid arthritis (two studies),^10 58^ bipolar disorder (one study),^75^ breast cancer (one study),^27^ chronic kidney disease (one study),^56^ Behçet’s disease (one study),^79^ kidney transplant recipients (one study)^38^ and psychiatric outpatients (one study).^67^ Additionally, several studies examined adherence in populations with mixed chronic illnesses or complex comorbidities, such as combinations of hypertension, diabetes mellitus, arthritis, cardiac disease, renal disease and mental illness.^32 51 70 89 93 112^

#### Adherence assessment tools

Within the 98 studies included in this scoping review (online supplemental table 1), a variety of adherence measurement tools were employed. The most frequently used instrument was the 8-item MMAS (MMAS-8), applied in 50 studies.^10 16 18 20 22 26 28–30 34 37 42 43 45–48 50 52 54 60–63 68 70–72 75–78 81 83 85 89 91 94 96 97 100 101 104 105 107^ Other versions of the Morisky scale were also reported, including the 4-item MMAS (MMAS-4, used in five studies),^17 41 90 102 111^ the 9-item MMAS (MMAS-9), one study^73^ and unspecified MMAS versions (five studies).^21 25 55 56 59^ The MARS was used in 10 studies,^23 24 31 32 40 57 65 87 106 109^ either alone or combined with other tools. The 5-item MARS (MARS-5) was employed in six studies.^19 33 39 49 58 84^ The Brief Medication Questionnaire (BMQ) appeared in two studies, sometimes in combination with MARS or MMAS.^11 66^ Additional tools included the General Medication Adherence Scale (GMAS, two studies),^36 112^ Morisky Green Levine Scale (MGLS, two studies), 51 93 the Simplified Medication Adherence Questionnaire (SMAQ, five studies),^27 38 44 95 103^ the Medication Adherence Questionnaire (MAQ, one study),^86^ the Patient Medication Adherence Questionnaire (PMAQ, one study),^80^ the Compliance Questionnaire on Rheumatology (CQR, one study),^79^ the Medication Adherence Scale (one study)^107^ and several studies relied on self-reported adherence, electronic medication monitoring (Mems), pharmacy refill data or combined methods.^67 82 88 98 99 110^

10.1136/bmjopen-2025-112898.supp3 Supplementary data

### Medication adherence rates

Medication adherence rates (see online supplemental table 1) showed wide variation across different diseases analysed in studies conducted in African countries. This variability may partially reflect differences in adherence measurement tools, cut-off values, administration methods and lack of local validation. Overall, adherence to treatment was found to be strongly influenced by the type of disease, population context, assessment tools used and individual patient characteristics.

In the case of asthma, adherence rates ranged from 24.5% to 44.8%, indicating generally low adherence to controller therapy. Among individuals with mixed chronic diseases, values ranged from 48.6% to 50.6%, reflecting moderate levels of therapeutic compliance. For glaucoma, adherence ranged between 46.3% and 60.8%, while among patients with heart failure, rates varied from 36.4% to 76.3%, suggesting differences associated with clinical severity and treatment regimens.

On HIV, studies reported adherence rates between 26.7% and 96.3%, with higher adherence observed among paediatric populations and lower rates among adolescents and young adults. In cases of arterial hypertension, adherence ranged widely from 3.0% to 96.8%, demonstrating substantial heterogeneity between countries and measurement methodologies.

For other conditions, a study on Behçet’s disease reported an adherence rate of 69.2%, while bipolar I disorder showed considerably low adherence at only 19.0%. Among patients with breast cancer undergoing endocrine therapy, adherence reached 70.0% and in cardiovascular diseases, a rate of 49.0% was observed. Patients with chronic kidney disease demonstrated an adherence rate of 48.6%, whereas in kidney transplant cases, adherence reached 81.9%, reflecting a strong commitment to immunosuppressive therapy.

In mental disorders, adherence ranged from 59.0% to 62.3% for general psychiatric conditions and was 44.3% in combined severe mental disorders. Among patients with depressive disorders, rates ranged from 9.5% to 74.0%, showing high interindividual variability. In epilepsy, adherence varied widely from 4.0% to 65.9%, reflecting specific challenges in disease management.

In rheumatological conditions, rheumatoid arthritis showed the lowest adherence rates, ranging from 0.0% to 11.8%, indicating significant difficulties in treatment compliance. Schizophrenia exhibited intermediate adherence levels, with rates between 45.0% and 68.4%. In type 1 and type 2 diabetes mellitus, adherence rates were highly variable, ranging from 5.6% to 86.8%, with the lowest values reported in studies conducted in Nigeria and the highest in Ghanaian and Ethiopian contexts.

### Methodological characteristics of medication adherence assessment tools

The methodological characteristics of medication adherence assessment tools used in African studies are presented in online supplemental table 2. Across the included studies, the MMAS-8 was the most frequently used instrument, followed by MARS, SMAQ, MMAS-4, MARS-5 and other less commonly used tools such as GMAS, PMAQ, MCQ and disease-specific adherence scales.

10.1136/bmjopen-2025-112898.supp4 Supplementary data

Interviewer-administered questionnaires represented the predominant mode of administration. Self-administered approaches were less common and included paper questionnaires and electronic adherence tools. Electronic administration methods were reported primarily in HIV-related studies.

Most instruments were generic rather than disease-specific. Disease-specific tools were identified mainly in studies involving HIV, rheumatological conditions and selected chronic diseases. Additionally, only a small number of studies employed supplementary adherence assessment methods such as pill counts, pharmacy refill records, medication possession ratios, viral load monitoring, biological markers, therapeutic drug monitoring and electronic medication monitoring systems.

### Validation and cultural adaptation of adherence tools

Within the 98 studies included in this scoping review, only 15 (15.3% of the studies) reported having undertaken translation and/or cultural adaptation of the instruments to the local context. Among the included studies, eight validated the MMAS-8 across different African countries,^22 31 52 75 76 97 104 105^ while one study validated the MMAS-4,^111^ two validated the MARS-5,^39 49^ two validated the MGLS^51 93^ and two validated the MARS.^23 32^

Translation and cultural adaptation procedures varied considerably across studies. 40/98 (40.8%) studies translated tools into local languages such as Amharic, Afaan Oromo, Arabic, Yoruba, Kiswahili, Runyankore/Rukiga, Igbo, French and Lingala, while 43/98 (43.9%) studies did not report any adaptation process. Translation was frequently mentioned without detailed descriptions of forward backward translation procedures or psychometric reassessment after adaptation.

## Discussion

This scoping review mapped medication adherence assessment tools used across African populations with chronic diseases and identified substantial methodological heterogeneity in adherence measurement. A total of 98 studies conducted in 27 African countries were identified, with the MMAS-8 being the most frequently used scale, followed by MARS and other scales such as SMAQ, MCQ, PMAQ and CQR, used in specific contexts.

Adherence rates showed high variability among the 98 studies included in this scoping review, ranging from 0% to 96.8%, depending on the country, the health condition and the instrument used. This wide discrepancy may reflect both methodological challenges such as the use of non-standardised instruments and contextual factors, such as the type of disease, the age group of participants and the characteristics of the health system in place.^107^ Chronic diseases such as hypertension and diabetes generally showed lower adherence rates, which is consistent with African studies.^104 105^ On the other hand, conditions such as infection with HIV demonstrated relatively higher adherence rates in some contexts, possibly due to greater investment in adherence programmes in countries like Kenya and South Africa.^113 114^

The widespread use of the MMAS-8 adherence scale was consistent with findings from studies conducted in various African contexts, as well as international studies, which highlight its simplicity, practicality, reliability and applicability across multiple clinical conditions.^115 116^ This scale remains one of the most widely used self-report adherence measures due to its simplicity, low implementation cost and ease of administration.^117^ However, the large number of instruments identified, including MMAS-4, MARS, SMAQ, GMAS, BMQ, MAQ, PMAQ, CQR and several indirect methods, reflects the absence of a standardised approach for adherence measurement in African settings.^2^ This methodological diversity may partly explain the substantial variability observed in adherence estimates across studies.

In most studies, the absence of validation of adherence tools within local African contexts was identified.^116^ Many scales were applied directly or merely translated, without proper linguistic, sociocultural adaptation or local psychometric validation, disregarding the linguistic diversity and cultural specificities of the continent.^104 118^ For example, one study conducted in Ethiopia used the MMAS-8 translated into the local language without formal psychometric validation.^115^ This practice may introduce interpretation bias, particularly among populations with low health literacy, making it difficult to understand terms such as ‘adherence’ or ‘regular use’. Although adherence scales that are translated, culturally adapted and locally validated can be feasible and reliable, there was a notable prevalence of imported tools used without local validation. In Morocco and Sudan, for instance, the GMAS was validated; however, other scales still lack regional and local adaptations.^118 119^

Despite the widespread use of the MMAS-8 adherence scale, only eight studies conducted in Africa have reported formal psychometric and cultural validation of this adherence tool.^22 31 52 75 76 97 104 105^ Formal validation is essential to ensure that the instrument truly measures the construct of adherence within the diverse African contexts, which are characterised by linguistic diversity, health literacy barriers and cultural influence.^105 115 119 120^ In most studies, the MMAS-8 was only directly translated, without consistent psychometric validation, raising concerns about the reliability of the information produced and its applicability in monitoring multiple clinical conditions in African settings. Studies conducted in Ethiopia and Nigeria reported semantic comprehension difficulties with the MMAS-8, requiring modifications to the scale’s content to ensure clarity without compromising the essence of the original instrument.^104 121–123^ This diversity reflects the lack of standardisation in adherence assessment and highlights the need for careful consideration when comparing results across studies.

Most of the adherence scales identified were developed outside the African continent, with cultural and linguistic validations still insufficiently established in the populations where they were applied. This raises concerns regarding the cultural sensitivity and contextual appropriateness of these scales to accurately measure treatment adherence across diverse African contexts.

Beyond validation status, the findings raise important questions regarding the appropriateness of adherence instruments across different disease conditions. Most studies relied on generic self-report tools regardless of the clinical condition under investigation.^2^ While instruments such as MMAS and MARS are practical and easy to administer, they may not adequately capture disease-specific adherence challenges.^124^ Medication-taking behaviour among individuals living with HIV differs considerably from that observed in patients with diabetes, hypertension, epilepsy or severe mental illness. Disease-specific tools such as SMAQ for HIV or CQR for rheumatological diseases may provide more clinically relevant information because they address treatment characteristics unique to these conditions.^125^ The widespread use of generic instruments identified in this review suggests that important dimensions of adherence may remain insufficiently assessed in several African contexts, where cultural adaptations and disease-specific validation remain sparse.^115^

Most studies on adherence scales included in this scoping review focus on the regions of West and East Africa, with emphasis on Ethiopia, Nigeria, Egypt, Kenya and Cameroon, while countries in the North and South of the continent, such as Libya, Tunisia, Angola, Mozambique and South Africa, were under-represented or not represented at all. This may indicate a disparity in the geographical distribution of scientific production related to adherence scales across different African countries, with some countries having higher research output than others possibly due to more developed research infrastructures.^118^ This unequal distribution may create gaps in regional understanding of the barriers and facilitators to adherence, compromising the development of public policies tailored to each context.^126^

The predominance of studies conducted in hospital settings, rather than in community or rural environments, may limit the generalisability of the results, especially on a continent where most of the population lives outside urban centres. This type of study requires clinical infrastructure to ensure strict protocol control, which favours research conducted in hospital environments.^127^

The findings of this review have important implications for both research and clinical practice. Future studies should prioritise the development and validation of culturally appropriate adherence instruments capable of capturing the realities of African populations. Greater standardisation of adherence measurement would improve comparability across studies and support evidence-based decision-making. In addition, combining self-reported measures with objective methods such as pharmacy refill records, biological markers or electronic monitoring may improve measurement accuracy.

This review has several strengths. It represents the first comprehensive mapping of medication adherence assessment tools used across African populations with chronic diseases. The review followed PRISMA-ScR recommendations and included evidence from a broad range of countries, diseases, and healthcare settings.

This study has some limitations, although a systematic search strategy was conducted across various databases for this scoping review, it is possible that some relevant studies were not identified, the studies published in English and French were included, potentially excluding relevant evidence published in other languages. As recommended for scoping reviews, no formal methodological quality appraisal or risk-of-bias assessment was conducted. The heterogeneity among the studies in terms of clinical conditions and populations assessed, application contexts and languages used may limit comparisons between scales and the generalisation of results to different African settings. The geographical distribution of studies was uneven, with a strong concentration in Ethiopia and Nigeria and limited evidence from several African countries, which may affect the generalisability of findings across the continent.

In addition, although major international databases such as PubMed were included, some studies indexed exclusively in *African Journals Online* (*AJOL*) may not have been captured.

## Conclusion

In conclusion, this review has identified that important challenges remain in terms of tools to assess adherence in Africa due to the lack of standardised, validated and culturally adapted tools. The predominant use of the MMAS-8 adherence scale, without formal psychometric validation, raises concerns about the reliability and applicability of the data obtained across different linguistic, cultural and social contexts on the continent.

Future research should prioritise the contextual adaptation, psychometric validation and methodological standardisation of existing adherence assessment tools before developing entirely new instruments for African settings. Such tools should be integrated into national and regional health strategies, with guidelines to direct adaptation and psychometric validation, in order to strengthen systems for monitoring treatment adherence, improve the guidance of therapeutic interventions and promote more accurate and equitable public policies tailored to the diverse contexts across the continent.

## Supplementary Material

Reviewer comments

Author's manuscript

## Data Availability

No data are available.
