# Supplementary material for: Mapping medication adherence tools in Africa: a scoping review
Source: BMJ Open. 2026 Sep 16;16(9):e112898. doi: 10.1136/bmjopen-2025-112898 (PMC13584023; doi:10.1136/bmjopen-2025-112898)
Supplement: Supplementary file 1 [file bmjopen-16-9-s001.pdf]

Preferred Reporting Items for Systematic reviews and Meta-Analyses extension for Scoping Reviews (PRISMA-ScR) Checklist

SECTION	ITEM	PRISMA-ScR CHECKLIST ITEM	REPORTED ON PAGE #
**TITLE**			
Title	1	Identify the report as a scoping review.	**Title Section (Page 1):** The report is explicitly identified as such: *"Mapping Medication Adherence Tools in Africa: A Scoping Review"*
**ABSTRACT**			
Structured summary	2	Provide a structured summary that includes (as applicable): background, objectives, eligibility criteria, sources of evidence, charting methods, results, and conclusions that relate to the review questions and objectives.	Page 1
**INTRODUCTION**			
Rationale	3	Describe the rationale for the review in the context of what is already known. Explain why the review questions/objectives lend themselves to a scoping review approach.	*:* **Pages 2 and 3**
Objectives	4	Provide an explicit statement of the questions and objectives being addressed with reference to their key elements (e.g., population or participants, concepts, and context) or other relevant key elements used to conceptualize the review questions and/or objectives.	Page 3
**METHODS**			
Protocol and registration	5	Indicate whether a review protocol exists; state if and where it can be accessed (e.g., a Web address); and if available, provide registration information, including the registration number.	No protocol was registered for this study
Eligibility criteria	6	Specify characteristics of the sources of evidence used as eligibility criteria (e.g., years considered, language, and publication status), and provide a rationale.	Page 5 (Eligibility Criteria)
Information sources*	7	Describe all information sources in the search (e.g., databases with dates of coverage and contact with authors to identify additional sources), as well as the date the most recent search was executed.	Page 5 (Information Sources and Search Strategy)
Search	8	Present the full electronic search strategy for at least 1 database, including any limits used, such that it could be repeated.	The literature search was carried out across three major

Muchanga IJ, *et al. BMJ Open* 2026; 16:e112898. doi: 10.1136/bmjopen-2025-112898

SECTION	ITEM	PRISMA-ScR CHECKLIST ITEM	REPORTED ON PAGE #
			databases: PubMed, Scopus, and Web of Science. The final search was completed on December 10, 2024. Search terms were selected based on MeSH terms and relevant keywords to ensure comprehensive coverage of the topic. The search strategy combined geographic keywords related to African countries with terms describing medication adherence (e.g., "medication compliance", "treatment nonadherence") and terms related to measurement instruments (e.g., "scale", "tool", "survey", "assessment"). Boolean operators like AND, OR, and NOT were used to combine and refine the search terms. No filters were applied regarding publication date or study type beyond the language and peer-review criteria. The complete search strategy is available in the supplementary materials. Page 5
Selection of sources of evidence†	9	State the process for selecting sources of evidence (i.e., screening and eligibility) included in the scoping review.	Page 5 (Information Sources and Search Strategy and Study Selection Process).
Data charting process‡	10	Describe the methods of charting data from the included sources of evidence (e.g., calibrated forms or forms that have been tested by the team before their use, and whether data charting was done independently or in duplicate) and	Data Extraction (Page 6)<br><br>Data extraction was performed independently by two reviewers (I.M and L.M) using a structured extraction form developed for this review. Any disagreements

Muchanga IJ, *et al. BMJ Open* 2026; 16:e112898. doi: 10.1136/bmjopen-2025-112898

SECTION	ITEM	PRISMA-ScR CHECKLIST ITEM	REPORTED ON PAGE #
		any processes for obtaining and confirming data from investigators.	between the reviewers were resolved through discussion and consensus. The form was piloted to ensure clarity and consistency
Data items	11	List and define all variables for which data were sought and any assumptions and simplifications made.	Data Extraction (Page 6)
Critical appraisal of individual sources of evidence§	12	If done, provide a rationale for conducting a critical appraisal of included sources of evidence; describe the methods used and how this information was used in any data synthesis (if appropriate).	Page 6 (Quality Assessment).
Synthesis of results	13	Describe the methods of handling and summarizing the data that were charted.	Page 6 (Data Analysis and Synthesis)
**RESULTS**			
Selection of sources of evidence	14	Give numbers of sources of evidence screened, assessed for eligibility, and included in the review, with reasons for exclusions at each stage, ideally using a flow diagram.	Pages 7 and 8 (Results / Figure 1).
Characteristics of sources of evidence	15	For each source of evidence, present characteristics for which data were charted and provide the citations.	Pages 15-41 (Study Characteristics / Tables 1 and 2)
Critical appraisal within sources of evidence	16	If done, present data on critical appraisal of included sources of evidence (see item 12).	Not applicable
Results of individual sources of evidence	17	For each included source of evidence, present the relevant data that were charted that relate to the review questions and objectives.	**Pages 15-23 (Table1)**.
Synthesis of results	18	Summarize and/or present the charting results as they relate to the review questions and objectives.	Page 11 and 12 (Adherence Assessment Tools / Medication Adherence Rates)
**DISCUSSION**			
Summary of evidence	19	Summarize the main results (including an overview of concepts, themes, and types of evidence available), link to the review questions and objectives, and consider the relevance to key groups.	**Pages 42-45 (Discussion)**
Limitations	20	Discuss the limitations of the scoping review process.	Page 45 (Strengths and Limitations of this Study)
Conclusions	21	Provide a general interpretation of the results with respect to the review questions and objectives, as	Page 45 (Conclusion)

St. Michael's Inspired Care.

SECTION	ITEM	PRISMA-ScR CHECKLIST ITEM	REPORTED ON PAGE #
		well as potential implications and/or next steps.	
**FUNDING**			
Funding	22	Describe sources of funding for the included sources of evidence, as well as sources of funding for the scoping review. Describe the role of the funders of the scoping review.	Funding, Page 46

JBI = Joanna Briggs Institute; PRISMA-ScR = Preferred Reporting Items for Systematic reviews and Meta-Analyses extension for Scoping Reviews.

* Where *sources of evidence* (see second footnote) are compiled from, such as bibliographic databases, social media platforms, and Web sites.

† A more inclusive/heterogeneous term used to account for the different types of evidence or data sources (e.g., quantitative and/or qualitative research, expert opinion, and policy documents) that may be eligible in a scoping review as opposed to only studies. This is not to be confused with *information sources* (see first footnote).

‡ The frameworks by Arksey and O'Malley (6) and Levac and colleagues (7) and the JBI guidance (4, 5) refer to the process of data extraction in a scoping review as data charting.

§ The process of systematically examining research evidence to assess its validity, results, and relevance before using it to inform a decision. This term is used for items 12 and 19 instead of "risk of bias" (which is more applicable to systematic reviews of interventions) to include and acknowledge the various sources of evidence that may be used in a scoping review (e.g., quantitative and/or qualitative research, expert opinion, and policy document).

From: Tricco AC, Lillie E, Zarin W, O'Brien KK, Colquhoun H, Levac D, et al. PRISMA Extension for Scoping Reviews (PRISMAScR): Checklist and Explanation. Ann Intern Med. 2018;169:467–473. doi: 10.7326/M18-0850.

St. Michael's
Inspired Care.
