# Supplementary material for: Mapping medication adherence tools in Africa: a scoping review
Source: BMJ Open. 2026 Sep 16;16(9):e112898. doi: 10.1136/bmjopen-2025-112898 (PMC13584023; doi:10.1136/bmjopen-2025-112898)
Supplement: Supplementary file 2 [file bmjopen-16-9-s002.pdf]

PUBMED SEARCH STRATEGY

(Africa[Title/Abstract] OR Nigeria[Title/Abstract] OR Ethiopia[Title/Abstract] OR Egypt[Title/Abstract] OR Congo[Title/Abstract] OR Tanzania[Title/Abstract] OR South Africa[Title/Abstract] OR Kenya[Title/Abstract] OR Uganda[Title/Abstract] OR Sudan[Title/Abstract] OR Algeria[Title/Abstract] OR Morocco[Title/Abstract] OR Angola[Title/Abstract] OR Ghana[Title/Abstract] OR Mozambique[Title/Abstract] OR Madagascar[Title/Abstract] OR "Côte d'Ivoire"[Title/Abstract] OR Cameroon[Title/Abstract] OR Niger[Title/Abstract] OR Mali[Title/Abstract] OR "Burkina Faso"[Title/Abstract] OR Malawi[Title/Abstract] OR Zambia[Title/Abstract] OR Chad[Title/Abstract] OR Somalia[Title/Abstract] OR Senegal[Title/Abstract] OR Zimbabwe[Title/Abstract] OR Guinea[Title/Abstract] OR Rwanda[Title/Abstract] OR Benin[Title/Abstract] OR Burundi[Title/Abstract] OR Tunisia[Title/Abstract] OR South Sudan[Title/Abstract] OR Togo[Title/Abstract] OR "Sierra Leone" OR "Libya"[Title/Abstract] OR "Congo"OR "Central African Republic"[Title/Abstract] OR Liberia[Title/Abstract] OR Mauritania[Title/Abstract] OR Eritrea[Title/Abstract] OR Gambia[Title/Abstract] OR Botswana[Title/Abstract] OR Namibia[Title/Abstract] OR Gabon[Title/Abstract] OR Lesotho [Title/Abstract] OR "Guinea-Bissau"[Title/Abstract] OR "Equatorial Guinea"[Title/Abstract] OR Mauritius[Title/Abstract] OR Eswatini[Title/Abstract] OR Djibouti[Title/Abstract] OR Comoros[Title/Abstract] OR "Cape Verde"[Title/Abstract] OR "Sao Tome and Principe"[Title/Abstract] OR Seychelles[Title/Abstract])
AND
(medication compliance[Title/Abstract]) OR (medication adherence[Title/Abstract]) OR (medication persistance[Title/Abstract]) OR (medication noncompliance[Title/Abstract]) OR (medication nonadherence[Title/Abstract]) OR (medication nonpersistance[Title/Abstract]) OR (medication non-compliance[Title/Abstract]) OR (medication non-adherence[Title/Abstract]) OR (drug compliance[Title/Abstract]) OR (drug adherence[Title/Abstract]) OR (drug persistance[Title/Abstract]) OR (drug noncompliance[Title/Abstract]) OR (drug nonadherence[Title/Abstract]) OR (drug nonpersistance[Title/Abstract]) OR (drug non-compliance[Title/Abstract]) OR (drug non-adherence[Title/Abstract]) OR (treatment compliance[Title/Abstract]) OR (treatment adherence[Title/Abstract]) OR (treatment persistance[Title/Abstract]) OR (treatment noncompliance[Title/Abstract]) OR (treatment nonadherence[Title/Abstract]) OR (treatment nonpersistance[Title/Abstract]) OR (treatment non-compliance[Title/Abstract]) OR (treatment non-adherence[Title/Abstract])
AND
(instrument∗[Title/Abstract] OR tool∗[Title/Abstract] OR measure∗[Title/Abstract] OR metric∗[Title/Abstract] OR scale∗[Title/Abstract] OR survey∗[Title/Abstract] OR assessment∗[Title/Abstract] OR screen∗[Title/Abstract]

SCOPUS SEARCH STRATEGY

“Africa” OR “Nigeria” OR “Ethiopia” OR “Egypt” OR “Congo” OR “Tanzania” OR “South Africa” OR “Kenya” OR “Uganda” OR “Sudan” OR “Algeria” OR “Morocco” OR “Angola” OR “Ghana” OR “Mozambique” OR “Madagascar” OR “Côte d'Ivoire” OR “Cameroon” OR “Niger” OR “Mali” OR “Burkina Faso” OR “Malawi” OR “Zambia” OR “Chad” OR “Somalia” OR “Senegal” OR “Zimbabwe” OR “Guinea” OR

Muchanga IJ, *et al. BMJ Open* 2026; 16:e112898. doi: 10.1136/bmjopen-2025-112898

“Rwanda” OR “Benin” OR “Burundi” OR “Tunisia” OR “South Sudan” OR “Togo” OR “Sierra Leone” OR “Libya” OR “Congo” OR “Central African Republic” OR “Liberia” OR “Mauritania” OR “Eritrea” OR “Gambia” OR “Botswana” OR “Namibia” OR “Gabon” OR “Lesotho” OR “Guinea-Bissau” OR “Equatorial Guinea” OR “Mauritius” OR “Eswatini” OR ”Djibouti” OR “Comoros” OR “Cape Verde” OR “Sao Tome and Principe” OR “Seychelles”

“medication compliance” OR “medication adherence” OR “medication persistance” OR “medication noncompliance” OR “medication nonadherence” OR “medication nonpersistance” OR “medication non-compliance” OR “medication non-adherence” OR “drug compliance” OR “drug adherence” OR “drug persistance” OR “drug noncompliance” OR “drug nonadherence” OR “drug nonpersistance” OR “drug non-compliance” OR “drug non-adherence” OR “treatment compliance” OR “treatment adherence” OR “treatment persistance” OR “treatment noncompliance” OR “treatment nonadherence” OR “treatment nonpersistance” OR “treatment non-compliance” OR “treatment non-adherence”

“instrument” OR “tool” OR “measure” OR “metric” OR “scale” OR “survey” OR “assessment” OR “screen”OR “instruments” OR “tools” OR “measures” OR “measurement” OR “metrics” OR “scales” OR “surveys” OR “assessments” OR “screening”

WEBSCIENCE SEARCH STRATEGY

((Africa) OR (Nigeria) OR (Ethiopia) OR (Egypt) OR (Congo) OR (Tanzania) OR (South Africa) OR (Kenya) OR (Uganda) OR (Sudan) OR (Algeria) OR (Morocco) OR (Angola) OR (Ghana) OR (Mozambique) OR (Madagascar) OR (Côte d'Ivoire) OR (Cameroon) OR (Niger) OR (Mali) OR (Burkina Faso) OR (Malawi) OR (Zambia) OR (Chad) OR (Somalia) OR (Senegal) OR (Zimbabwe) OR (Guinea) OR (Rwanda) OR (Benin) OR (Burundi) OR (Tunisia) OR (South Sudan) OR (Togo) OR (Sierra Leone) OR (Libya) OR (Congo) OR (Central African Republic) OR (Liberia) OR (Mauritania) OR (Eritrea) OR (Gambia) OR (Botswana) OR (Namibia) OR (Gabon) OR (Lesotho) OR (Guinea-Bissau) OR (Equatorial Guinea) OR (Mauritius) OR (Eswatini) OR(Djibouti) OR (Comoros) OR (Cape Verde) OR (Sao Tome and Principe) OR (Seychelles))

((medication compliance) OR (medication adherence) OR (medication persistance) OR (medication noncompliance) OR (medication nonadherence) OR (medication nonpersistance) OR (medication non-compliance) OR (medication non-adherence) OR (drug compliance) OR (drug adherence) OR (drug persistance) OR (drug noncompliance) OR (drug nonadherence) OR (drug nonpersistance) OR (drug non-compliance) OR (drug non-adherence) OR (treatment compliance) OR (treatment adherence) OR (treatment persistance) OR (treatment noncompliance) OR (treatment nonadherence) OR (treatment nonpersistance) OR (treatment non-compliance) OR (treatment non-adherence))

Muchanga IJ, *et al. BMJ Open* 2026; 16:e112898. doi: 10.1136/bmjopen-2025-112898

((instrument∗) OR (tool∗) OR (measure∗) OR (metric∗) OR (scale∗) OR (survey∗) OR (assessment∗) OR (screen∗))

Muchanga IJ, *et al. BMJ Open* 2026; 16:e112898. doi: 10.1136/bmjopen-2025-112898
