# Supplementary material for: Mapping medication adherence tools in Africa: a scoping review
Source: BMJ Open. 2026 Sep 16;16(9):e112898. doi: 10.1136/bmjopen-2025-112898 (PMC13584023; doi:10.1136/bmjopen-2025-112898)
Supplement: Supplementary file 3 [file bmjopen-16-9-s003.pdf]

Supplemental Table 1: Characteristics of Studies on Medication Adherence in Patients with Chronic Diseases in Africa

Title	Author & Year	Country	Disease	Population (n)	% of Female	Population Mean Age	Settings	Study Design	Adherence Scale Tool	Adherence Rate (%)
Evaluation of factors affecting adherence to asthma controller therapy in chest clinics in a sub-Saharan African setting: a cross-sectional study [91]	Ngahane et al.2016	Cameroon	Asthma	210	63.3	41.2	Hospital	Cross-Sectional	MMAS-8	44.8
Medication regimen complexity and its impact on medication adherence and asthma control among patients with asthma in Ethiopian referral hospitals [40]	Belachew et al.2022	Ethiopia	Asthma	396	60.1	49.4	Hospital	Cross-Sectional	MARS	24.5
Reliability of the Amharic Version of the Medication Adherence Report Scale and Beliefs about Medicines Questionnaire in Patients with Asthma in Ethiopia[49]	Gebremariam et al.2025	Ethiopia	Asthma	120	55.8	44.6	Hospital	Cross-Sectional	MARS-5	20.2
Evaluation of treatment adherence in patients with Behçet's disease: its relation to disease manifestations, patients' beliefs about medications, and quality of life [79]	Zayed et al.2019	Egypt	Behçet's Disease	67	14.9	35.0	Hospital	Cross-Sectional	CQR	69.2
Psycho-demographic and clinical predictors of medication adherence in patients with bipolar I disorder in a university hospital in Egypt [75]	Okasha et al.2020	Egypt	Bipolar I Disorder	110	32.7	36.6	Hospital	Longitudinal Prospective	MMAS-8	19.0
Breast Nurse Intervention to Improve Adherence to Endocrine Therapy Among Breast Cancer Patients in South Ethiopia [27]	Getachew et al.2022	Ethiopia	Breast Cancer	162	100.0	41.8	Hospital	Cluster Randomized	SMAQ	70.0
Validation of Amharic versions of medication adherence tools among outpatients with cardiovascular diseases: a cross-sectional study[51]	Tegegn et al.2025	Ethiopia	Cardiovascular diseases	315	45.4	54.8	Hospital	Cross-Sectional	MGLS	52.1
Medication adherence among cardiac patients in Khartoum State, Sudan: a cross-sectional study [89]	Awad et al.2017	Sudan	Cardiovascular Diseases ((hypertension, dyslipidaemia, ischaemic heart disease, chronic heart failure, Arrhythmia, cardiac valve replacement, rheumatic heart disease, cerebrovascular disease)	386	57.0		Hospital	Cross-Sectional	MMAS-8	49.0

A cross-sectional study on the assessment of adherence to cardiovascular medications in Sudan heart center[93]	Mahmoud et al.2025	Sudan	Cardiovascular Diseases (Hypertension, Ischemic Heart Disease, and Heart Failure)	353	40.5	58.7	Hospital	Cross-Sectional	MGLS-4	36.5
Medication adherence and its influencing factors in community-dwelling older adults with chronic illnesses in a rural area [113]	Ahmed et al.2023	Egypt	Chronic Illnesses (hypertension, cardiac disease, renal disease, arthritis, diabetes, neurological, hepatic, osteoporosis, respiratory, gastrointestinal, thyroid)	310	52.6	69.7	Hospital	Cross-Sectional	GMAS	50.6
Evaluation of medication adherence and predictors of sub-optimal adherence among pre-dialysis patients with chronic kidney disease [56]	Okoro et al.2020	Nigeria	Chronic Kidney Disease	220	61.4	52.7	Hospital	Prospective	MMAS	48.6
Impact of pharmacist intervention on antidepressant medication adherence and disease severity in patients with major depressive disorder in fragile north-east Nigeria [57]	Yusuf et al.2021	Nigeria	Depressive Disorders	101	80.2		Hospital	Prospective Interventional	MARS	9.5
Magnitude and factors associated with treatment non-adherence among patients with depressive disorders at St. Amanuel Mental Specialized Hospital, Addis Ababa, Ethiopia, 2019: A cross sectional study [31]	Alemayehu et al. 2022	Ethiopia	Depressive Disorders	415	53.0	36.0	Hospital	Cross-Sectional	MARS	74.0
Self-Reported Adverse Drug Reactions, Medication Adherence, and Clinical Outcomes among Major Depressive Disorder Patients in Ethiopia: A Prospective Hospital Based Study [42]	Abegaz et al. 2017	Ethiopia	Depressive Disorders	217	43.8	30.9	Hospital	Cross-Sectional	MMAS-8	10.6
Effect of patients beliefs about medications on adherence to drugs in diabetic patients attending family medicine outpatient clinic in Ismailia, Egypt [76]	Salama & Saudi. 2020	Egypt	Diabetes Mellitus	82	85.4	18.6	Hospital	Cross-Sectional	MMAS-8	45.1
Medication belief as correlate of medication adherence among patients with diabetes in Edo State, Nigeria [66]	Olorunfemi and Ojewole.2019	Nigeria	Diabetes Mellitus	180	55.6	57.2	Hospital	Descriptive Correlational	BMQ and MMAS	5.6
Adherence to Treatment and Factors Affecting Adherence of Epileptic Patients at Yirgalem General Hospital, Southern Ethiopia: A Prospective Cross-Sectional Study [18]	Hasiso and Desse. 2016	Ethiopia	Epilepsy	194	43.8	33.6	Hospital	Cross-Sectional	MMAS-8	32.0

Antiepileptic Drug Adherence and Its Associated Factors among Epilepsy Patients on Follow-ups at Amanuel Mental Specialized Hospital, Ethiopia [19]	Shumet et al.2022	Ethiopia	Epilepsy	439	35.3	16.4	Hospital	Cross-Sectional	MARS-5	16.4
Antiepileptic drug adherence in children in southern Ethiopia: A cross sectional study [20]	Dima and Shibesh. 2022	Ethiopia	Epilepsy	192	33.3	6.9	Hospital	Cross-Sectional	MMAS-8	65.0
Antiepileptic Drug Nonadherence and Its Predictors among People with Epilepsy [21]	Getnet et al.2016	Ethiopia	Epilepsy	450	41.3	27.0	Hospital	Cross-Sectional	MMAS	62.2
Assessment of Patients' Adherence to Antiepileptic Medications at Dessie Referral Hospital, Chronic Follow-Up, South Wollo, Amhara Region, North East Ethiopia [25]	Kassahun et al.2018	Ethiopia	Epilepsy	88	37.5		Hospital	Cross-Sectional	MMAS	65.9
Beliefs about medication, medication adherence and seizure control amongadult epilepsy patients in Kimberley, South Africa [96]	Egenas et al.2015	South Africa	Epilepsy	197	39.1	40.0	Hospital	Descriptive Observational	MMAS-8	54.6
Factors affecting adherence to antiepileptic medications among Sudanese individuals with epilepsy: A cross-sectional survey [90]	Elsayed et al.2019	Sudan	Epilepsy	96	68.8	29.0	Hospital	Cross-Sectional	MMAS-4	4.0
Associations Between Personality Traits and Adherence to Treatment in Patients with Primary Open Angle Glaucoma in an African Population [84]	Antir et al.2021	Ghana	Glaucoma	400	52.8	56.2	Hospital	Cross-Sectional	MARS-5	60.8
Drug utilization, medication adherence and treatment satisfaction amongst glaucoma patients receiving care at secondary health facilities [55]	Bello et al.2023	Nigeria	Glaucoma	311	59.2	63.8	Hospital	Cross-Sectional	MMAS	46.3
Treatment adherence and associated factors among glaucoma patients attending Ophthalmic units of referral hospitals in North West Ethiopia, 2019 [45]	Birhanie et al.2022	Ethiopia	Glaucoma	382	30.9	57.7	Hospital	Cross-Sectional	MMAS-8	49.5
Medication Adherence and Its Determinants Among Adults With Heart Failure: A Study in Selected Hospitals of North Shewa Zone, Oromia, Ethiopia (2024)-A Multicenter Institutional-Based Cross-Sectional Study[46]	Mohammed et al.2026	Ethiopia	Heart Failure	327	50.5	43.0	Hospital	Cross-Sectional	MMAS-8	44.7
Medication Nonadherence and Associated Factors among Heart Failure Patients at University of Gondar Comprehensive	Seid et al.2023	Ethiopia	Heart Failure	245	66.5	47.8	Hospital	Cross-Sectional	MARS-5	76.3

Specialized Hospital, Northwest Ethiopia [39]										
Pharmacist intervention to improve adherence to medication among heart failure patients at North East Ethiopia hospital [112]	Tsige et al.2024	Ethiopia	Heart Failure	412	53.6	44.7	Hospital	Pre-Post Interventional	MMAS-4	36.4
Factors Influencing Adherence to Antiretroviral Therapy Among Adolescents at Busia County Referral Hospital[88]	Mayamba et al.2025	Kenya	HIV	154	52.6	14.1	Hospital	Cross-Sectional	Self-report	73.4
Cultural adaptation and psychometric validation of the Amharic adherence barriers questionnaire for HIV patients on antiretroviral therapy in Ethiopia[50]	Tarekegn et al.2026	Ethiopia	HIV	200	59.5	42.1	Hospital	Cross-Sectional	MMAS-8	61.5
Clinical Utility of Pharmacy-Based Adherence Measurement in Predicting Virologic Outcomes in an Adult HIV-Infected Cohort in Jos, North Central Nigeria [53]	Abah et al.2016	Nigeria	HIV	926	66.0	34.0	Hospital	Retrospective	Drug-Refill Visits	57.5
Factors contributing to antiretroviral drug adherence among adults living with HIV or AIDS in a Kenyan rural community [80]	Kioko et al.2017	Kenya	HIV	301	62.8	40.4	Hospital	Cross-Sectional	PMAQ	86.0
Food Insecurity Is Associated with Increased Risk of NonAdherence to Antiretroviral Therapy among HIV-Infected Adults in the Democratic Republic of Congo: A CrossSectional Study [99]	Musumari et al.2014	Democratic Republic of Congo	HIV	898	72.2	44.0	Hospital	Cross-Sectional	Self-reported Adherence	79.1
Incomplete adherence among treatment-experienced adults on antiretroviral therapy in Tanzania, Uganda and Zambia [111]	Denison et al.2015	Tanzania, Uganda And Zambia	HIV	4489	68.0	40.0	Hospital	Cross-Sectional	Self-report, provider report and pharmacy refill	85.5
Measuring adherence to antiretroviral therapy in children and adolescents in western Kenya [82]	Vreeman et al.2014	Kenya	HIV	210	100.0	8.2	Hospital	Prospective Cohort	Electronic Medication Monitoring Using Mems	96.3
Medication adherence in HIV-positive pregnant women on antiretroviral therapy attending antenatal clinics in ado metropolis, south-west nigeria: A multicentre study [63]	Aduloju et al.2020	Nigeria	HIV	170	100.0	29.8	Hospital	Cross-Sectional	MMAS-8	75.3

Mood and anxiety disorders adversely influence medication adherence to antiretroviral therapy among people living with HIV/AIDS in Nigeria [68]	Omonefe Seb-Akahomen et al.2018	Nigeria	HIV	410	75.9	40.4	Hospital	Cross-Sectional	MMAS-8	63.2
Prevalence and determinants of adherence to antiretroviral treatment among HIV patients on first-line regimen: a cross-sectional study in Dakar, Senegal [103]	Mbengue et al.2019	Senegal	HIV	150	75.3	43.0	Hospital	Cross-Sectional	SMAQ and Self-reporting tool	30.6
Socio-demographic factors associated with medication adherence among People Living with HIV in the Kumasi Metropolis, Ghana [85]	Adu et al.2022	Ghana	HIV	420	80.0	45.0	Hospital	Cross-Sectional	MMAS-8	53.1
The role of health facility and individual level characteristics on medication adherence among PLHIV on second-line antiretroviral therapy in Northeast Ethiopia: use of multi-level model [44]	Wedajo et al.2022	Ethiopia	HIV	714	56.0		Hospital	Cross-Sectional	SMAQ	69.5
Using a Self-Administered Electronic Adherence Questionnaire to Identify Poor Adherence Amongst Adolescents and Young Adults on First-Line Antiretroviral Therapy in Johannesburg, South Africa [95]	Hirasen et al. 2020	South Africa	HIV	278	78.8	31.0	Hospital	Cross-Sectional	Self-reported Adherence tool and SMAQ	26.7
Adherence to hypertension management at a rural hospital in Limpopo: A cross-sectional study[94].	Murendeni et al.2025	South Africa	Hypertension	258	71.3	58.6	Hospital	Cross-Sectional	MMAS-8	39.5
Adherence to antihypertensive medication and its correlates among individuals with hypertension in a semi-urban community of Southern Nigeria [52]	Okwuonu et al.2015	Nigeria	Hypertension	252	43.3	56.6	Hospital	Cross-Sectional	MMAS-8	31.8
Adherence to Antihypertensive Treatment and Associated Factors in Central Ethiopia [16]	Tsadik, Berhane,and Worku. 2020	Ethiopia	Hypertension	989	52.7	57.6	Hospital	Cross-Sectional	MMAS-8	36.0
Antihypertensive medication adherence and associated factors among adult hypertensive patients at Jimma University Specialized Hospital, southwest Ethiopia [22]	Asgedom et al.2018	Ethiopia	Hypertension	280	46.8	57.7	Hospital	Cross-Sectional	MMAS-8	61.8
Antihypertensive Medication Adherence and Associated Factors: A Cross-Sectional Analysis of Patients Attending a	Mebrahtu et	Eritrea	Hypertension	335	66.3	59.7	Hospital	Cross-Sectional	MARS	27.2

National Referral Hospital in Asmara, Eritrea [110]	al.2021									
Factors associated with poor adherence to medication among hypertensive patients in twelve low and middle income Sub-Saharan countries [107]	Terline et al.2019	Benin, Cameroon, Congo, Democratic Republic of Congo, Gabon, Guinea, Costa de Marfim, Mauritania, Mozambique, Niger, Senegal and Togo	Hypertension	2198	88.2	57.7	Hospital	Cross-Sectional	Medication Adherence Scale-8 (MAS-8)	30.8
Family support and medication adherence among residents with hypertension in informal settlements of Nairobi, Kenya: a mixed-method study [81]	Xiong et al.2023	Kenya	Hypertension	93	65.6	57.0	Urban Settlement	Mixed-Methods	MMAS-8	43.0
General self-efficacy and hypertension treatment adherence in Algerian private clinical settings [98]	Said Kara.2022	Algeria	Hypertension	216	52.8	25.3	Hospital	Cross-Sectional	Treatment Adherence Scale[98]	
Impact of adverse drug events and treatment satisfaction on patient adherence with antihypertensive medication – a study in ambulatory patients [29]	Berhe et al.2017	Ethiopia	Hypertension	925	61.6	57.0	Hospital	Cross-Sectional	MMAS-8	21.0
Medication adherence and its associated factors among hypertensive patients attending the Debre Tabor General Hospital, northwest Ethiopia [35]	Teshome et al.2017	Ethiopia	Hypertension	337	51.6	58.3	Hospital	Cross-Sectional	MGLS	75.1
Medication Adherence and Patient Satisfaction among Hypertensive Patients Attending Outpatient Clinic in Lagos University Teaching Hospital, Nigeria [61]	Oluwole et al.2019	Nigeria	Hypertension	500	56.8	58.9	Hospital	Cross-Sectional	MMAS-8	90.2
Medication Adherence and Perceived Family Support Among Elderly Patients with Hypertension Attending a Specialty Clinic in Lagos, Nigeria [62]	Olaniran et al.2023	Nigeria	Hypertension	293	73.0	69.3	Hospital	Cross-Sectional	MMAS-8	14.3
Relationship between Cost of Care and Medication Adherence among	Ipinnimo et	Nigeria	Hypertension	138	54.4	52.6	Hospital	Cross-Sectional	MMAS-8	34.1

Hypertensive Patients Attending a Tertiary Health Facility in Ekiti State, Nigeria [71]	al.2022									
Treatment adherence and blood pressure outcome among hypertensive out-patients in two tertiary hospitals in Sokoto, Northwestern Nigeria [73]	Adisa et al.2018	Nigeria	Hypertension	605	59.2	54.5	Hospital	Cross-Sectional	MMAS-9	8.9
Treatment satisfaction and medication adherence among hypertensive patients in a teaching hospital in Ekiti State, Nigeria [74]	Akpor and Olowolaju.2022	Nigeria	Hypertension	270	63.0		Hospital	Cross-Sectional	MMAS-8	9.3
Validity and Reliability of a Self-Reported Measure of Antihypertensive Medication Adherence in Uganda [105]	Okello et al.2016	Uganda	Hypertension	329	69.3	55.0	Hospital	Cross-Sectional	MMAS-8	3.0
Depression and medication-adherence in patients with hypertension attending a tertiary health facility in South-West Nigeria [54]	Okunrinboye et al.2019	Nigeria	Hypertension And Depression	400	62.0	59.6	Hospital	Cross-Sectional	MMAS-8	96.8
Magnitude and associated factors of poor medication adherence among diabetic and hypertensive patients visiting public health facilities in Ethiopia during the COVID-19 pandemic [30]	Shimels et al.2021	Ethiopia	Hypertension and Diabetes Mellitus	409	56.0	56.5	Hospital	Cross-Sectional	MMAS-8	28.0
Medication non-adherence and its associated factors among kidney transplant patients in a large teaching hospital in Ethiopia [38]	Derejie et al.2024	Ethiopia	Kidney Transplant	270	28.5	37.0	Hospital	Cross-Sectional	SMAQ	81.9
Medication adherence and associated factors among psychiatry patients at Asella Referral and Teaching Hospital in Oromia, Ethiopia: Institution based cross sectional study [32]	Gudeta et al.2023	Ethiopia	Mental Illness (Major depressive disorder, schizophrenia, bipolar disorders, Brief psychotic disorder and schizophreniform disorder)	395	58.0	33.0	Hospital	Cross-Sectional	MARS	62.3
Medication compliance behavior in psychiatric out-patients with psychoactive substance use comorbidity in a Nigerian tertiary hospital [67]	Okpataku et al.2015	Nigeria	Psychiatric Out-Patient	208	47.1	36.7	Hospital	Cross-Sectional	Self-Administered Medication Adherence Scale	59.0
Medication adherence among Nigerian patients with rheumatoid arthritis: a two instruments survey [58]	Ubaka et al.2021	Nigeria	Rheumatoid Arthritis	169	62.7		Hospital	Cross-Sectional	MARS-5	11.8

Status of rheumatoid arthritis practice and treatment in Sudan [10]	Ali et al. 2023	Sudan	Rheumatoid Arthritis	90	93.3	55.0	Hospital	Cross-Sectional Descriptive	MMAS-8	0.0
Impact of sociodemographic factors and depression on medication adherence in schizophrenia: Cross sectional study in Morocco [106]	Doufik et al.2024	Morocco	Schizophrenia	305	21,3	35.5	Hospital	Cross-Sectional	MARS	78.7
Antipsychotic medication non-adherence among schizophrenia patients in Central Ethiopia [23]	Tareke et al. 2018	Ethiopia	Schizophrenia	412	30.3	35.3	Hospital	Cross-Sectional	MARS	59.0
Antipsychotic medication non-adherence and factors associated among patients with schizophrenia in eastern Ethiopia [24]	Mohammed et al.2024	Ethiopia	Schizophrenia	893	18.4	34.2	Hospital	Cross-Sectional	MARS	59.0
Medication adherence among Nigerians with schizophrenia: correlation between clinico-demographic factors and quality of life [59]	Ogunnubi et al.2017	Nigeria	Schizophrenia	160	53.8	38.5	Hospital	Cross-Sectional	MMAS	45.0
Quality of life and its association with current substance use, medication nonadherence and clinical factors of people with schizophrenia in Southwest Ethiopia: a hospital-based cross-sectional study [41]	Desalegn et al. 2020	Ethiopia	Schizophrenia	351	31.1	33.0	Hospital	Cross-Sectional	MMAS-4	68.4
Prevalence and predictors of sub-optimal medication adherence among patients with severe mental illnesses in a tertiary psychiatric facility in Maiduguri, North-eastern Nigeria [70]	Ibrahim et al.2015	Nigeria	Severe Mental Illnesses (schizophrenia, bipolar disorder or severe depression)	370	43.5	35.0	Hospital	Cross-Sectional	MMAS-8	44.3
Barriers to diabetes medication adherence in North West Ethiopia [108]	Abebe et al.2014	Ethiopia	Type 1 and Type 2 Diabetes Mellitus	391	52.9	50.4	Hospital	Cross-Sectional	MMAS-8	71.2
Drug therapy problems, medication adherence and treatment satisfaction among diabetic patients on follow-up care at Tikur Anbessa Specialized Hospital, Addis Ababa, Ethiopia Gebre Teklemariam D [28]	Demoz et al.2019	Ethiopia	Type 1 and Type 2 Diabetes Mellitus	418	52.9	53.0	Hospital	Cross-Sectional	MMAS-8	45.7
Health literacy, medication belief, and demographic factors as correlates of medication adherence in person with diabetes mellitus [11]	Olorunfemi and Olaolorunpo.2023	Benin	Type 1 and Type 2 Diabetes Mellitus	180	55.6	57.2	Hospital	Descriptive Correlational	MARS and BMQ	5.6

Medication adherence and direct treatment cost among diabetes patients attending a tertiary healthcare facility in Ogbomosho, Nigeria [60]	Fadare et al.2015	Nigeria	Type 1 and Type 2 Diabetes Mellitus	129	60.5	61.4	Hospital	Cross-Sectional	MMAS-8	40.6
Medication adherence and its associated factors among diabetic patients at Zewditu Memorial Hospital, Addis Ababa, Ethiopia [34]	Muhammed et al.2017	Ethiopia	Type 1 and Type 2 Diabetes Mellitus	146	54.1	46.5	Hospital	Cross-Sectional	MMAS-8	54.8
Medication non-adherence and associated factors among diabetes patients in Felege Hiwot Referral Hospital, Bahir Dar city administration, Northwest Ethiopia [37]	Teshager and Abate. 2019	Ethiopia	Type 1 and Type 2 Diabetes Mellitus	416	41.8	45.4	Hospital	Cross-Sectional	MMAS-8	31.2
Non-adherence to antidiabetic medications and associated factors among adult type 2 diabetes mellitus patients in Northeast Ethiopia: institutional based cross-sectional study[47]	Kassaw et al.2025	Ethiopia	Type 2 Diabetes Mellitus	401	42.4	53.6	Hospital	Cross-Sectional	MMAS-8	68.8
Machine learning applications to classify and monitor medication adherence in patients with type 2 diabetes in Ethiopia[48]	Kassaw et al.2025	Ethiopia	Type 2 Diabetes Mellitus	595	45.4	55.4	Hospital	Cross-Sectional	MMAS-8	55.3
Adherence to antidiabetic medication and factors associated with non-adherence among patients with type-2 diabetes mellitus in two regional hospitals in Cameroon [92]	Aminde et al.2019	Cameroon	Type 2 Diabetes Mellitus	195	69.7	60.5	Hospital	Cross-Sectional	MCQ	45.6
Adherence to Oral Antidiabetic Medications among Type 2 Diabetic (T2DM) Patients in Chronic Ambulatory Wards of Hiwot Fana Specialized University Hospital, Harar, Eastern Ethiopia: A Cross Sectional Study [17]	Jemal et al.2017	Ethiopia	Type 2 Diabetes Mellitus	196	45.4	61.1	Hospital	Cross-Sectional	MMAS-4	70.4
Association of treatment satisfaction and physician trust with glycemic control among primary care patients with type 2 diabetes in Egypt [78]	Ahmed et al.2024	Egypt	Type 2 Diabetes Mellitus	319	57.1	59.7	Hospital	Cross-Sectional	MMAS-8 and MKQ	79.9
Factors associated with medication non-adherence in patients with type 2 diabetes mellitus: The first Algerian survey [97]	Achouri et al.2019	Algeria	Type 2 Diabetes Mellitus	403	0	60.0	Hospital	Cross-Sectional	MMAS-8	68.7
Illness perceptions of Libyans with T2DM and their influence on medication adherence: a study in a diabetes center in Tripoli [100]	Ashur et al.2015	Libya	Type 2 Diabetes Mellitus	523	58.9	54.4	Hospital	Cross-Sectional	MMAS-8	63.9
llness perception, medication adherence and glycemic control among primary	Saudi et	Egypt	Type 2 Diabetes Mellitus	265	58.5	58.8	Hospital	Cross-Sectional	MMAS-8	32.5

health-care patients with type 2 diabetes mellitus at Port Said City, Egypt [77]	al.2022									
Low medication adherence and its associated factors among patients with type 2 diabetes mellitus attending Amana Hospital in Dar es Salaam, Tanzania: a cross-sectional study [101]	Doyaa et al.2024	Tanzania	Type 2 Diabetes Mellitus	248	70.2	59.8	Hospital	Cross-Sectional	MMAS-8	26.2
Medication Adherence and Contributing Factors Among Type 2 Diabetes Patients at Adama Hospital Medical College in Eastern Ethiopia [33]	Yosef et al.2023	Ethiopia	Type 2 Diabetes Mellitus	245	44.9	48.6	Hospital	Cross-Sectional	MARS-5	29.4
Medication adherence and factors associated with poor adherence among type 2 diabetes mellitus patients on follow-up at Kenyatta National Hospital, Kenya [83]	Waari et al.2018	Kenya	Type 2 Diabetes Mellitus	290	67.6	56.6	Hospital	Cross-Sectional	MMAS-8	45.5
Medication adherence and its impact on glycemic control in type 2 diabetes mellitus patients with comorbidity: A multicenter cross-sectional study in Northwest Ethiopia [36]	Sendekie et al.2022	Ethiopia	Type 2 Diabetes Mellitus	403	45.2	45.2	Hospital	Cross-Sectional	GMAS	23.1
Medication adherence and self-care behaviours among patients with type 2 diabetes mellitus in Ghana [86]	Afaya et al.2020	Ghana	Type 2 Diabetes Mellitus	330	68.2	57.5	Hospital	Cross-Sectional	MAQ	84.5
Medication Adherence in Type 2 Diabetes Patients in Nigeria [64]	Jackson et al.2015	Nigeria	Type 2 Diabetes Mellitus	303	56.5		Hospital	Cross-Sectional	MMAS-8	19.8
Medication adherence in type 2 diabetes patients: Study of patients in alimosho general hospital, Igando, Lagos, Nigeria [65]	Olufunsho and Osuolale.2015	Nigeria	Type 2 Diabetes Mellitus	152	72.4		Hospital	Descriptive (Retrospective & Prospective)	MARS	86.8
Non-adherence to medication and associated factors among type 2 diabetes patients at Clinique Medicale Fraternite, Rwanda: a cross-sectional study [102]	Murwanashyaka et al.2022	Rwanda	Type 2 Diabetes Mellitus	200	53.0		Hospital	Cross-Sectional	MMAS-4	46.5
The Association between Diabetes-Related Distress and Medication Adherence in Adult Patients with Type 2 Diabetes Mellitus: A Cross-Sectional Study [87]	Kretchy et al.2020	Ghana	Type 2 Diabetes Mellitus	188	72.3	59.3	Hospital	Cross-Sectional	MARS	34.5
The impact of pharmacist-led medication therapy management on medication adherence in patients with type 2 diabetes	ERKU et	Ethiopia	Type 2 Diabetes Mellitus	107	43.0	60.6	Hospital	Randomized	MMAS-8	9.2

mellitus: a randomized controlled study [43]	al.2017							Controlled		
The Influence of National Health Insurance on Medication Adherence Among Outpatient Type 2 Diabetics in Southwest Nigeria [72]	Ajibola et al.2018	Nigeria	Type 2 Diabetes Mellitus	110	50.9		Hospital	Cross-Sectional	MMAS-8	10.9
Validation and psychometric properties of the 8-item Morisky Medication Adherence Scale (MMAS-8) in Type 2 diabetes patients in sub-Saharan Africa [104]	Tandon et al.2015	Togo	Type 2 Diabetes Mellitus	308	72.7	57.5	Hospital	Cross-Sectional	MMAS-8	18.8

MMAS-8 (Morisky Medication Adherence Scale – 8 items), SMAQ (Simplified Medication Adherence Questionnaire), MARS (Medication Adherence Report Scale), MCQ (Medication Compliance Questionnaire), PMAQ (Patient Medication Adherence Questionnaire), CQR (Compliance Questionnaire on Rheumatology), GMAS (General Medication Adherence Scale), MGLS (Morisky-Green-Levine Scale), MAQ (Medication Adherence Questionnaire), MAS (Medication Adherence Scale), MKQ (Medication Knowledge Questionnaire), BMQ (Brief medication Questionnaire).
