# Supplementary material for: Mapping medication adherence tools in Africa: a scoping review
Source: BMJ Open. 2026 Sep 16;16(9):e112898. doi: 10.1136/bmjopen-2025-112898 (PMC13584023; doi:10.1136/bmjopen-2025-112898)
Supplement: Supplementary file 4 [file bmjopen-16-9-s004.pdf]

Supplemental Table 2: Methodological Characteristics of Medication Adherence Assessment Tools Used in African Studies

Study	Tool	Validated locally	Translation/Cultural adaptation	Administration mode	Disease-specific tool	Additional adherence method
Evaluation of treatment adherence in patients with Behçet's disease: its relation to disease manifestations, patients' beliefs about medications, and quality of life [79]	CQR	No	Arabic translation	Interview	Yes	None
Clinical Utility of Pharmacy-Based Adherence Measurement in Predicting Virologic Outcomes in an Adult HIV-Infected Cohort in Jos, North Central Nigeria[53]	Drug-Refill Visits	Clinical utility and predictive validity of pharmacy refill metrics in a local Nigerian public care facility	Not applicable	Electronic record review	Yes	None
Measuring adherence to antiretroviral therapy in children and adolescents in western Kenya[82]	Electronic Medication Monitoring Using Mems	No	Kiswahili or English translation	Interview	Yes	Electronic Dose Monitoring and Plasma Drug Concentrations
Medication adherence and its influencing factors in community-dwelling older adults with chronic illnesses in a rural area [113]	GMAS	No	Arabic translation	Interview	No	None
Medication adherence and its impact on glycemic control in type 2 diabetes mellitus patients with comorbidity: A multicenter cross-sectional study in Northwest Ethiopia[36]	GMAS	No	Afan Oromo and Amharic	Interview	Yes	None
Medication adherence and self-care behaviours among patients with type 2 diabetes mellitus in Ghana[86]	MAQ	No	Akan	Interview	Yes	None
Medication regimen complexity and its impact on medication adherence and asthma control among patients with asthma in Ethiopian referral hospitals [40]	MARS	No	Not specified	Interview	Yes	None
Impact of pharmacist intervention on antidepressant medication adherence and disease severity in patients with major depressive disorder in fragile north-east Nigeria [57]	MARS	No	Not specified	Interview	No	None
Antihypertensive Medication Adherence and Associated Factors: A Cross-Sectional Analysis of	MARS	No	Not specified	Interview	No	None

Patients Attending a National Referral Hospital in Asmara, Eritrea [110]						
Medication adherence and associated factors among psychiatry patients at Asella Referral and Teaching Hospital in Oromia, Ethiopia: Institution based cross sectional study[32]	MARS	Yes	Amharic or Afaan Oromo	Interview	No	None
Antipsychotic medication non-adherence among schizophrenia patients in Central Ethiopia[23]	MARS	Yes	Not specified	Interview	No	None
Antipsychotic medication non-adherence and factors associated among patients with schizophrenia in eastern Ethiopia [24]	MARS	No	Afan Oromo and Amharic	Interview	No	None
Health literacy, medication belief, and demographic factors as correlates of medication adherence in person with diabetes mellitus [11]	MARS	No	Not specified	Interview	No	BMQ
Medication adherence in type 2 diabetes patients: Study of patients in alimosho general hospital, Igando, Lagos, Nigeria[65]	MARS	No	Not specified	Interview	Yes	None
The Association between Diabetes-Related Distress and Medication Adherence in Adult Patients with Type 2 Diabetes Mellitus: A Cross-Sectional Study [87]	MARS	No	Not specified	Interview	Yes	None
Impact of sociodemographic factors and depression on medication adherence in schizophrenia: Cross sectional study in Morocco[106]	MARS	No	Translated from English to Moroccan Arabic dialect	Interview	No	None
Antiepileptic Drug Adherence and Its Associated Factors among Epilepsy Patients on Follow-ups at Amanuel Mental Specialized Hospital, Ethiopia[19]	MARS-5	No	Translated into Amharic	Interview	No	None
Associations Between Personality Traits and Adherence to Treatment in Patients with Primary Open Angle Glaucoma in an African Population [84]	MARS-5	No	No	Interview	No	None
Medication Nonadherence and Associated Factors among Heart Failure Patients at University of Gondar Comprehensive Specialized Hospital, Northwest Ethiopia[39]	MARS-5	Yes	Translated into Amharic	Interview	No	None
Medication adherence among Nigerian patients with rheumatoid arthritis: a two instruments survey[58]	MARS-5	No	Not specified	Interview	No	None

Medication Adherence and Contributing Factors Among Type 2 Diabetes Patients at Adama Hospital Medical College in Eastern Ethiopia[33]	MARS-5	No	Not specified	Interview	No	None
Reliability of the Amharic Version of the Medication Adherence Report Scale and Beliefs about Medicines Questionnaire in Patients with Asthma in Ethiopia[49]	MARS-5	Yes	English to the local language (Amharic)	Interview	No	None
Adherence to antidiabetic medication and factors associated with non-adherence among patients with type-2 diabetes mellitus in two regional hospitals in Cameroon[92]	MCQ	No	Not specified	Self-Administered	No	None
Medication adherence and its associated factors among hypertensive patients attending the Debre Tabor General Hospital, northwest Ethiopia[35]	MGLS	No	Not specified	Interview	No	None
A cross-sectional study on the assessment of adherence to cardiovascular medications in Sudan heart center[93]	MGLS	Yes	English to the local language (Sudanese)	Interview	No	None
Validation of Amharic versions of medication adherence tools among outpatients with cardiovascular diseases: a cross-sectional study[51]	MGLS	Yes	English to the local language (Amharic)	Interview	No	None
Evaluation of medication adherence and predictors of sub-optimal adherence among pre-dialysis patients with chronic kidney disease [56]	MMAS	No	Not specified	Self-reported questionnaire or Interview	No	None
Medication belief as correlate of medication adherence among patients with diabetes in Edo State, Nigeria[66]	MMAS	No	Not specified	Questionnaires	No	BMQ
Antiepileptic Drug Nonadherence and Its Predictors among People with Epilepsy [21]	MMAS	No	Translated into Amharic	Interview	No	None
Assessment of Patients' Adherence to Antiepileptic Medications at Dessie Referral Hospital, Chronic Follow-Up, South Wollo, Amhara Region, North East Ethiopia[25]	MMAS	No	Translated into Amharic	Interview	No	None
Medication adherence among Nigerians with schizophrenia: correlation between clinico-demographic factors and quality of life [59]	MMAS	No	Not specified	Interview	No	None
Factors affecting adherence to antiepileptic medications among Sudanese individuals with epilepsy: A cross-sectional survey[90]	MMAS-4	No	Arabic translation	Interview	No	None

Drug utilization, medication adherence and treatment satisfaction amongst glaucoma patients receiving care at secondary health facilities[55]	MMAS-4	No	No	Interview	No	None
Pharmacist intervention to improve adherence to medication among heart failure patients at North East Ethiopia hospital[112]	MMAS-4	Yes	Translated into Amharic	Interview	No	None
Quality of life and its association with current substance use, medication nonadherence and clinical factors of people with schizophrenia in Southwest Ethiopia: a hospital-based cross-sectional study[41]	MMAS-4	No	Afaan Oromo and Amharic	Interview	No	None
Adherence to Oral Antidiabetic Medications among Type 2 Diabetic (T2DM) Patients in Chronic Ambulatory Wards of Hiwot Fana Specialized University Hospital, Harar, Eastern Ethiopia: A Cross Sectional Study[17]	MMAS-4	No	Afan Oromo and Amharic	Interview	No	None
Non-adherence to medication and associated factors among type 2 diabetes patients at Clinique Medicale Fraternite, Rwanda: a cross-sectional study[102]	MMAS-4	No	Not specified	Interview	No	None
Evaluation of factors affecting adherence to asthma controller therapy in chest clinics in a sub-Saharan African setting: a cross-sectional study [91]	MMAS-8	No	Not specified	Interview	No	None
Psycho-demographic and clinical predictors of medication adherence in patients with bipolar I disorder in a university hospital in Egypt [75]	MMAS-8	Yes	Arabic translation	Interview	No	None
Medication adherence among cardiac patients in Khartoum State, Sudan: a cross-sectional study [89]	MMAS-8	No	Translated from English into Arabic	Self-administered questionnaire	No	None
Magnitude and factors associated with treatment non-adherence among patients with depressive disorders at St. Amanuel Mental Specialized Hospital, Addis Ababa, Ethiopia, 2019: A cross sectional study [31]	MMAS-8	Yes	Translated into Amharic	Interview	No	None
Self-Reported Adverse Drug Reactions, Medication Adherence, and Clinical Outcomes among Major Depressive Disorder Patients in Ethiopia: A Prospective Hospital Based Study [42]	MMAS-8	No	Translated into Amharic	Interview	No	None
Effect of patients beliefs about medications on adherence to drugs in diabetic patients attending family medicine outpatient clinic in Ismailia, Egypt [76]	MMAS-8	Yes	Arabic translation	Questionnaires	No	None

Muchanga IJ, *et al. BMJ Open* 2026; 16:e112898. doi: 10.1136/bmjopen-2025-112898

Adherence to Treatment and Factors Affecting Adherence of Epileptic Patients at Yirgalem General Hospital, Southern Ethiopia[18]	MMAS-8	No	Translated into Amharic and Sidama	Interview	No	None
Antiepileptic drug adherence in children in southern Ethiopia: A cross-sectional study[20]	MMAS-8	No	Translated into Amharic and Sidama	Interview	No	None
Beliefs about medication, medication adherence and seizure control among adult epilepsy patients in Kimberley, South Africa[96]	MMAS-8	No	No	Interview	No	None
Treatment adherence and associated factors among glaucoma patients attending Ophthalmic units of referral hospitals in North West Ethiopia [45]	MMAS-8	No	Translated into Amharic	Interview	No	None
Medication adherence in HIV-positive pregnant women on antiretroviral therapy attending antenatal clinics in ado metropolis, south-west nigeria: A multicentre study[63]	MMAS-8	No	Not specified	Interview	No	Pill Count Method
Mood and anxiety disorders adversely influence medication adherence to antiretroviral therapy among people living with HIV/AIDS in Nigeria[68]	MMAS-8	No	Not specified	Interview	No	None
Socio-demographic factors associated with medication adherence among People Living with HIV in the Kumasi Metropolis, Ghana[85]	MMAS-8	No	Not specified	Structured questionnaire	No	None
Adherence to antihypertensive medication and its correlates among individuals with hypertension in a semi-urban community of Southern Nigeria[52]	MMAS-8	Yes	Igbo language	Interview	No	None
Adherence to Antihypertensive Treatment and Associated Factors in Central Ethiopia[16]	MMAS-8	No	Amharic or Afaan Oromo	Interview	No	None
Antihypertensive medication adherence and associated factors among adult hypertensive patients at Jimma University Specialized Hospital, southwest Ethiopia [22]	MMAS-8	Yes	Amharic or Afaan Oromo	Interview	No	None
Factors associated with poor adherence to medication among hypertensive patients in twelve low and middle income Sub-Saharan countries [107]	MMAS-8	No	Via verbal translation	Self-administered with physician assistance / Interviewer-supported	No	None

Family support and medication adherence among residents with hypertension in informal settlements of Nairobi, Kenya: a mixed-method study [81]	MMAS-8	No	Not specified	Interview	No	None
Impact of adverse drug events and treatment satisfaction on patient adherence with antihypertensive medication – a study in ambulatory patients [29]	MMAS-8	No	Amharic and Tigrigna	Interview	No	None
Medication Adherence and Patient Satisfaction among Hypertensive Patients Attending Outpatient Clinic in Lagos University Teaching Hospital, Nigeria [61]	MMAS-8	No	Not specified	Interview	No	None
Medication Adherence and Perceived Family Support Among Elderly Patients with Hypertension Attending a Specialty Clinic in Lagos, Nigeria [62]	MMAS-8	No	Not specified	Interview	No	None
Relationship between Cost of Care and Medication Adherence among Hypertensive Patients Attending a Tertiary Health Facility in Ekiti State, Nigeria[71]	MMAS-8	No	Not specified	Interview	No	None
Treatment satisfaction and medication adherence among hypertensive patients in a teaching hospital in Ekiti State, Nigeria[74]	MMAS-8	No	Not specified	Questionnaire	Yes	Pill counts or pharmacy refill records
Validity and Reliability of a Self-Reported Measure of Antihypertensive Medication Adherence in Uganda[105]	MMAS-8	Yes	Runyankore/Rukiga	Questionnaire	Yes	None
Depression and medication-adherence in patients with hypertension attending a tertiary health facility in South-West Nigeria [54]	MMAS-8	No	Yoruba language	Interview	Yes	None
Magnitude and associated factors of poor medication adherence among diabetic and hypertensive patients visiting public health facilities in Ethiopia during the COVID-19 pandemic[30]	MMAS-8	No	Amharic	Interview	Yes	None
Status of rheumatoid arthritis practice and treatment in Sudan[10]	MMAS-8	No	Not specified	Interview	No	None
Prevalence and predictors of sub-optimal medication adherence among patients with severe mental illnesses in a tertiary psychiatric facility in Maiduguri, North-eastern Nigeria [70]	MMAS-8	No	Not specified	Interview	No	None
Barriers to diabetes medication adherence in North West Ethiopia[108]	MMAS-8	No	Amharic	Interview	No	None

Drug therapy problems, medication adherence and treatment satisfaction among diabetic patients on follow-up care at Tikur Anbessa Specialized Hospital, Addis Ababa, Ethiopia Gebre Teklemariam D[28]	MMAS-8	No	Not specified	Interview	No	None
Medication adherence and direct treatment cost among diabetes patients attending a tertiary healthcare facility in Ogbomosho, Nigeria [60]	MMAS-8	No	Not specified	Interview	No	None
Medication adherence and its associated factors among diabetic patients at Zewditu Memorial Hospital, Addis Ababa, Ethiopia[34]	MMAS-8	No	Not specified	Interview	No	None
Medication non-adherence and associated factors among diabetes patients in Felege Hiwot Referral Hospital, Bahir Dar city administration, Northwest Ethiopia[37]	MMAS-8	No	Amharic	Interview	No	None
Association of treatment satisfaction and physician trust with glycemic control among primary care patients with type 2 diabetes in Egypt [78]	MMAS-8	No	Arabic	Interview	No	None
Factors associated with medication non-adherence in patients with type 2 diabetes mellitus: The first Algerian survey[97]	MMAS-8	Yes	Arabic/French	Questionnaire	No	None
Illness perceptions of Libyans with T2DM and their influence on medication adherence: a study in a diabetes center in Tripoli	MMAS-8	No	Arabic	Self-administered / Self-reported questionnaire	No	None
llness perception, medication adherence and glycemic control among primary health-care patients with type 2 diabetes mellitus at Port Said City, Egypt [100]	MMAS-8	No	Arabic	Self-administered	No	None
Low medication adherence and its associated factors among patients with type 2 diabetes mellitus attending Amana Hospital in Dar es Salaam, Tanzania: a cross-sectional study [101]	MMAS-8	No	Not specified	Self-administered	No	None
Medication adherence and factors associated with poor adherence among type 2 diabetes mellitus patients on follow-up at Kenyatta National Hospital, Kenya[83]	MMAS-8	No	kiswahili	Interview	No	None
Medication Adherence in Type 2 Diabetes Patients in Nigeria[64]	MMAS-8	No	Not specified	Interview	No	None

The impact of pharmacist-led medication therapy management on medication adherence in patients with type 2 diabetes mellitus: a randomized controlled study [43]	MMAS-8	No	Not specified	Interview	No	None
The Influence of National Health Insurance on Medication Adherence Among Outpatient Type 2 Diabetics in Southwest Nigeria [72]	MMAS-8	No	Not specified	Interview	No	None
Validation and psychometric properties of the 8-item Morisky Medication Adherence Scale (MMAS-8) in Type 2 diabetes patients in sub-Saharan Africa [104]	MMAS-8	Yes	Translated from English to French and Ewe following international guidelines (forward translation, review, reverse translation, and pilot testing)	Interview	No	MMAS-4
Medication Adherence and Its Determinants Among Adults With Heart Failure: A Study in Selected Hospitals of North Shewa Zone, Oromia, Ethiopia (2024)-A Multicenter Institutional-Based Cross-Sectional Study [46]	MMAS-8	No	English to the local language (Amharic)	Interview	No	None
Non-adherence to antidiabetic medications and associated factors among adult type 2 diabetes mellitus patients in Northeast Ethiopia: institutional based cross-sectional study[47]	MMAS-8	No	English to the local language (Amharic)	Questionnaire, administered	No	None
Machine learning applications to classify and monitor medication adherence in patients with type 2 diabetes in Ethiopia[48]	MMAS-8	No	English to the local language (Amharic)	Interview	No	None
Cultural adaptation and psychometric validation of the Amharic adherence barriers questionnaire for HIV patients on antiretroviral therapy in Ethiopia[50]	MMAS-8	No	English to the local language (Amharic)	Interview	No	None
Adherence to hypertension management at a rural hospital in Limpopo: A cross-sectional study[94]	MMAS-8	No	English to the local language (Amharic)	Interview	No	None
Treatment adherence and blood pressure outcome among hypertensive out-patients in two tertiary hospitals in Sokoto, Northwestern Nigeria [73]	MMAS-9	No	Not specified	Interview	Yes	Modified Morisky Adherence Predictor Scale (MMAPS), Brief Illness Perception Questionnaire

						(BIPQ) and Beliefs about Medicine Questionnaire (BMQ)
Factors contributing to antiretroviral drug adherence among adults living with HIV or AIDS in a Kenyan rural community[80]	PMAQ	No	Kamba translation	Interview	Yes	Self-reports / Patient recall and Pill counts
Incomplete adherence among treatment-experienced adults on antiretroviral therapy in Tanzania, Uganda and Zambia[111]	Provider report and pharmacy refill	No	Not specified	Interview	Yes	Pharmacy Medication Possession Ratio (MPR), Healthcare Provider Reports and Biological Marker
Medication compliance behavior in psychiatric out-patients with psychoactive substance use comorbidity in a Nigerian tertiary hospital[67]	Self-Administered Medication Adherence Scale	No	Not specified	Interview	No	None
Factors Influencing Adherence to Antiretroviral Therapy Among Adolescents at Busia County Referral Hospital [88]	Self-report questionnaire	Not applicable	English and administered in a bilingual format/adapted to Kiswahili	Interview	Yes	Pharmacy refill registries and clinic appointment attendance records
Food Insecurity Is Associated with Increased Risk of NonAdherence to Antiretroviral Therapy among HIV-Infected Adults in the Democratic Republic of Congo: A Cross-Sectional Study[99]	Self-reported Adherence	No	Translated into French or Lingala	Interview	No	None
Breast Nurse Intervention to Improve Adherence to Endocrine Therapy Among Breast Cancer Patients in South Ethiopia [27]	SMAQ	No	Translated from English to Amharic	Interview	No	Monitoring of drug refill registration forms (Medication Possession Ratio - MPR
Prevalence and determinants of adherence to antiretroviral treatment among HIV patients on first-line	SMAQ	No	Not specified	Interview	No	Self-reporting tool and Visual

regimen: a cross-sectional study in Dakar, Senegal [103]						Analogue Scale (VAS)
The role of health facility and individual level characteristics on medication adherence among PLHIV on second-line antiretroviral therapy in Northeast Ethiopia: use of multi-level model[44]	SMAQ	No	Not specified	Interview	No	None
Medication non-adherence and its associated factors among kidney transplant patients in a large teaching hospital in Ethiopia[38]	SMAQ	No	Amharic	Interview	No	None
Using a Self-Administered Electronic Adherence Questionnaire to Identify Poor Adherence Amongst Adolescents and Young Adults on First-Line Antiretroviral Therapy in Johannesburg, South Africa[95]	SMAQ and South African National Department of Health (NDoH) adherence questionnaire	No	Not specified	Self-administered electronic questionnaire	Yes for NDoH and no for SMAQ	Viral Load Monitoring (Standard of Care) and Therapeutic Drug Monitoring (TDM)
General self-efficacy and hypertension treatment adherence in Algerian private clinical settings [98]	Treatment Adherence Scale	No	Not specified	Self-administered questionnaires	Yes	None

Muchanga IJ, *et al. BMJ Open* 2026; 16:e112898. doi: 10.1136/bmjopen-2025-112898
