# Supplementary material for: Mapping medication adherence tools in Africa: a scoping review
Source: BMJ Open. 2026 Sep 16;16(9):e112898. doi: 10.1136/bmjopen-2025-112898 (PMC13584023; doi:10.1136/bmjopen-2025-112898)
Supplement: Supplementary file 5 [file bmjopen-2025-112898.draft_revisions.pdf]

BMJ Open is committed to open peer review. As part of this commitment we make the peer review history of every article we publish publicly available.

When an article is published we post the peer reviewers' comments and the authors' responses online. We also post the versions of the paper that were used during peer review. These are the versions that the peer review comments apply to.

The versions of the paper that follow are the versions that were submitted during the peer review process. They are not the versions of record or the final published versions. They should not be cited or distributed as the published version of this manuscript.

BMJ Open is an open access journal and the full, final, typeset and author-corrected version of record of the manuscript is available on our site with no access controls, subscription charges or pay-per-view fees (http://bmjopen.bmj.com).

If you have any questions on BMJ Open's open peer review process please email info.bmjopen@bmj.com

BMJ Open

Mapping Medication Adherence Tools in Africa: A Scoping Review

Journal:	*BMJ Open*
Manuscript ID	bmjopen-2025-112898
Article Type:	Original research
Date Submitted by the Author:	25-Oct-2025
Complete List of Authors:	Muchanga, Izaidino; University of Porto, Department of Community Medicine; University of Porto, Center for Health Technologies and Services Research; São Tomás University of Mozambique<br>Midão, Luís ; University of Porto, RISE-Health<br>Mazuze, Avelino ; University of Porto, Department of Community Medicine; University of Porto, Center for Health Technologies and Services Research; Lúrio University, Faculty of Health Sciences<br>Melo, Daniela ; University of Porto, RISE-Health<br>Alcântara, Leovaldo ; University of Porto, RISE-Health<br>Figueiredo, Teodora ; University of Porto, RISE-Health<br>Costa, Elisio ; Universidade do Porto Faculdade de Farmacia, Biological Sciences<br>Sousa-Pinto, Bernardo; University of Porto
Keywords:	Chronic Disease, Clinical Decision-Making, INFECTIOUS DISEASES

SCHOLARONE MANUSCRIPTS

I, the Submitting Author has the right to grant and does grant on behalf of all authors of the Work (as defined in the below author licence), an exclusive licence and/or a non-exclusive licence for contributions from authors who are: i) UK Crown employees; ii) where BMJ has agreed a CC-BY licence shall apply, and/or iii) in accordance with the terms applicable for US Federal Government officers or employees acting as part of their official duties; on a worldwide, perpetual, irrevocable, royalty-free basis to BMJ Publishing Group Ltd ("BMJ") its licensees and where the relevant Journal is co-owned by BMJ to the co-owners of the Journal, to publish the Work in this journal and any other BMJ products and to exploit all rights, as set out in our licence.

The Submitting Author accepts and understands that any supply made under these terms is made by BMJ to the Submitting Author unless you are acting as an employee on behalf of your employer or a postgraduate student of an affiliated institution which is paying any applicable article publishing charge ("APC") for Open Access articles. Where the Submitting Author wishes to make the Work available on an Open Access basis (and intends to pay the relevant APC), the terms of reuse of such Open Access shall be governed by a Creative Commons licence – details of these licences and which Creative Commons licence will apply to this Work are set out in our licence referred to above.

Other than as permitted in any relevant BMJ Author's Self Archiving Policies, I confirm this Work has not been accepted for publication elsewhere, is not being considered for publication elsewhere and does not duplicate material already published. I confirm all authors consent to publication of this Work and authorise the granting of this licence.

For peer review only

Mapping Medication Adherence Tools in Africa: A Scoping Review

Izaidino Muchanga[1,2,5], Luís Midão[3], Avelino Mazuze[1,2,4], Daniela Melo[3], Leovaldo Alcântara[3], Teodora Figueiredo[3], Elísio Costa[3], Bernardo Sousa-Pinto[1,2]

[1] Department of Community Medicine, Information and Health Decision Sciences, Faculty of Medicine, University of Porto, Porto, Portugal
[2] Center for Health Technologies and Services Research, Health Research Network, University of Porto, Porto, Portugal
[3] RISE-Health, Competence Centre for Active and Healthy Ageing, Faculty of Pharmacy, University of Porto, Porto, Portugal
[4] Faculty of Health Sciences, Lúrio University, Nampula, Mozambique
[5] São Tomás University of Mozambique, Xai-Xai, Mozambique

Correspondence email:izaidino@yahoo.com

For peer review only

Abstract

Treatment adherence is essential for disease control and to achieve good clinical outcomes. This is even more important in the African context, where the population lives with a high burden of chronic and infectious diseases. The lack of systematisation on the tools used to measure adherence and whether these really apply to local realities has led to challenges in accurately assessing treatment outcomes. This scoping review aimed to map and characterise the tools used to assess adherence in African populations with chronic diseases. The study was conducted according to PRISMA-ScR guidelines and the methodological model of Arksey and O'Malley, refined by Levac et al. The search was conducted in PubMed, Scopus and Web of Science databases, without date restriction, but limited to peer-reviewed articles published in English. Studies that used or validated instruments to measure adherence to treatment in African countries, across all health settings and age groups were included. Grey literature, conference summaries, editorials and studies outside the African context or focused on acute diseases were excluded. Eighty-eight studies involving 26 African countries were analysed and the most used adherence tool was MMAS-8 in 44 studies. Other scales included MMAS-4, MARS, BMQ, SMAQ, GMAS, MAQ and CQR and indirect methods like self-reporting, electronic monitoring and pharmacy registers. The heterogeneity of the instruments showed the absence of standardisation in the measurement of adherence. The most studied chronic conditions were type 2 diabetes, hypertension, HIV and epilepsy, with 27; 20; 11 and 9 studies, respectively. Other studies have addressed psychiatric, rheumatic, respiratory, cardiovascular, renal and ophthalmological diseases and populations with multiple comorbidities. Most of the studies were conducted in Ethiopia and Nigeria, followed by Egypt, Kenya and South Africa. Countries such as Mozambique, Togo, Gabon, Guinea and the Democratic Republic of the Congo had limited representation, usually with a single study. The results demonstrated a wide range of scales used with the predominance of instruments developed outside Africa, which were not always validated for local socio-cultural contexts. This gap undermines the reliability of measurements and hinders formulation of effective interventions. It is concluded that there is an urgent need to develop and validate culturally adapted therapeutic adherence assessment tools for African contexts in order to improve the measurement of membership and to guide more effective public health policies.

Keywords: Adherence scales, adherence to treatment, chronic diseases and Africa

Introduction

Medication adherence usually refers to whether patients take their medications as prescribed, as well as whether they continue to take a prescribed medication (1). Medication adherence behavior has thus been divided into two main concepts, namely, adherence and persistence(2). Although conceptually similar, adherence refers to the intensity of drug use during the duration of therapy, whereas persistence refers to the overall duration of drug therapy.

Adherence to prescribed treatment regimens is essential for achieving successful therapeutic outcomes, especially in chronic disease management (to enhance chronic disease prognosis and reduce long-term healthcare expenditures). Non-adherence represents a substantial public health issue that negatively impacts clinical outcomes, patients' quality of life, and healthcare costs (3). For example, more than 30% of global hospital admissions are attributed to medication non-adherence (4). It is estimated that around 50% of patients with chronic diseases in developed countries do not adhere adequately to their treatment, with even higher non-adherence rates reported in developing countries such as Eswatini (27.7%), Botswana (24.1%), and South Africa (13.5%) (5). In fact, studies performed in African countries indicate non-adherence rates ranging from 20% to 40%, significantly compromising the effectiveness of treatment programs (6). Common barriers include transportation costs, income loss due to time spent attending medical appointments, long waiting times, and medication shortages (7). Additionally, healthcare professionals' attitudes and cultural norms can negatively influence adherence, with patients often fearing judgment for failing to strictly follow treatment regimens (8).

Achieving effective adherence is complex and requires an in-depth understanding of the underlying causes of non-adherence. Globally, more than 30% of hospital admissions are attributed to medication non-adherence (4).In Africa, these barriers are further exacerbated by socioeconomic factors such as poverty, cultural beliefs and traditional practices, and systemic issues including limited healthcare access and frequent medication shortages (9). Health systems in many regions also face resource scarcity and inequalities in healthcare access, compounding these challenges (10).

For peer review only

Sub-Saharan Africa bears a high burden of both infectious and chronic diseases, including human immunodeficiency virus / acquired immune deficiency syndrome (HIV/AIDS) (25%), tuberculosis (24%), hypertension (46%), and diabetes (8.5%). Studies indicate non-adherence rates ranging from 20% to 40%, significantly compromising the effectiveness of treatment programs (6). Common barriers include transportation costs, income loss due to time spent attending medical appointments, long waiting times, and medication shortages (7). Additionally, healthcare professionals' attitudes and cultural norms can negatively influence adherence, with patients often fearing judgment for failing to strictly follow treatment regimens (8).

Appropriate tools are critical to quantify adherence. Various methodologies exist, including direct methods such as detecting drugs or metabolites in biological samples, and indirect methods such as pill counts, pharmacy refill records, electronic monitoring devices, and self-reported questionnaires (3). Selecting the most suitable tool requires consideration of the clinical context and population characteristics, particularly in African settings where cultural and socioeconomic factors may influence assessment outcomes. However, despite the importance of adherence assessment in African countries, there appears to be a scarcity of validated and culturally adapted scales. The most frequent adherence scales, such as the Morisky-Green scale (4- and 8-item versions), the Medication Adherence Rating Scale (MARS), and the Medication Compliance Questionnaire (MCQ) (11), were developed and validated outside Africa, raising concerns about their direct applicability in African populations due to cultural and socioeconomic differences. The existence of adherence scales suitable to be used in the African context would allow for more accurate measurement of non-adherence and for the design of effective interventions (12). Therefore, the objective of this scoping review is to map and characterize adherence assessment tools that have been used and validated in various African settings.

Methods

This scoping review was conducted following the Preferred Reporting Items for Systematic Reviews and Meta-Analyses extension for Scoping Reviews (PRISMA-ScR) guidelines (13). The methodological approach was based on the framework proposed by Arksey and O'Malley(14), further refined by Levac et al (15), and included: identification

For peer review only

of the research question, to explore and map the tools available for assessing medication adherence across Africa; identification of relevant studies through a systematic search strategy across multiple electronic databases and grey literature sources; selection of studies using predefined inclusion and exclusion criteria based on relevance to the topic and objectives; data extraction using a structured form to collect key characteristics, types of adherence tools, settings, populations, and implementation contexts; and synthesis of results through narrative and thematic approaches, highlighting patterns, gaps, and methodological diversity among the identified tools to ensure transparency and reproducibility.

A scoping review of literature was chosen because it allows for a comprehensive and detailed mapping of the various medication adherence tools used in Africa, a field that is still emerging and methodologically diverse. This approach facilitates the identification of knowledge gaps and conceptual differences, providing an essential foundation for future research and policies tailored to the complexity and heterogeneity of African contexts.

Research Question

The research question of this review was centered on the identification of validated tools, scales, or instruments used to assess medication adherence in African countries. The review sought to provide a comprehensive mapping of available measures and their use across diverse populations and healthcare contexts within the continent.

Eligibility Criteria

Studies were included if they involved populations of any age from African countries and described the use or validation of instruments to measure medication adherence. The review considered all healthcare settings and focused exclusively on peer-reviewed articles published in English and French. There were no limitations on the year of publication.
Grey literature, conference abstracts, opinion pieces, editorials, and other non-peer-reviewed sources were excluded

Information Sources and Search Strategy

The literature search was carried out across three major databases: PubMed, Scopus, and Web of Science. The final search was completed on December 10, 2024. Search terms

For peer review only

were selected based on MeSH terms and relevant keywords to ensure comprehensive coverage of the topic. The search strategy combined geographic keywords related to African countries with terms describing medication adherence (e.g., "medication compliance", "treatment nonadherence") and terms related to measurement instruments (e.g., "scale", "tool", "survey", "assessment"). Boolean operators like AND, OR, and NOT were used to combine and refine the search terms. No filters were applied regarding publication date or study type beyond the language and peer-review criteria. The complete search strategy is available in the supplementary materials.

Study Selection Process

The screening process was carried out in three sequential stages: initial screening of titles, followed by abstracts, and subsequently full-text articles. Two reviewers independently assessed the eligibility of studies at each stage. Any discrepancies were resolved through consultation with a third reviewer to reach consensus. The screening and selection process was facilitated using Rayyan (16), a web-based tool designed to streamline systematic and scoping review workflows.

Data Extraction

Data extraction was performed independently by two reviewers (I.M and L.M) using a structured extraction form developed for this review. Any disagreements between the reviewers were resolved through discussion and consensus. The form was piloted to ensure clarity and consistency. Extracted data included the article title, authorship and year of publication, study country, characteristics of the population (including sample size, gender distribution, and mean age), setting, study design, the name and nature of the adherence scale or tool, the associated disease or condition, and reported adherence rates when available.

For peer review only

Data Analysis and Synthesis

The findings were synthesised descriptively and organised into tables to facilitate interpretation and comparison. Given the scope of the review, no subgroup analyses or thematic categorisations were planned or conducted.

Quality Assessment

Consistent with the objectives and nature of a scoping review, no formal assessment of methodological quality or risk of bias was undertaken.

Ethics Considerations

This review did not involve the collection of primary data or engagement with human participants and therefore did not require ethical approval.

For peer review only

Results

In total, 6,541 records were identified from the following databases: PubMed (n = 631), Web of Science (n = 3,514), and Scopus (n = 2,369) (Figure 1). Before the initial screening, 1,866 duplicate records were removed, leaving 4,675 records for the screening stage. No records were excluded by automation tools or for other reasons at this stage. During the screening of the 4,675 records, 4,400 records were excluded for not meeting the established inclusion criteria. Consequently, 275 full-text reports were selected for retrieval and detailed eligibility assessment. After this stage, 88 studies – performed in 26 African countries – met all eligibility criteria and were included in the review.

For peer review only

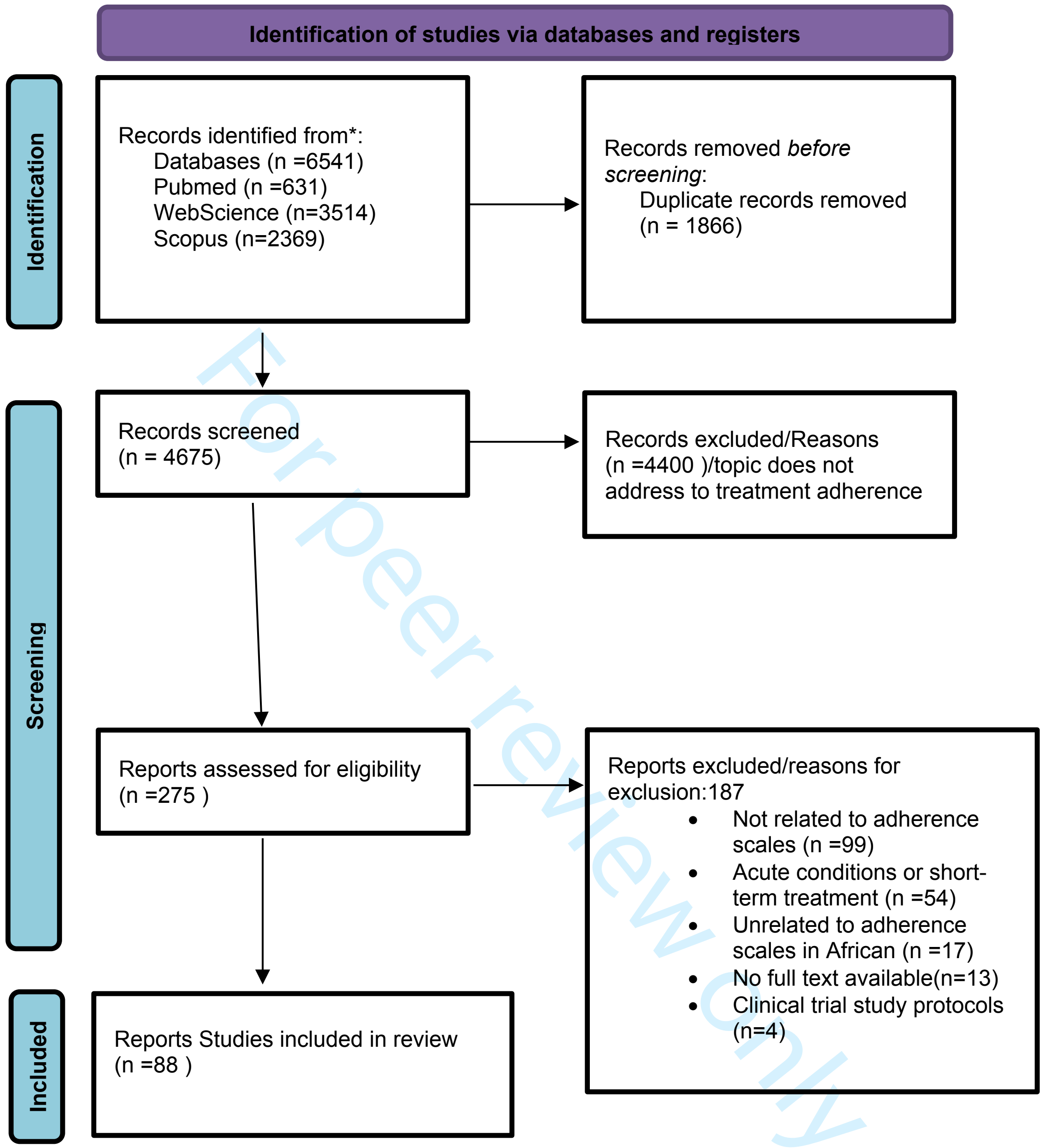

Figure 1: PRISMA flow chart diagram for scoping review on Mapping Medication Adherence Tools in Africa

For peer review only

Study characteristics

Population Demographics

Demographically, the average population size across the studies was approximately 449 participants, exhibiting substantial variation (minimum of 67 and maximum of 4489). The average percentage of female participants was 57.0%. The average age of participants was approximately 49.8 years, with a range of 6.9 to 69.7 years. The reported medication adherence rates varied widely, with an average of 66.5%.

Geographical Distribution

The studies included in this scoping review were conducted across a range of African countries, reflecting the regional diversity of adherence research on chronic conditions. According to the map of Africa (figure 2)the majority of studies were conducted in Ethiopia (30 studies) (17-46) and Nigeria (22 studies) (47-69), highlighting these countries as key contributors to the evidence base. Egypt accounted for 5 studies (40, 70-74), while Kenya and Ghana were the setting for 4 studies (75-82), Sudan and Cameron were the setting for 3 studies (83-87). South Africa, Uganda, Senegal, Benim, Togo and Algeria each contributed 2 studies (79-91). Single studies were conducted in Eritrea, Rwanda, Libya, Benin, Togo, Tanzania, Mozambique, Gabon, Guinea, Côte d'Ivoire, Mauritania, Niger, Democratic Republic of Congo, and the Republic of Congo (92-99). Additionally, one multicountry study spanned Benin, Cameroon, Congo, Democratic Republic of Congo, Gabon, Guinea, Côte d'Ivoire, Mauritania, Mozambique, Niger, Senegal, and Togo (100).

For peer review only

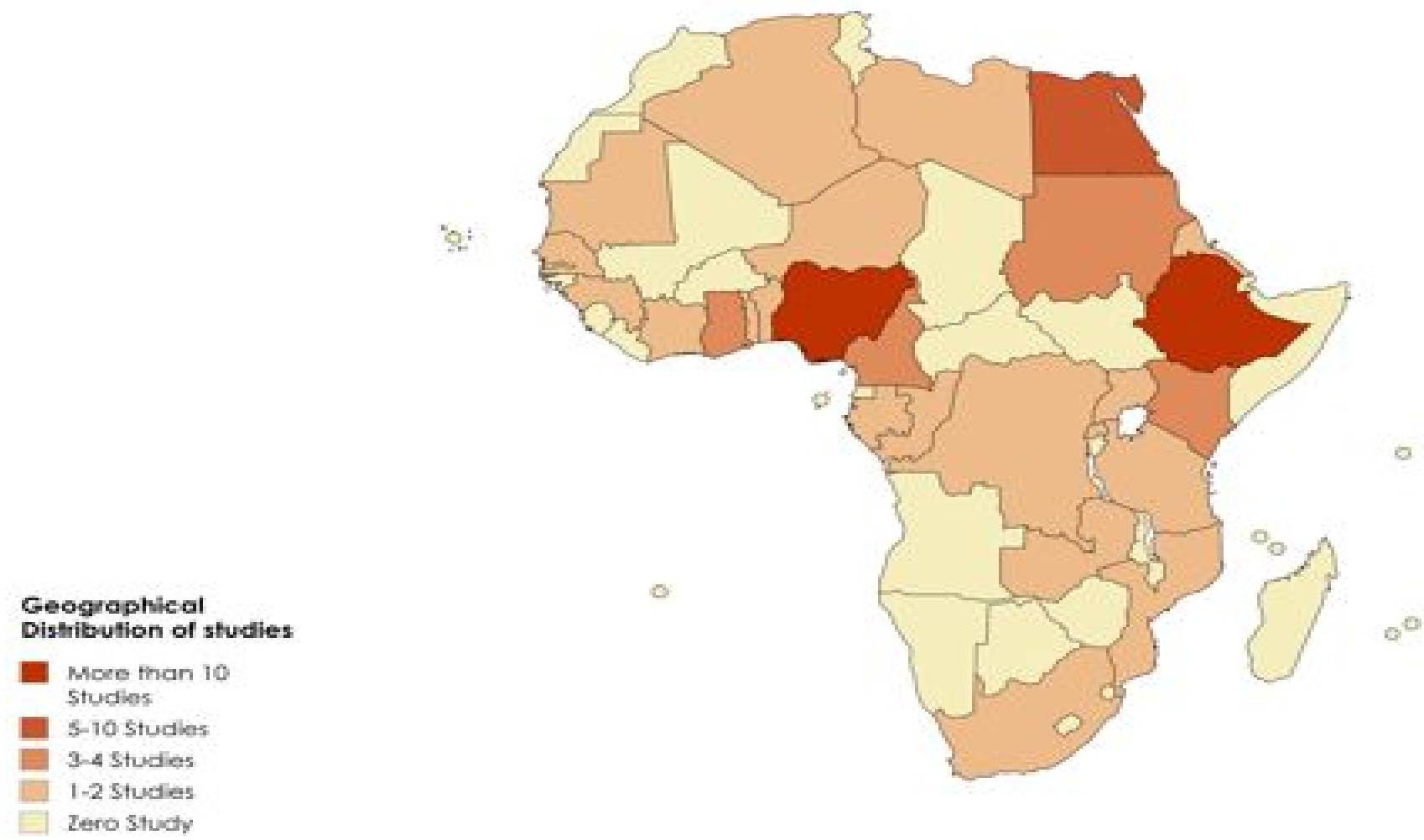

Figure 2:Map of Geographical Distribution of the Studies in Africa

Disease Conditions

The studies included in this systematic review covered a wide range of chronic conditions. The most frequently investigated condition was type 2 diabetes mellitus, reported in 27 studies (18, 29, 34, 35, 37, 38, 44, 55, 59-61, 67, 71-73, 78, 81, 82, 87, 90, 93-96, 98, 101), followed by hypertension, assessed in 17 studies (17, 23, 30, 36, 47, 49, 56, 57, 66, 68, 69, 76, 77, 83, 91, 99, 100, 102). HIV was examined in 11 studies (45, 48, 58, 63, 75, 77, 80, 89, 92, 97, 103), while epilepsy was the focus of 7 studies (19-22, 26, 84, 88). Other conditions addressed included type 1 diabetes mellitus (6 studies) (29, 35, 38, 55, 93, 101), depressive disorders (5 studies) (32, 33, 43, 52, 65), schizophrenia (4 studies) (24, 25, 42, 54), asthma (2 studies) (41, 86), glaucoma (3 studies) (46, 50, 79), heart failure (2 studies) (40, 104), rheumatoid arthritis (2 studies) (53, 85), bipolar disorder (1 study) (70), breast cancer (1 study) (28), chronic kidney disease (1 study) (51), Behçet's disease (1 study) (74), kidney transplant recipients (1 study) (39), and psychiatric out-patients (1 study) (62). Additionally, several studies examined adherence in populations with mixed chronic illnesses or complex comorbidities, such as combinations of hypertension, diabetes mellitus, arthritis, cardiac disease, renal disease, and mental illness (33, 65, 83, 105).

Adherence Assessment Tools

Within the 88 studies included in this scoping review (Table 1), a variety of adherence measurement tools were employed. The most frequently used instrument was the 8-item Morisky Medication Adherence Scale (MMAS-8), applied in 45 studies (17, 19, 21, 23, 27, 29-31, 35, 38, 43, 44, 46, 47, 49, 55-58, 63, 65-67, 70-73, 76, 78, 80, 83, 85, 86, 88, 90, 94, 95, 98-100). Other versions of the Morisky scale were also reported, including the 4-item MMAS (MMAS-4, used in 5 studies) (18, 42, 84, 96, 104), the 9-item MMAS (MMAS-9), 1 study (68), and unspecified MMAS versions (5 studies) (22, 26, 50, 51, 54). The Medication Adherence Report Scale (MARS) was used in 9 studies (24, 25, 32, 33, 41, 52, 60, 82, 102), either alone or combined with other tools. The 5-item MARS (MARS-5) was employed in 5 studies (20, 34, 40, 53, 79). The Brief Medication Questionnaire (BMQ) appeared in 2 studies, sometimes in combination with MARS or MMAS (61, 93). Additional tools included the General Medication Adherence Scale (GMAS, 2 studies) (37, 105), the Simplified Medication Adherence Questionnaire (SMAQ, 5 studies) (28, 39, 45, 89, 97), the Medication Adherence Questionnaire (MAQ, 1 study) (81), the Patient Medication Adherence Questionnaire (PMAQ, 1 study) (75), the Compliance Questionnaire on Rheumatology (CQR, 1 study) (74), the Medication Adherence Scale (MAS-8, 1 study) (100), and several studies relied on self-reported adherence, electronic medication monitoring (Mems), pharmacy refill data, or combined methods (62, 77, 91, 92, 103).

Within the 88 studies included in this scoping review, only 12(only 13.6% of the studies) reported having undertaken translation and/or cultural adaptation of the instruments to the local context. Among the included studies, six validated the MMAS-8 across different African countries(21, 46, 83, 94, 98, 99), while one study validated the MMAS (50), two validated the MARS-5 (79), one validated the MARS (52, 102), one validated the MAQ (81), and one validated the CQR (74).

Medication Adherence Rates

Medication adherence rates (table 1) showed wide variation across different diseases analyzed in studies conducted in African countries. Overall, adherence to treatment was

For peer review only

found to be strongly influenced by the type of disease, population context, assessment tools used, and individual patient characteristics.

In the case of asthma, adherence rates ranged from 24.5% to 44.8%, indicating generally low adherence to controller therapy. Among individuals with mixed chronic diseases, values ranged from 48.6% to 50.6%, reflecting moderate levels of therapeutic compliance. For glaucoma, adherence ranged between 46.3% and 60.8%, while among patients with heart failure, rates varied from 36.4% to 76.3%, suggesting differences associated with clinical severity and treatment regimens.

On HIV, studies reported adherence rates between 26.7% and 96.3%, with higher adherence observed among pediatric populations and lower rates among adolescents and young adults. In cases of arterial hypertension, adherence ranged widely from 3.0% to 96.8%, demonstrating substantial heterogeneity between countries and measurement methodologies.

For other conditions, a study on Behçet's disease reported an adherence rate of 69.2%, while bipolar I disorder showed considerably low adherence at only 19.0%. Among patients with breast cancer undergoing endocrine therapy, adherence reached 70.0%, and in cardiovascular diseases, a rate of 49.0% was observed. Patients with chronic kidney disease demonstrated an adherence rate of 48.6%, whereas in kidney transplant cases, adherence reached 81.9%, reflecting a strong commitment to immunosuppressive therapy.

In mental disorders, adherence ranged from 59.0% to 62.3% for general psychiatric conditions and was 44.3% in combined severe mental disorders. Among patients with depressive disorders, rates ranged from 9.5% to 74.0%, showing high interindividual variability. In epilepsy, adherence varied widely from 4.0% to 65.9%, reflecting specific challenges in disease management.

In rheumatologic conditions, rheumatoid arthritis showed the lowest adherence rates, ranging from 0.0% to 11.8%, indicating significant difficulties in treatment compliance. Schizophrenia exhibited intermediate adherence levels, with rates between 45.0% and 68.4%. In type 1 and type 2 diabetes mellitus, adherence rates were highly variable, ranging from 5.6% to 86.8%, with the lowest values reported in studies conducted in Nigeria and the highest in Ghanaian and Ethiopian contexts.

For peer review only

Table 1: Characteristics of Studies on Medication Adherence in Patients with Chronic Diseases in Africa

Title	Author & Year	Country	Disease	Population (n)	% of Female	Population Mean Age	Settings	Study Design	Adherence Scale Tool	Adherence Rate (%)
Evaluation of factors affecting adherence to asthma controller therapy in chest clinics in a sub-Saharan African setting: a cross-sectional study (86)	Ngahane et al.2016	Cameroon	Asthma	210	63.3	41.2	Hospital	Cross-Sectional	MMAS-8	44.8
Medication regimen complexity and its impact on medication adherence and asthma control among patients with asthma in Ethiopian referral hospitals (41)	Belachew et al.2022	Ethiopia	Asthma	396	60.1	49.4	Hospital	Cross-Sectional	MARS	24.5
Evaluation of treatment adherence in patients with Behçet's disease: its relation to disease manifestations, patients' beliefs about medications, and quality of life (74)	Zayed et al.2019	Egypt	Behçet's Disease	67	14.9	35.0	Hospital	Cross-Sectional	CQR	69.2
Psycho-demographic and clinical predictors of medication adherence in patients with bipolar I disorder in a university hospital in Egypt (70)	Okasha et al.2020	Egypt	Bipolar I Disorder	110	32.7	36.6	Hospital	Longitudinal Prospective	MMAS-8	19.0
Breast Nurse Intervention to Improve Adherence to Endocrine Therapy Among Breast Cancer Patients in South Ethiopia (28)	Getachew et al.2022	Ethiopia	Breast Cancer	162	100.0	41.8	Hospital	Cluster Randomized	SMAQ	70.0
Medication adherence among cardiac patients in Khartoum State, Sudan: a cross-sectional study (83)	Awad et al.2017	Sudan	Cardiovascular Diseases ((hypertension, dyslipidaemia, ischaemic heart disease, chronic heart failure, Arrhythmia, cardiac valve replacement, rheumatic heart disease, cerebrovascular disease)	386	57.0		Hospital	Cross-Sectional	MMAS-8	49.0
Medication adherence and its influencing factors in community-dwelling older adults with chronic illnesses in a rural area (105)	Ahmed et al.2023	Egypt	Chronic Illnesses (hypertension, cardiac disease, renal disease, arthritis, diabetes, neurological, hepatic, osteoporosis, respiratory, gastrointestinal, thyroid)	310	52.6	69.7	Hospital	Cross-Sectional	GMAS	50.6
Evaluation of medication adherence and predictors of sub-optimal adherence among pre-dialysis patients with chronic kidney disease (51)	Okoro et al.2020	Nigeria	Chronic Kidney Disease	220	61.4	52.7	Hospital	Prospective	MMAS	48.6
Impact of pharmacist intervention on antidepressant medication adherence and disease severity in patients with major depressive disorder in fragile north-east Nigeria (52)	Yusuf et al.2021	Nigeria	Depressive Disorders	101	80.2		Hospital	Prospective Interventional	MARS	9.5
Magnitude and factors associated with treatment non-adherence among patients with depressive disorders at St. Amanuel Mental Specialized	Alemayehu et al. 2022	Ethiopia	Depressive Disorders	415	53.0	36.0	Hospital	Cross-Sectional	MARS	74.0

For peer review only - http://bmjopen.bmj.com/site/about/guidelines.xhtml

Hospital, Addis Ababa, Ethiopia, 2019: A cross sectional study (32)										
Self-Reported Adverse Drug Reactions, Medication Adherence, and Clinical Outcomes among Major Depressive Disorder Patients in Ethiopia: A Prospective Hospital Based Study (43)	Abegaz et al. 2017	Ethiopia	Depressive Disorders	217	43.8	30.9	Hospital	Cross-Sectional	MMAS-8	10.6
Effect of patients beliefs about medications on adherence to drugs in diabetic patients attending family medicine outpatient clinic in Ismailia, Egypt (71)	Salama & Saudi.2020	Egypt	Diabetes Mellitus	82	85.4	18.6	Hospital	Cross-Sectional	MMAS-8	45.1
Medication belief as correlate of medication adherence among patients with diabetes in Edo State, Nigeria (61)	Olorunfemi and Ojewole.2019	Nigeria	Diabetes Mellitus	180	55.6	57.2	Hospital	Descriptive Correlational	BMQ and MMAS	5.6
Adherence to Treatment and Factors Affecting Adherence of Epileptic Patients at Yirgalem General Hospital, Southern Ethiopia: A Prospective Cross-Sectional Study (19)	Hasiso and Desse.2016	Ethiopia	Epilepsy	194	43.8	33.6	Hospital	Cross-Sectional	MMAS-8	32.0
Antiepileptic Drug Adherence and Its Associated Factors among Epilepsy Patients on Follow-ups at Amanuel Mental Specialized Hospital, Ethiopia (20)	Shumet et al.2022	Ethiopia	Epilepsy	439	35.3	16.4	Hospital	Cross-Sectional	MARS-5	16.4
Antiepileptic drug adherence in children in southern Ethiopia: A cross sectional study (21)	Dima and Shibesh.2022	Ethiopia	Epilepsy	192	33.3	6.9	Hospital	Cross-Sectional	MMAS-8	65.0
Antiepileptic Drug Nonadherence and Its Predictors among People with Epilepsy (22)	Getnet et al.2016	Ethiopia	Epilepsy	450	41.3	27.0	Hospital	Cross-Sectional	MMAS	62.2
Assessment of Patients' Adherence to Antiepileptic Medications at Dessie Referral Hospital, Chronic Follow-Up, South Wollo, Amhara Region, North East Ethiopia (26)	Kassahun et al.2018	Ethiopia	Epilepsy	88	37.5		Hospital	Cross-Sectional	MMAS	65.9
Beliefs about medication, medication adherence and seizure control amongadult epilepsy patients in Kimberley, South Africa (88)	Egenas et al.2015	South Africa	Epilepsy	197	39.1	40.0	Hospital	Descriptive Observational	MMAS-8	54.6
Factors affecting adherence to antiepileptic medications among Sudanese individuals with epilepsy: A cross-sectional survey (84)	Elsayed et al.2019	Sudan	Epilepsy	96	68.8	29.0	Hospital	Cross-Sectional	MMAS-4	4.0
Associations Between Personality Traits and Adherence to Treatment in Patients with Primary Open Angle Glaucoma in an African Population (79)	Antir et al.2021	Ghana	Glaucoma	400	52.8	56.2	Hospital	Cross-Sectional	MARS-5	60.8
Drug utilization, medication adherence and treatment satisfaction amongst glaucoma patients receiving care at secondary health facilities (50)	Bello et al.2023	Nigeria	Glaucoma	311	59.2	63.8	Hospital	Cross-Sectional	MMAS	46.3
Treatment adherence and associated factors among glaucoma patients attending Ophthalmic units of referral hospitals in North West Ethiopia, 2019 (46)	Birhanie et al.2022	Ethiopia	Glaucoma	382	30.9	57.7	Hospital	Cross-Sectional	MMAS-8	49.5

For peer review only

Medication Nonadherence and Associated Factors among Heart Failure Patients at University of Gondar Comprehensive Specialized Hospital, Northwest Ethiopia (40)	Seid et al.2023	Ethiopia	Heart Failure	245	66.5	47.8	Hospital	Cross-Sectional	MARS-5	76.3
Pharmacist intervention to improve adherence to medication among heart failure patients at North East Ethiopia hospital (104)	Tsige et al.2024	Ethiopia	Heart Failure	412	53.6	44.7	Hospital	Pre-Post Interventional	MMAS-4	36.4
Clinical Utility of Pharmacy-Based Adherence Measurement in Predicting Virologic Outcomes in an Adult HIV-Infected Cohort in Jos, North Central Nigeria (48)	Abah et al.2016	Nigeria	HIV	926	66.0	34.0	Hospital	Retrospective	Drug-Refill Visits	57.5
Factors contributing to antiretroviral drug adherence among adults living with HIV or AIDS in a Kenyan rural community (75)	Kioko et al.2017	Kenya	HIV	301	62.8	40.4	Hospital	Cross-Sectional	PMAQ	86.0
Food Insecurity Is Associated with Increased Risk of NonAdherence to Antiretroviral Therapy among HIV-Infected Adults in the Democratic Republic of Congo: A CrossSectional Study (92)	Musumari et al.2014	Democratic Republic of Congo	HIV	898	72.2	44.0	Hospital	Cross-Sectional	Self-reported Adherence	79.1
Incomplete adherence among treatment-experienced adults on antiretroviral therapy in Tanzania, Uganda and Zambia (103)	Denison et al.2015	Tanzania, Uganda And Zambia	HIV	4489	68.0	40.0	Hospital	Cross-Sectional	Self-report, provider report and pharmacy refill	85.5
Measuring adherence to antiretroviral therapy in children and adolescents in western Kenya (77)	Vreeman et al.2014	Kenya	HIV	210	100.0	8.2	Hospital	Prospective Cohort	Electronic Medication Monitoring Using Mems	96.3
Medication adherence in HIV-positive pregnant women on antiretroviral therapy attending antenatal clinics in ado metropolis, south-west nigeria: A multicentre study (58)	Aduloju et al.2020	Nigeria	HIV	170	100.0	29.8	Hospital	Cross-Sectional	MMAS-8	75.3
Mood and anxiety disorders adversely influence medication adherence to antiretroviral therapy among people living with HIV/AIDS in Nigeria (63)	Omonefe Seb-Akahomen et al.2018	Nigeria	HIV	410	75.9	40.4	Hospital	Cross-Sectional	MMAS-8	63.2
Prevalence and determinants of adherence to antiretroviral treatment among HIV patients on first-line regimen: a cross-sectional study in Dakar, Senegal (97)	Mbengue et al.2019	Senegal	HIV	150	75.3	43.0	Hospital	Cross-Sectional	SMAQ and Self-reporting tool	30.6
Socio-demographic factors associated with medication adherence among People Living with HIV in the Kumasi Metropolis, Ghana (80)	Adu et al.2022	Ghana	HIV	420	80.0	45.0	Hospital	Cross-Sectional	MMAS-8	53.1
The role of health facility and individual level characteristics on medication adherence among PLHIV on second-line antiretroviral therapy in Northeast Ethiopia: use of multi-level model (45)	Wedajo et al.2022	Ethiopia	HIV	714	56.0		Hospital	Cross-Sectional	SMAQ	69.5

For peer review only

Using a Self-Administered Electronic Adherence Questionnaire to Identify Poor Adherence Amongst Adolescents and Young Adults on First-Line Antiretroviral Therapy in Johannesburg, South Africa (89)	Hirasen et al. 2020	South Africa	HIV	278	78.8	31.0	Hospital	Cross-Sectional	Self-reported Adherence tool and SMAQ	26.7
Adherence to antihypertensive medication and its correlates among individuals with hypertension in a semi-urban community of Southern Nigeria (47)	Okwuonu et al.2015	Nigeria	Hypertension	252	43.3	56.6	Hospital	Cross-Sectional	MMAS-8	31.8
Adherence to Antihypertensive Treatment and Associated Factors in Central Ethiopia (17)	Tsadik , Berhane ,and Worku. 2020	Ethiopia	Hypertension	989	52.7	57.6	Hospital	Cross-Sectional	MMAS-8	36.0
Antihypertensive medication adherence and associated factors among adult hypertensive patients at Jimma University Specialized Hospital, southwest Ethiopia (23)	Asgedom et al.2018	Ethiopia	Hypertension	280	46.8	57.7	Hospital	Cross-Sectional	MMAS-8	61.8
Antihypertensive Medication Adherence and Associated Factors: A Cross-Sectional Analysis of Patients Attending a National Referral Hospital in Asmara, Eritrea (102)	Mebrahtu et al.2021	Eritrea	Hypertension	335	66.3	59.7	Hospital	Cross-Sectional	MARS	27.2
Factors associated with poor adherence to medication among hypertensive patients in twelve low and middle income Sub-Saharan countries (100)	Terline et al.2019	Benin, Cameroon, Congo, Democratic Republic of Congo, Gabon, Guinea, Costa de Marfim, Mauritania, Mozambique, Niger, Senegal and Togo	Hypertension	2198	88.2	57.7	Hospital	Cross-Sectional	Medication Adherence Scale-8 (MAS-8)	30.8
Family support and medication adherence among residents with hypertension in informal settlements of Nairobi, Kenya: a mixed-method study (76)	Xiong et al.2023	Kenya	Hypertension	93	65.6	57.0	Urban Settlement	Mixed-Methods	MMAS-8	43.0
General self-efficacy and hypertension treatment adherence in Algerian private clinical settings (91)	Said Kara.2022	Algeria	Hypertension	216	52.8	25.3	Hospital	Cross-Sectional	Treatment Adherence Scale(91)	
Impact of adverse drug events and treatment satisfaction on patient adherence with antihypertensive medication – a study in ambulatory patients (30)	Berhe et al.2017	Ethiopia	Hypertension	925	61.6	57.0	Hospital	Cross-Sectional	MMAS-8	21.0
Medication adherence and its associated factors among hypertensive patients attending the	Teshome et al.2017	Ethiopia	Hypertension	337	51.6	58.3	Hospital	Cross-Sectional	MGLS	75.1

Debre Tabor General Hospital, northwest Ethiopia (36)										
Medication Adherence and Patient Satisfaction among Hypertensive Patients Attending Outpatient Clinic in Lagos University Teaching Hospital, Nigeria (56)	Oluwole et al.2019	Nigeria	Hypertension	500	56.8	58.9	Hospital	Cross-Sectional	MMAS-8	90.2
Medication Adherence and Perceived Family Support Among Elderly Patients with Hypertension Attending a Specialty Clinic in Lagos, Nigeria (57)	Olaniran et al.2023	Nigeria	Hypertension	293	73.0	69.3	Hospital	Cross-Sectional	MMAS-8	14.3
Relationship between Cost of Care and Medication Adherence among Hypertensive Patients Attending a Tertiary Health Facility in Ekiti State, Nigeria (66)	Ipinnimo et al.2022	Nigeria	Hypertension	138	54.4	52.6	Hospital	Cross-Sectional	MMAS-8	34.1
Treatment adherence and blood pressure outcome among hypertensive out-patients in two tertiary hospitals in Sokoto, Northwestern Nigeria (68)	Adisa et al.2018	Nigeria	Hypertension	605	59.2	54.5	Hospital	Cross-Sectional	MMAS-9	8.9
Treatment satisfaction and medication adherence among hypertensive patients in a teaching hospital in Ekiti State, Nigeria (69)	Akpor and Olowolaju.2022	Nigeria	Hypertension	270	63.0		Hospital	Cross-Sectional	MMAS-8	9.3
Validity and Reliability of a Self-Reported Measure of Antihypertensive Medication Adherence in Uganda (99)	Okello et al.2016	Uganda	Hypertension	329	69.3	55.0	Hospital	Cross-Sectional	MMAS-8	3.0
Depression and medication-adherence in patients with hypertension attending a tertiary health facility in South-West Nigeria (49)	Okunrinboye et al.2019	Nigeria	Hypertension And Depression	400	62.0	59.6	Hospital	Cross-Sectional	MMAS-8	96.8
Magnitude and associated factors of poor medication adherence among diabetic and hypertensive patients visiting public health facilities in Ethiopia during the COVID-19 pandemic (31)	Shimels et al.2021	Ethiopia	Hypertension and Diabetes Mellitus	409	56.0	56.5	Hospital	Cross-Sectional	MMAS-8	28.0
Medication non-adherence and its associated factors among kidney transplant patients in a large teaching hospital in Ethiopia (39)	Derejie et al.2024	Ethiopia	Kidney Transplant	270	28.5	37.0	Hospital	Cross-Sectional	SMAQ	81.9
Medication adherence and associated factors among psychiatry patients at Asella Referral and Teaching Hospital in Oromia, Ethiopia: Institution based cross sectional study (33)	Gudeta et al.2023	Ethiopia	Mental Illness (Major depressive disorder, schizophrenia, bipolar disorders, Brief psychotic disorder and schizophreniform disorder)	395	58.0	33.0	Hospital	Cross-Sectional	MARS	62.3
Medication compliance behavior in psychiatric out-patients with psychoactive substance use comorbidity in a Nigerian tertiary hospital (62)	Okpataku et al.2015	Nigeria	Psychiatric Out-Patient	208	47.1	36.7	Hospital	Cross-Sectional	Self-Administered Medication Adherence Scale	59.0

Medication adherence among Nigerian patients with rheumatoid arthritis: a two instruments survey (53)	Ubaka et al.2021	Nigeria	Rheumatoid Arthritis	169	62.7		Hospital	Cross-Sectional	MARS-5	11.8
Status of rheumatoid arthritis practice and treatment in Sudan (85)	Ali et al. 2023	Sudan	Rheumatoid Arthritis	90	93.3	55.0	Hospital	Cross-Sectional Descriptive	MMAS-8	0.0
Antipsychotic medication non-adherence among schizophrenia patients in Central Ethiopia (24)	Tareke et al. 2018	Ethiopia	Schizophrenia	412	30.3	35.3	Hospital	Cross-Sectional	MARS	59.0
Antipsychotic medication non-adherence and factors associated among patients with schizophrenia in eastern Ethiopia (25)	Mohammed et al.2024	Ethiopia	Schizophrenia	893	18.4	34.2	Hospital	Cross-Sectional	MARS	59.0
Medication adherence among Nigerians with schizophrenia: correlation between clinico-demographic factors and quality of life (54)	Ogunnubi et al.2017	Nigeria	Schizophrenia	160	53.8	38.5	Hospital	Cross-Sectional	MMAS	45.0
Quality of life and its association with current substance use, medication nonadherence and clinical factors of people with schizophrenia in Southwest Ethiopia: a hospital-based cross-sectional study (42)	Desalegn et al. 2020	Ethiopia	Schizophrenia	351	31.1	33.0	Hospital	Cross-Sectional	MMAS-4	68.4
Prevalence and predictors of sub-optimal medication adherence among patients with severe mental illnesses in a tertiary psychiatric facility in Maiduguri, North-eastern Nigeria (65)	Ibrahim et al.2015	Nigeria	Severe Mental Illnesses (schizophrenia, bipolar disorder or severe depression)	370	43.5	35.0	Hospital	Cross-Sectional	MMAS-8	44.3
Barriers to diabetes medication adherence in North West Ethiopia (101)	Abebe et al.2014	Ethiopia	Type 1 and Type 2 Diabetes Mellitus	391	52.9	50.4	Hospital	Cross-Sectional	MMAS-8	71.2
Drug therapy problems, medication adherence and treatment satisfaction among diabetic patients on follow-up care at Tikur Anbessa Specialized Hospital, Addis Ababa, Ethiopia Gebre Teklemariam D (29)	Demoz et al.2019	Ethiopia	Type 1 and Type 2 Diabetes Mellitus	418	52.9	53.0	Hospital	Cross-Sectional	MMAS-8	45.7
Health literacy, medication belief, and demographic factors as correlates of medication adherence in person with diabetes mellitus (93)	Olorunfemi and Olaolorunpo.2023	Benin	Type 1 and Type 2 Diabetes Mellitus	180	55.6	57.2	Hospital	Descriptive Correlational	MARS and BMQ	5.6
Medication adherence and direct treatment cost among diabetes patients attending a tertiary healthcare facility in Ogbomosho, Nigeria (55)	Fadare et al.2015	Nigeria	Type 1 and Type 2 Diabetes Mellitus	129	60.5	61.4	Hospital	Cross-Sectional	MMAS-8	40.6
Medication adherence and its associated factors among diabetic patients at Zewditu Memorial Hospital, Addis Ababa, Ethiopia (35)	Muhammed et al.2017	Ethiopia	Type 1 and Type 2 Diabetes Mellitus	146	54.1	46.5	Hospital	Cross-Sectional	MMAS-8	54.8
Medication non-adherence and associated factors among diabetes patients in Felege Hiwot Referral Hospital, Bahir Dar city administration, Northwest Ethiopia (38)	Teshager and Abate.2019	Ethiopia	Type 1 and Type 2 Diabetes Mellitus	416	41.8	45.4	Hospital	Cross-Sectional	MMAS-8	31.2

Adherence to antidiabetic medication and factors associated with non-adherence among patients with type-2 diabetes mellitus in two regional hospitals in Cameroon (87)	Aminde et al.2019	Cameroon	Type 2 Diabetes Mellitus	195	69.7		60.5	Hospital	Cross-Sectional	MCQ	45.6
Adherence to Oral Antidiabetic Medications among Type 2 Diabetic (T2DM) Patients in Chronic Ambulatory Wards of Hiwot Fana Specialized University Hospital, Harar, Eastern Ethiopia: A Cross Sectional Study (18)	Jemal et al.2017	Ethiopia	Type 2 Diabetes Mellitus	196	45.4		61.1	Hospital	Cross-Sectional	MMAS-4	70.4
Association of treatment satisfaction and physician trust with glycemic control among primary care patients with type 2 diabetes in Egypt (73)	Ahmed et al.2024	Egypt	Type 2 Diabetes Mellitus	319	57.1		59.7	Hospital	Cross-Sectional	MMAS-8 and MKQ	79.9
Factors associated with medication non-adherence in patients with type 2 diabetes mellitus: The first Algerian survey (90)	Achouri et al.2019	Algeria	Type 2 Diabetes Mellitus	403		0	60.0	Hospital	Cross-Sectional	MMAS-8	68.7
Illness perceptions of Libyans with T2DM and their influence on medication adherence: a study in a diabetes center in Tripoli (94)	Ashur et al.2015	Libya	Type 2 Diabetes Mellitus	523	58.9		54.4	Hospital	Cross-Sectional	MMAS-8	63.9
llness perception, medication adherence and glycemic control among primary health-care patients with type 2 diabetes mellitus at Port Said City, Egypt (72)	Saudi et al.2022	Egypt	Type 2 Diabetes Mellitus	265	58.5		58.8	Hospital	Cross-Sectional	MMAS-8	32.5
Low medication adherence and its associated factors among patients with type 2 diabetes mellitus attending Amana Hospital in Dar es Salaam, Tanzania: a cross-sectional study (95)	Doyaa et al.2024	Tanzania	Type 2 Diabetes Mellitus	248	70.2		59.8	Hospital	Cross-Sectional	MMAS-8	26.2
Medication Adherence and Contributing Factors Among Type 2 Diabetes Patients at Adama Hospital Medical College in Eastern Ethiopia (34)	Yosef et al.2023	Ethiopia	Type 2 Diabetes Mellitus	245	44.9		48.6	Hospital	Cross-Sectional	MARS-5	29.4
Medication adherence and factors associated with poor adherence among type 2 diabetes mellitus patients on follow-up at Kenyatta National Hospital, Kenya (78)	Waari et al.2018	Kenya	Type 2 Diabetes Mellitus	290	67.6		56.6	Hospital	Cross-Sectional	MMAS-8	45.5
Medication adherence and its impact on glycemic control in type 2 diabetes mellitus patients with comorbidity: A multicenter cross-sectional study in Northwest Ethiopia (37)	Sendekie et al.2022	Ethiopia	Type 2 Diabetes Mellitus	403	45.2		45.2	Hospital	Cross-Sectional	GMAS	23.1
Medication adherence and self-care behaviours among patients with type 2 diabetes mellitus in Ghana (81)	Afaya et al.2020	Ghana	Type 2 Diabetes Mellitus	330	68.2		57.5	Hospital	Cross-Sectional	MAQ	84.5
Medication Adherence in Type 2 Diabetes Patients in Nigeria (59)	Jackson et al.2015	Nigeria	Type 2 Diabetes Mellitus	303	56.5			Hospital	Cross-Sectional	MMAS-8	19.8
Medication adherence in type 2 diabetes patients: Study of patients in alimosho general hospital, Igando, Lagos, Nigeria (60)	Olufunsho and Osuolale.2015	Nigeria	Type 2 Diabetes Mellitus	152	72.4			Hospital	Descriptive (Retrospective & Prospective)	MARS	86.8
Non-adherence to medication and associated factors among type 2 diabetes patients	Murwanashyaka	Rwanda	Type 2 Diabetes Mellitus	200	53.0			Hospital	Cross-Sectional	MMAS-4	46.5

at Clinique Medicale Fraternite, Rwanda: a cross-sectional study (96)	et al.2022									
The Association between Diabetes-Related Distress and Medication Adherence in Adult Patients with Type 2 Diabetes Mellitus: A Cross-Sectional Study (82)	Kretchy et al.2020	Ghana	Type 2 Diabetes Mellitus	188	72.3	59.3	Hospital	Cross-Sectional	MARS	34.5
The impact of pharmacist-led medication therapy management on medication adherence in patients with type 2 diabetes mellitus: a randomized controlled study (44)	ERKU et al.2017	Ethiopia	Type 2 Diabetes Mellitus	107	43.0	60.6	Hospital	Randomized Controlled	MMAS-8	9.2
The Influence of National Health Insurance on Medication Adherence Among Outpatient Type 2 Diabetics in Southwest Nigeria (67)	Ajibola et al.2018	Nigeria	Type 2 Diabetes Mellitus	110	50.9		Hospital	Cross-Sectional	MMAS-8	10.9
Validation and psychometric properties of the 8-item Morisky Medication Adherence Scale (MMAS-8) in Type 2 diabetes patients in sub-Saharan Africa (98)	Tandon et al.2015	Togo	Type 2 Diabetes Mellitus	308	72.7	57.5	Hospital	Cross-Sectional	MMAS-8	18.8

MMAS-8 (Morisky Medication Adherence Scale – 8 items), SMAQ (Simplified Medication Adherence Questionnaire), MARS (Medication Adherence Report Scale), MCQ (Medication Compliance Questionnaire), PMAQ (Patient Medication Adherence Questionnaire), CQR (Compliance Questionnaire on Rheumatology), GMAS (General Medication Adherence Scale), MGLS (Morisky-Green-Levine Scale), MAQ (Medication Adherence Questionnaire), MAS (Medication Adherence Scale), MKQ (Medication Knowledge Questionnaire), BMQ (Brief medication Questionnaire).

For peer review only

Discussion

In this scoping review, we included 88 studies that revealed the use of a diversity of tools to assess treatment adherence in chronic conditions across African countries. MMAS-8 was the most commonly used, followed by MARS and other scales such as SMAQ, MCQ, PMAQ and CQR, used in specific contexts. The widespread use of the MMAS-8 adherence scale was consistent with findings from studies conducted in various African contexts, as well as international studies, which highlight its simplicity, practicality, reliability, and applicability across multiple clinical conditions (106, 107).

In most studies, the absence of validation of adherence tools within local African contexts was identified (107). Many scales were applied directly or merely translated, without proper linguistic, sociocultural adaptation or local psychometric validation, disregarding the linguistic diversity and cultural specificities of the continent (98, 108). For example, one study conducted in Ethiopia used the MMAS-8 translated into the local language without formal psychometric validation (106). This practice may introduce interpretation bias, particularly among populations with low health literacy, making it difficult to understand terms such as “adherence” or “regular use.” Although adherence scales that are translated, culturally adapted, and locally validated can be feasible and reliable, there was a notable prevalence of imported tools used without local validation. In Morocco and Sudan, for instance, the GMAS was validated; however, other scales still lack regional and local adaptations (108, 109).

Despite the widespread use of the MMAS-8 adherence scale, only three studies conducted in Africa (Togo, Nigeria, and Uganda) have reported formal psychometric and cultural validation of this adherence tool (12, 98, 110). Formal validation is essential to ensure that the instrument truly measures the construct of adherence within the diverse African contexts, which are characterised by linguistic diversity, health literacy barriers, and cultural influence (99, 106, 109, 111). In most studies, the MMAS-8 was only directly translated, without consistent psychometric validation, raising concerns about the reliability of the information produced and its applicability in monitoring multiple clinical conditions in African settings. Studies conducted in Ethiopia and Nigeria reported semantic comprehension difficulties with the MMAS-8, requiring modifications to the scale's content

For peer review only

to ensure clarity without compromising the essence of the original instrument (98, 110, 112, 113). This diversity reflects the lack of standardisation in adherence assessment and highlights the need for careful consideration when comparing results across studies.

Most of the adherence scales identified were developed outside the African continent, with cultural and linguistic validations still insufficiently established in the populations where they were applied. This raises concerns regarding the cultural sensitivity and contextual appropriateness of these scales to accurately measure treatment adherence across diverse African contexts.

Adherence rates showed high variability among the 88 studies included in this scoping review, ranging from 4% to 96.8%, depending on the country, the health condition, and the instrument used. This wide discrepancy may reflect both methodological challenges such as the use of non-standardised instruments and contextual factors, such as the type of disease, the age group of participants, and the characteristics of the health system in place (100). Chronic diseases such as hypertension and diabetes generally showed lower adherence rates, which is consistent with international studies (98, 99). On the other hand, conditions such as infection with the HIV demonstrated relatively higher adherence rates in some contexts, possibly due to greater investment in adherence programs in countries like Kenya and South Africa (114, 115).

Most studies on adherence scales included in this scoping review focus on the regions of West and East Africa, with emphasis on Ethiopia, Nigeria, Egypt, Kenya, and Cameroon, while countries in the North and South of the continent, such as Libya, Tunisia, Angola, Mozambique, and South Africa, were underrepresented or not represented at all. This may indicate a disparity in the geographical distribution of scientific production related to adherence scales across different African countries, with some countries having higher research output than others possibly due to more developed research infrastructures (108). This unequal distribution may create gaps in regional understanding of the barriers and facilitators to adherence, compromising the development of public policies tailored to each context (116).

The predominance of studies conducted in hospital settings, rather than in community or rural environments, may limit the generalizability of the results, especially on a continent

For peer review only

where most of the population lives outside urban centres. This type of study requires clinical infrastructure to ensure strict protocol control, which favours research conducted in hospital environments (117).

The most studied health conditions were type 2 diabetes, hypertension, HIV, and epilepsy. The predominance of non-communicable chronic diseases is likely due to their increasing burden in the African continent (5). Adherence to treatment for these conditions was generally low, reflecting structural and behavioural challenges related to continuous medication use and disease understanding, especially in rural areas and populations with low health literacy (118, 119).

This study has some limitations. Although a systematic search strategy was conducted across various databases for this scoping review, it is possible that some relevant studies were not identified, especially those indexed in local databases and published in languages other than English. The heterogeneity among the studies in terms of clinical conditions and populations assessed, application contexts, and languages used may limit comparisons between scales and the generalisation of results to different African settings

Conclusion

In conclusion, this review has identified that important challenges remain in terms of tools to assess adherence in Africa due to the lack of standardised, validated, and culturally adapted tools. The predominant use of the MMAS-8 adherence scale, without formal psychometric validation, raises concerns about the reliability and applicability of the data obtained across different linguistic, cultural, and social contexts on the continent.

Overall, this points to an urgent need for local validation of instruments that consider the linguistic and cultural diversity of Africa. Such tools should be integrated into national and regional health strategies, with guidelines to direct adaptation and psychometric validation, in order to strengthen systems for monitoring treatment adherence, improve the guidance of therapeutic interventions, and promote more accurate and equitable public policies tailored to the diverse contexts across the continent.

For peer review only

Acknowledgements

Acknowledgments are extended to the Faculties of Medicine and Pharmacy of the University of Porto for the scientific support that made this scoping review possible.

Special thanks are given to the Universidade São Tomás de Moçambique for the moral support during the continuation of the doctoral studies.

Author contribuitions

Conceptualization: Izaidino Muchanga, Luís Midão, Elísio Costa and Bernardo Sousa-Pinto

Data curation: Izaidino Muchanga, Luís Midão, Avelino Mazuze, Daniela Melo, Leovaldo Alcântara, and Teodora Figueiredo.

Writing-original draft: Izaidino Muchanga and Luís Midão

Supervision: Elísio Costa and Bernardo Sousa-Pinto

Writing-review & editing: Izaidino Muchanga, Luís Midão, Avelino Mazuze, Daniela Melo, Leovaldo Alcântara, Teodora Figueiredo, Elísio Costa and Bernardo Sousa-Pinto

Funding

This work was supported by the Fundação para a Ciência e a Tecnologia (FCT), Portugal, through a PhD scholarship granted to Izaidino Jaime Muchanga (PRT/BD/154774/2022-https://doi.org/10.54499/PRT/BD/154774/2022), funded by national funds, within the scope of the PhD program in Clinical and Health Services Research at the Faculty of Medicine of the University of Porto.

Competing Interests

The authors declare that they have no competing interests.

For peer review only

References

1. Al-Moamary M, Aggarwal B, Al-Ahmad M, Sriprasart T, Koenig S, Levy G, et al. Are Treatment Adherence Factors Apparent in Patients with Asthma and to Physicians? Results from the APPaRENT 3 Survey. Adv Ther. 2025;42(3):1506–21.
2. Ho PM, Bryson CL, Rumsfeld JS. Medication adherence: its importance in cardiovascular outcomes. Circulation. 2009;119(23):3028–35.
3. Lam WY, Fresco P. Medication Adherence Measures: An Overview. Biomed Res Int. 2015;2015:217047.
4. Hardon A, Davey, S., Gerrits, T., Hodgkin, C., Irunde, H., Kgatlwane, J., Kinsman, J., Nakiyemba, A., & Laing, R. From access to adherence: The challenges of antiretroviral treatment: Studies from Botswana, Tanzania and Uganda. World Health Organization. Geneva2006. 320 p.
5. Gouda HN, Charlson F, Sorsdahl K, Ahmadzada S, Ferrari AJ, Erskine H, et al. Burden of non-communicable diseases in sub-Saharan Africa, 1990-2017: results from the Global Burden of Disease Study 2017. Lancet Glob Health. 2019;7(10):e1375–e87.
6. Nachega JB, Uthman OA, Peltzer K, Richardson LA, Mills EJ, Amekudzi K, et al. Association between antiretroviral therapy adherence and employment status: systematic review and meta-analysis. Bull World Health Organ. 2015;93(1):29–41.
7. Hardon AP, Akurut D, Comoro C, Ekezie C, Irunde HF, Gerrits T, et al. Hunger, waiting time and transport costs: time to confront challenges to ART adherence in Africa. AIDS Care. 2007;19(5):658–65.
8. Almeida P, Rafael, C., Pimentel, V., Abecasis, A., Sebastião, C., & Morais, J. Adherence to Antiretroviral Therapy(ART) Among HIV-1 Patients From Sub-Saharan Africa: A Systematic Review. Preprintsorg (wwwpreprintsorg). 2014;2.
9. Ware NC, Idoko J, Kaaya S, Biraro IA, Wyatt MA, Agbaji O, et al. Explaining adherence success in sub-Saharan Africa: an ethnographic study. PLoS Med. 2009;6(1):e11.
10. Sankar UV, Lipska K, Mini GK, Sarma PS, Thankappan KR. The adherence to medications in diabetic patients in rural Kerala, India. Asia Pac J Public Health. 2015;27(2):NP513–23.
11. Morisky DEL, W.G. and Levine,D.M. Concurrent and predictive validity of a self-reported measure of medication adherence. Medical care. 1986;24:8.
12. Afolabi MO, Daropale VO, Irinoye AI, Adegoke AA. Health-seeking behaviour and student perception of health care services in a university community in Nigeria. Health. 2013;05(05):817–24.
13. Page MJ, McKenzie JE, Bossuyt PM, Boutron I, Hoffmann TC, Mulrow CD, et al. The PRISMA 2020 statement: an updated guideline for reporting systematic reviews. BMJ. 2021;372:n71.
14. Arksey H, O'Malley L. Scoping studies: towards a methodological framework. International Journal of Social Research Methodology. 2005;8(1):19–32.
15. O'Brien LDHCKK. Scoping studies: advancing the methodology. Implementation Science. 2010.

For peer review only

16. Ouzzani M, Hammady H, Fedorowicz Z, Elmagarmid A. Rayyan-a web and mobile app for systematic reviews. Syst Rev. 2016;5(1):210.
17. D GT, Berhane Y, Worku A. Adherence to Antihypertensive Treatment and Associated Factors in Central Ethiopia. Int J Hypertens. 2020;2020:9540810.
18. Jemal A, Abdela J. Adherence to Oral Antidiabetic Medications among Type 2 Diabetic (T2DM) Patients in Chronic Ambulatory Wards of Hiwot Fana Specialized University Hospital, Harar, Eastern Ethiopia: A Cross Sectional Study. Journal of Diabetes & Metabolism. 2017;08(01).
19. Hasiso TY, Desse TA. Adherence to Treatment and Factors Affecting Adherence of Epileptic Patients at Yirgalem General Hospital, Southern Ethiopia: A Prospective Cross-Sectional Study. PLoS One. 2016;11(9):e0163040.
20. Shumet S, Wondie M, Ayano G, Asfaw H, Kassew T, Mesafint G. Antiepileptic Drug Adherence and Its Associated Factors among Epilepsy Patients on Follow-ups at Amanuel Mental Specialized Hospital, Ethiopia. Ethiop J Health Sci. 2022;32(5):913–22.
21. Dima SA, Shibeshi MS. Antiepileptic drug adherence in children in southern Ethiopia: A cross sectional study. PLoS One. 2022;17(2):e0263821.
22. Getnet A, Woldeyohannes SM, Bekana L, Mekonen T, Fekadu W, Menberu M, et al. Antiepileptic Drug Nonadherence and Its Predictors among People with Epilepsy. Behav Neurol. 2016;2016:3189108.
23. Asgedom SW, Atey TM, Desse TA. Antihypertensive medication adherence and associated factors among adult hypertensive patients at Jimma University Specialized Hospital, southwest Ethiopia. BMC Res Notes. 2018;11(1):27.
24. Tareke M, Tesfaye S, Amare D, Belete T, Abate A. Antipsychotic medication non-adherence among schizophrenia patients in Central Ethiopia. S Afr J Psychiatr. 2018;24:1124.
25. Mohammed F, Geda B, Yadeta TA, Dessie Y. Antipsychotic medication non-adherence and factors associated among patients with schizophrenia in eastern Ethiopia. BMC Psychiatry. 2024;24(1):108.
26. Kassahun G, Moges G, Demessie Y. Assessment of Patients' Adherence to Antiepileptic Medications at Dessie Referral Hospital, Chronic Follow-Up, South Wollo, Amhara Region, North East Ethiopia. Neurol Res Int. 2018;2018:5109615.
27. Abebe K, Deresse B, Negeri KG. Non adherence to treatment and the associated factors in patients with epilepsy in Southern Ethiopia. Healthcare in Low-resource Settings. 2022;10(1).
28. Getachew S, Addissie A, Seife E, Wakuma T, Unverzagt S, Jemal A, et al. Breast Nurse Intervention to Improve Adherence to Endocrine Therapy Among Breast Cancer Patients in South Ethiopia. Oncologist. 2022;27(8):e650–e60.
29. Demoz GT, Berha AB, Alebachew Woldu M, Yifter H, Shibeshi W, Engidawork E. Drug therapy problems, medication adherence and treatment satisfaction among diabetic patients on follow-up care at Tikur Anbessa Specialized Hospital, Addis Ababa, Ethiopia. PLoS One. 2019;14(10):e0222985.
30. Berhe DF, Taxis K, Haaijer-Ruskamp FM, Mulugeta A, Mengistu YT, Burgerhof JGM, et al. Impact of adverse drug events and treatment satisfaction on patient adherence with antihypertensive medication - a study in ambulatory patients. Br J Clin Pharmacol. 2017;83(9):2107–17.

For peer review only

31. Shimels T, Asrat Kassu R, Bogale G, Bekele M, Getnet M, Getachew A, et al. Magnitude and associated factors of poor medication adherence among diabetic and hypertensive patients visiting public health facilities in Ethiopia during the COVID-19 pandemic. PLoS One. 2021;16(4):e0249222.
32. Alemayehu Y, Asfaw H, Girma M. Magnitude and factors associated with treatment non-adherence among patients with depressive disorders at St. Amanuel Mental Specialized Hospital, Addis Ababa, Ethiopia, 2019: A cross sectional study. PLoS One. 2022;17(7):e0271713.
33. Gudeta DB, Leta K, Alemu B, Kandula UR. Medication adherence and associated factors among psychiatry patients at Asella Referral and Teaching Hospital in Oromia, Ethiopia: Institution based cross sectional study. PLoS One. 2023;18(4):e0283829.
34. Yosef T, Nureye D, Tekalign E, Assefa E, Shifera N. Medication Adherence and Contributing Factors Among Type 2 Diabetes Patients at Adama Hospital Medical College in Eastern Ethiopia. SAGE Open Nurs. 2023;9:23779608231158975.
35. Ali M, Alemu T, Sada O. Medication adherence and its associated factors among diabetic patients at Zewditu Memorial Hospital, Addis Ababa, Ethiopia. BMC Res Notes. 2017;10(1):676.
36. Teshome DF, Bekele KB, Habitu YA, Gelagay AA. Medication adherence and its associated factors among hypertensive patients attending the Debre Tabor General Hospital, northwest Ethiopia. Integr Blood Press Control. 2017;10:1–7.
37. Sendekie AK, Netere AK, Kasahun AE, Belachew EA. Medication adherence and its impact on glycemic control in type 2 diabetes mellitus patients with comorbidity: A multicenter cross-sectional study in Northwest Ethiopia. PLoS One. 2022;17(9):e0274971.
38. Abate TW. Medication non-adherence and associated factors among diabetes patients in Felege Hiwot Referral Hospital, Bahir Dar city administration, Northwest Ethiopia. BMC Res Notes. 2019;12(1):175.
39. Derejie MN, Dereje EN, Alemu DM, Tesfay YG, Hunduma F, Temie NM. Medication non-adherence and its associated factors among kidney transplant patients in a large teaching hospital in Ethiopia. BMC Nephrol. 2024;25(1):187.
40. Seid MA, Toleha HN, Sema FD. Medication Nonadherence and Associated Factors among Heart Failure Patients at University of Gondar Comprehensive Specialized Hospital, Northwest Ethiopia. Int J Chronic Dis. 2023;2023:1824987.
41. Belachew EA, Netere AK, Sendekie AK. Medication regimen complexity and its impact on medication adherence and asthma control among patients with asthma in Ethiopian referral hospitals. Asthma Res Pract. 2022;8(1):7.
42. Desalegn D, Girma S, Abdeta T. Quality of life and its association with current substance use, medication non-adherence and clinical factors of people with schizophrenia in Southwest Ethiopia: a hospital-based cross-sectional study. Health Qual Life Outcomes. 2020;18(1):82.
43. Abegaz TM, Sori LM, Toleha HN. Self-Reported Adverse Drug Reactions, Medication Adherence, and Clinical Outcomes among Major Depressive Disorder Patients in Ethiopia: A Prospective Hospital Based Study. Psychiatry J. 2017;2017:5812817.
44. Erku DA, Ayele AA, Mekuria AB, Belachew SA, Hailemeskel B, Tegegn HG. The impact of pharmacist-led medication therapy management on medication adherence

For peer review only

in patients with type 2 diabetes mellitus: a randomized controlled study. Pharm Pract (Granada). 2017;15(3):1026.

45. Wedajo S, Degu G, Deribew A, Ambaw F. The role of health facility and individual level characteristics on medication adherence among PLHIV on second-line antiretroviral therapy in Northeast Ethiopia: use of multi-level model. AIDS Res Ther. 2022;19(1):17.

46. Birhanie SA, Getie GA, Tesfa M, Mulugeta H, Gedfew M, Mekete YS, et al. Treatment adherence and associated factors among glaucoma patients attending Ophthalmic units of referral hospitals in North West Ethiopia, 2019. Front Ophthalmol (Lausanne). 2022;2:985893.

47. Okwuonu Chimezie G UNV, Chimezie Oluchi Jb , Ogah Okechukwu S , Mbanaso Augustus U , Odigwe Clement O. ADHERENCE TO ANTIHYPERTENSIVE MEDICATION AND ITS CORRELATES AMONG INDIVIDUALS WITH HYPERTENSION IN A SEMI-URBAN COMMUNITY

OF SOUTHERN NIGERIA. Journal of Biomedical Sciences. 2015;14:12.

48. Abah IO, Ojeh VB, Musa J, Ugoagwu P, Agaba PA, Agbaji O, et al. Clinical Utility of Pharmacy-Based Adherence Measurement in Predicting Virologic Outcomes in an Adult HIV-Infected Cohort in Jos, North Central Nigeria. J Int Assoc Provid AIDS Care. 2016;15(1):77–83.

49. Okunrinboye HI, Otakpor AN, Ilesanmi OS. Depression and medication-adherence in patients with hypertension attending a tertiary health facility in South-West Nigeria. Pan Afr Med J. 2019;33:27.

50. Bello SI, Ojieabu WA, Yusuf H. Drug utilization, medication adherence and treatment satisfaction amongst glaucoma patients receiving care at secondary health facilities. Journal of Pharmaceutical Health Services Research. 2023;14(1):71–8.

51. Okoro RN, Ummate I, Ohieku JD, Yakubu S, Adibe MO, Okonta MJ. Evaluation of medication adherence and predictors of sub-optimal adherence among pre-dialysis patients with chronic kidney disease. Med Access Point Care. 2020;4:2399202620954089.

52. Yusuf H, Magaji MG, Maiha BB, Yakubu SI, Haruna WC, Mohammed S. Impact of pharmacist intervention on antidepressant medication adherence and disease severity in patients with major depressive disorder in fragile north-east Nigeria. Journal of Pharmaceutical Health Services Research. 2021;12(3):410–6.

53. Ubaka CM, Ibe OG, Amorha KC, Isah A, Okonta MJ. Medication adherence among Nigerian patients with rheumatoid arthritis: a two instruments survey. Journal of Pharmaceutical Health Services Research. 2021;12(1):11–7.

54. Ogunnubi OP, Olagunju AT, Aina OF, Okubadejo NU. Medication Adherence Among Nigerians with Schizophrenia: Correlation Between Clinico-Demographic Factors and Quality of Life. Ment Illn. 2017;9(1):6889.

55. Fadare J, Olamoyegun M, Gbadegesin BA. Medication adherence and direct treatment cost among diabetes patients attending a tertiary healthcare facility in Ogbomosho, Nigeria. Malawi Med J. 2015;27(2):65–70.

56. Oluwole EO, Osibogun O, Adegoke O, Adejimi AA, Adewole AM, Osibogun A. Medication adherence and patient satisfaction among hypertensive patients attending

outpatient clinic in Lagos University Teaching Hospital, Nigeria. Niger Postgrad Med J. 2019;26(2):129–37.
57. Olaniran GO, Akodu BA, Olaniran AA, Bamidele JO, Ogunyemi AO, Idowu OO. Medication Adherence and Perceived Family Support Among Elderly Patients with Hypertension Attending a Specialty Clinic in Lagos, Nigeria. Annals of Health Research. 2023;9(1):30–42.
58. Aduloju OP, Aduloju, T., Ade-Ojo, I. P., & Akintayo, A. A. Medication adherence in HIV-positive pregnant women on antiretroviral therapy attending antenatal clinics in Ado metropolis, south-west Nigeria: A multicentre study. S Afr J Obstet Gynaecol. 2020;26.
59. Jackson IL, Adibe MO, Okonta MJ, Ukwe CV. Medication adherence in type 2 diabetes patients in Nigeria. Diabetes Technol Ther. 2015;17(6):398–404.
60. Awodele O, Osuolale JA. Medication adherence in type 2 diabetes patients: study of patients in Alimosho General Hospital, Igando, Lagos, Nigeria. Afr Health Sci. 2015;15(2):513–22.
61. Olorunfemi O, Ojewole F. Medication belief as correlate of medication adherence among patients with diabetes in Edo State, Nigeria. Nurs Open. 2019;6(1):197–202.
62. Okpataku CI, Kwanashie HO, Ejiofor JI, Olisah VO. Medication compliance behavior in psychiatric out-patients with psychoactive substance use comorbidity in a Nigerian tertiary hospital. Niger J Clin Pract. 2015;18(3):371–6.
63. Seb-Akahomen O, Lawani A, James B. Mood and anxiety disorders adversely influence medication adherence to antiretroviral therapy among people living with HIV/AIDS in Nigeria. HIV & AIDS Review. 2018;17(2):91–7.
64. Heestermans T, Browne JL, Aitken SC, Vervoort SC, Klipstein-Grobusch K. Determinants of adherence to antiretroviral therapy among HIV-positive adults in sub-Saharan Africa: a systematic review. BMJ Glob Health. 2016;1(4):e000125.
65. Ibrahim AW, Yahya S, Pindar SK, Wakil MA, Garkuwa A, Sale S. Prevalence and predictors of sub-optimal medication adherence among patients with severe mental illnesses in a tertiary psychiatric facility in Maiduguri, North-eastern Nigeria. Pan Afr Med J. 2015;21:39.
66. Ipinnimo TM, Abodunrin OL, Durowade KA. Relationship between Cost of Care and Medication Adherence among Hypertensive Patients Attending a Tertiary Health Facility in Ekiti State, Nigeria. Journal of Community Medicine and Primary Health Care. 2022;34(1):38–57.
67. Ajibola SS, Timothy FO. The Influence of National Health Insurance on Medication Adherence Among Outpatient Type 2 Diabetics in Southwest Nigeria. J Patient Exp. 2018;5(2):114–9.
68. Adisa R, Ilesanmi OA, Fakeye TO. Treatment adherence and blood pressure outcome among hypertensive out-patients in two tertiary hospitals in Sokoto, Northwestern Nigeria. BMC Cardiovasc Disord. 2018;18(1):194.
69. Akpor OA, Olowolaju FO. Treatment satisfaction and medication adherence among hypertensive patients in a teaching hospital in Ekiti State, Nigeria. Journal of Integrative Nursing. 2022;4(2):59–67.

For peer review only

70. Okasha TA, Radwan DN, Elkholy H, Hendawy H, Shourab E, Teama RRA, et al. Psycho-demographic and clinical predictors of medication adherence in patients with bipolar I disorder in a university hospital in Egypt. S Afr J Psychiatr. 2020;26:1437.
71. Salama HM, Saudi RA. Effect of patients beliefs about medications on adherence to drugs in diabetic patients attending family medicine outpatient clinic in Ismailia, Egypt. J Diabetes Metab Disord. 2020;19(2):951–8.
72. Saudi RA, Abbas RA, Nour-Eldein H, Sayed Ahmed HA. Illness perception, medication adherence and glycemic control among primary health-care patients with type 2 diabetes mellitus at Port Said City, Egypt. Diabetol Int. 2022;13(3):522–30.
73. Sayed Ahmed HA, Abdelsalam NE, Joudeh AI, Abdelrahman AG, Eldahshan NA. Association of treatment satisfaction and physician trust with glycemic control among primary care patients with type 2 diabetes in Egypt. Diabetol Int. 2024;15(1):67–75.
74. Zayed HS, Medhat BM, Seif EM. Evaluation of treatment adherence in patients with Behcet's disease: its relation to disease manifestations, patients' beliefs about medications, and quality of life. Clin Rheumatol. 2019;38(3):761–8.
75. Kioko MT, Pertet AM. Factors contributing to antiretroviral drug adherence among adults living with HIV or AIDS in a Kenyan rural community. Afr J Prim Health Care Fam Med. 2017;9(1):e1–e7.
76. Xiong S, Peoples N, Ostbye T, Olsen M, Zhong X, Wainaina C, et al. Family support and medication adherence among residents with hypertension in informal settlements of Nairobi, Kenya: a mixed-method study. J Hum Hypertens. 2023;37(1):74–9.
77. Vreeman RC, Nyandiko WM, Liu H, Tu W, Scanlon ML, Slaven JE, et al. Measuring adherence to antiretroviral therapy in children and adolescents in western Kenya. J Int AIDS Soc. 2014;17(1):19227.
78. Waari G, Mutai J, Gikunju J. Medication adherence and factors associated with poor adherence among type 2 diabetes mellitus patients on follow-up at Kenyatta National Hospital, Kenya. Pan Afr Med J. 2018;29:82.
79. Abu EK, Antiri EO, Ocansey S, Ntodie M, Abokyi S, Abraham CH. Associations between personality traits and adherence to treatment in patients with primary open-angle glaucoma in an African population. Clin Exp Optom. 2023;106(5):509–15.
80. Adu C, Mensah KA, Ahinkorah BO, Osei D, Tetteh AW, Seidu AA. Socio-demographic factors associated with medication adherence among People Living with HIV in the Kumasi Metropolis, Ghana. AIDS Res Ther. 2022;19(1):50.
81. Afaya RA, Bam V, Azongo TB, Afaya A, Kusi-Amponsah A, Ajusiyine JM, et al. Medication adherence and self-care behaviours among patients with type 2 diabetes mellitus in Ghana. PLoS One. 2020;15(8):e0237710.
82. Kretchy IA, Koduah A, Ohene-Agyei T, Boima V, Appiah B. The Association between Diabetes-Related Distress and Medication Adherence in Adult Patients with Type 2 Diabetes Mellitus: A Cross-Sectional Study. J Diabetes Res. 2020;2020:4760624.
83. Awad A, Osman N, Altayib S. Medication adherence among cardiac patients in Khartoum State, Sudan: a cross-sectional study. Cardiovasc J Afr. 2017;28(6):350–5.
84. Elsayed MA, El-Sayed NM, Badi S, Ahmed MH. Factors affecting adherence to antiepileptic medications among Sudanese individuals with epilepsy: A cross-sectional survey. J Family Med Prim Care. 2019;8(7):2312–7.

For peer review only

85. Ali ZAH, Abd El-Raheem GOH, Noma M. Status of rheumatoid arthritis practice and treatment in Sudan. Scientific African. 2023;22.
86. Ngahane BH, Pefura-Yone EW, Mama M, Tengang B, Nganda MM, Wandji A, et al. Evaluation of factors affecting adherence to asthma controller therapy in chest clinics in a sub-Saharan African setting: a cross-sectional study. Afr Health Sci. 2016;16(1):194–200.
87. Aminde LN, Tindong M, Ngwasiri CA, Aminde JA, Njim T, Fondong AA, et al. Adherence to antidiabetic medication and factors associated with non-adherence among patients with type-2 diabetes mellitus in two regional hospitals in Cameroon. BMC Endocr Disord. 2019;19(1):35.
88. Egenasi C, Steinberg WJ, Raubenheimer JE. Beliefs about medication, medication adherence and seizure control among adult epilepsy patients in Kimberley, South Africa. South African Family Practice. 2015;57(5):326–32.
89. Hirasen K, Evans D, Jinga N, Grabe R, Turner J, Mashamaite S, et al. Using a Self-Administered Electronic Adherence Questionnaire to Identify Poor Adherence Amongst Adolescents and Young Adults on First-Line Antiretroviral Therapy in Johannesburg, South Africa. Patient Prefer Adherence. 2020;14:133–51.
90. Achouri MY, Mammeri M, Sehanine Y, Selka MA, Ghomari WI, Lahmer A, et al. [Factors associated with medication non-adherence in patients with type 2 diabetes mellitus: The first Algerian survey]. Ann Pharm Fr. 2019;77(6):506–15.
91. Kara S. General self-efficacy and hypertension treatment adherence in Algerian private clinical settings. J Public Health Afr. 2022;13(3):2121.
92. Musumari PM, Wouters E, Kayembe PK, Kiumbu Nzita M, Mbikayi SM, Suguimoto SP, et al. Food insecurity is associated with increased risk of non-adherence to antiretroviral therapy among HIV-infected adults in the Democratic Republic of Congo: a cross-sectional study. PLoS One. 2014;9(1):e85327.
93. Olorunfemi O. Health literacy, medication belief, and demographic factors as correlates of medication adherence in person with diabetes mellitus. Journal of Diabetology. 2023;14(2):100–6.
94. Ashur ST, Shah SA, Bosseri S, Morisky DE, Shamsuddin K. Illness perceptions of Libyans with T2DM and their influence on medication adherence: a study in a diabetes center in Tripoli. Libyan J Med. 2015;10(1):29797.
95. Doya IF, Yahaya JJ, Ngaiza AI, Bintabara D. Low medication adherence and its associated factors among patients with type 2 diabetes mellitus attending Amana Hospital in Dar es Salaam, Tanzania: a cross-sectional study. Int Health. 2024;16(2):200–7.
96. Murwanashyaka JD, Ndagijimana A, Biracyaza E, Sunday FX, Umugwaneza M. Non-adherence to medication and associated factors among type 2 diabetes patients at Clinique Medicale Fraternite, Rwanda: a cross-sectional study. BMC Endocr Disord. 2022;22(1):219.
97. Mbengue MAS, Sarr SO, Diop A, Ndour CT, Ndiaye B, Mboup S. Prevalence and determinants of adherence to antiretroviral treatment among HIV patients on first-line regimen: a cross-sectional study in Dakar, Senegal. Pan Afr Med J. 2019;33:95.
98. Tandon S, Chew M, Eklu-Gadegbeku CK, Shermock KM, Morisky DE. Validation and psychometric properties of the 8-item Morisky Medication Adherence Scale

For peer review only

(MMAS-8) in Type 2 diabetes patients in sub-Saharan Africa. Diabetes Res Clin Pract. 2015;110(2):129–36.
99. Okello S, Nasasira B, Muiru AN, Muyingo A. Validity and Reliability of a Self-Reported Measure of Antihypertensive Medication Adherence in Uganda. PLoS One. 2016;11(7):e0158499.
100. Macquart de Terline D, Kane A, Kramoh KE, Ali Toure I, Mipinda JB, Diop IB, et al. Factors associated with poor adherence to medication among hypertensive patients in twelve low and middle income Sub-Saharan countries. PLoS One. 2019;14(7):e0219266.
101. Solomon Mekonnen Abebe* YBaAW. Barriers to diabetes medication adherence in North West Ethiopia. Springer Open Journal. 2014.
102. Mebrahtu G, M MM, Okoth Achila O, Seyoum Y, Adnoy ET, Ovberedjo M. Antihypertensive Medication Adherence and Associated Factors: A Cross-Sectional Analysis of Patients Attending a National Referral Hospital in Asmara, Eritrea. Patient Prefer Adherence. 2021;15:2619–32.
103. Denison JA, Koole O, Tsui S, Menten J, Torpey K, van Praag E, et al. Incomplete adherence among treatment-experienced adults on antiretroviral therapy in Tanzania, Uganda and Zambia. AIDS. 2015;29(3):361–71.
104. Tsige AW, Kotiso TA, Ayenew KD, Ayele SG. Pharmacist intervention to improve adherence to medication among heart failure patients at North East Ethiopia hospital. Pharmacol Res Perspect. 2024;12(3):e1199.
105. Ahmed EH, Abdel-Aziz HR, Ahmed S. Medication adherence and its influencing factors in community-dwelling older adults with chronic illnesses in a rural area. Nursing Practice Today. 2023.
106. Jose M, Rajmohan P, Sulfath TS, Varma RP, Mohan M, Jose NK, et al. Medication adherence scales in non-communicable diseases: A scoping review of design gaps, constructs and validation processes. PLoS One. 2025;20(5):e0321423.
107. Awwad O, AlMuhaissen S, Al-Nashwan A, AbuRuz S. Translation and validation of the Arabic version of the Morisky, Green and Levine (MGL) adherence scale. PLoS One. 2022;17(10):e0275778.
108. Maryem A, Younes I, Yassmine M, Morad G, Karima B, Amal K, et al. Translation, cultural adaptation and validation of the General Medication Adherence Scale (GMAS) in moroccan patients with type-2 diabetes. BMC Nurs. 2023;22(1):302.
109. Mahmoud MA, Islam MA, Ahmed M, Bashir R, Ibrahim R, Al-Nemiri S, et al. Validation of the Arabic Version of General Medication Adherence Scale (GMAS) in Sudanese Patients with Diabetes Mellitus. Risk Manag Healthc Policy. 2021;14:4235–41.
110. De Las Cuevas C, Penate W. Psychometric properties of the eight-item Morisky Medication Adherence Scale (MMAS-8) in a psychiatric outpatient setting. Int J Clin Health Psychol. 2015;15(2):121–9.
111. Merten S, Kenter E, McKenzie O, Musheke M, Ntalasha H, Martin-Hilber A. Patient-reported barriers and drivers of adherence to antiretrovirals in sub-Saharan Africa: a meta-ethnography. Trop Med Int Health. 2010;15 Suppl 1:16–33.
112. Cabral AC, Moura-Ramos M, Castel-Branco M, Fernandez-Llimos F, Figueiredo IV. Cross-cultural adaptation and validation of a European Portuguese version of the

For peer review only

8-item Morisky medication adherence scale. Rev Port Cardiol (Engl Ed). 2018;37(4):297–303.

113. Evangeli M, Foster C, Musiime V, Fidler S, Seeley J, Frize G, et al. Cultural Adaption, Translation, Preliminary Reliability and Validity of Key Psychological and Behavioural Measures for 18 to 25 Year-Olds Living with HIV in Uganda: A Multi-Stage Approach. AIDS Behav. 2024;28(3):924–35.

114. Vreeman RC, Scanlon ML, Tu W, Slaven JE, McAteer CI, Kerr SJ, et al. Validation of a self-report adherence measurement tool among a multinational cohort of children living with HIV in Kenya, South Africa and Thailand. J Int AIDS Soc. 2019;22(5):e25304.

115. Duong M, Piroth L, Grappin M, Forte F, Peytavin G, Buisson M, et al. Evaluation of the Patient Medication Adherence Questionnaire as a tool for self-reported adherence assessment in HIV-infected patients on antiretroviral regimens. HIV Clin Trials. 2001;2(2):128–35.

116. Lane J, Nakambale H, Kadakia A, Dambisya Y, Stergachis A, Odoch WD. A systematic scoping review of medicine availability and affordability in Africa. BMC Health Serv Res. 2024;24(1):91.

117. Adagba KO, Aliyu AA, Ejembi CL, Olorukooba AA, Joshua IA. Comparative Study of Patients' Adherence between Hospital-based and Communitybased Treatment for Multidrug Resistant Tuberculosis (MDR-TB) in Kaduna State. Journal of Community Medicine and Primary Health Care. 2023;35(1):112–24.

118. Kudzinesta M, Mubita M, Kalemeera F, Godman B, Hango E, Kibuule D. Utility of medicines information leaflets in hypertensive care in a setting with low health literacy: A cross-sectional study. Med Access Point Care. 2020;4:2399202620910031.

119. Chauke GD, Nakwafila O, Chibi B, Sartorius B, Mashamba-Thompson T. Factors influencing poor medication adherence amongst patients with chronic disease in low-and-middle-income countries: A systematic scoping review. Heliyon. 2022;8(6):e09716.

For peer review only

BMJ Open

Mapping Medication Adherence Tools in Africa: A Scoping Review

Journal:	*BMJ Open*
Manuscript ID	bmjopen-2025-112898.R1
Article Type:	Original research
Date Submitted by the Author:	16-Jul-2026
Complete List of Authors:	Muchanga, Izaidino; University of Porto, Department of Community Medicine; University of Porto, Center for Health Technologies and Services Research; São Tomás University of Mozambique<br>Midão, Luís ; University of Porto, RISE-Health, Competence Centre for Active and Healthy Ageing, Faculty of Pharmacy, University of Porto<br>Mazuze, Avelino ; University of Porto, Department of Community Medicine; University of Porto, Center for Health Technologies and Services Research; Lúrio University, Faculty of Health Sciences<br>Melo, Daniela ; University of Porto, RISE-Health, Competence Centre for Active and Healthy Ageing, Faculty of Pharmacy, University of Porto<br>Alcântara, Leovaldo ; University of Porto, RISE-Health, Competence Centre for Active and Healthy Ageing, Faculty of Pharmacy, University of Porto<br>Figueiredo, Teodora ; University of Porto, RISE-Health, Competence Centre for Active and Healthy Ageing, Faculty of Pharmacy, University of Porto<br>Costa, Elisio ; University of Porto, RISE-Health, Competence Centre for Active and Healthy Ageing, Faculty of Pharmacy, University of Porto<br>Sousa-Pinto, Bernardo; University of Porto, Department of Community Medicine, Information and Health Decision Sciences, Faculty of Medicine, University of Porto, Porto, Portugal ; University of Porto, Center for Health Technologies and Services Research, Health Research Network, University of Porto, Porto, Portugal
**Primary Subject Heading**:	Public health
Secondary Subject Heading:	Global health, Epidemiology, Health services research
Keywords:	Chronic Disease, Clinical Decision-Making, INFECTIOUS DISEASES

SCHOLARONE MANUSCRIPTS

I, the Submitting Author has the right to grant and does grant on behalf of all authors of the Work (as defined in the below author licence), an exclusive licence and/or a non-exclusive licence for contributions from authors who are: i) UK Crown employees; ii) where BMJ has agreed a CC-BY licence shall apply, and/or iii) in accordance with the terms applicable for US Federal Government officers or employees acting as part of their official duties; on a worldwide, perpetual, irrevocable, royalty-free basis to BMJ Publishing Group Ltd ("BMJ") its licensees and where the relevant Journal is co-owned by BMJ to the co-owners of the Journal, to publish the Work in this journal and any other BMJ products and to exploit all rights, as set out in our licence.

The Submitting Author accepts and understands that any supply made under these terms is made by BMJ to the Submitting Author unless you are acting as an employee on behalf of your employer or a postgraduate student of an affiliated institution which is paying any applicable article publishing charge ("APC") for Open Access articles. Where the Submitting Author wishes to make the Work available on an Open Access basis (and intends to pay the relevant APC), the terms of reuse of such Open Access shall be governed by a Creative Commons licence – details of these licences and which Creative Commons licence will apply to this Work are set out in our licence referred to above.

Other than as permitted in any relevant BMJ Author's Self Archiving Policies, I confirm this Work has not been accepted for publication elsewhere, is not being considered for publication elsewhere and does not duplicate material already published. I confirm all authors consent to publication of this Work and authorise the granting of this licence.

For peer review only

Mapping Medication Adherence Tools in Africa: A Scoping Review

Izaidino Jaime Muchanga[1,2,5], Luís Midão[3], Avelino Mazuze[1,2,4], Daniela Melo[3], Leovaldo Alcântara[3], Teodora Figueiredo[3], Elísio Costa[3], Bernardo Sousa-Pinto[1,2]

[1] Department of Community Medicine, Information and Health Decision Sciences, Faculty of Medicine, University of Porto, Porto, Portugal
[2] Center for Health Technologies and Services Research, Health Research Network, University of Porto, Porto, Portugal
[3] RISE-Health, Competence Centre for Active and Healthy Ageing, Faculty of Pharmacy, University of Porto, Porto, Portugal
[4]Faculty of Health Sciences, Lúrio University, Nampula, Mozambique
[5]São Tomás University of Mozambique, Xai-Xai, Mozambique

Correspondence email:izaidino@yahoo.com

For peer review only

Abstract

Objectives: To map medication adherence assessment tools used among African populations with chronic diseases and describe their methodological characteristics,validation status, cultural adaptation, administration methods, and reported adherence rates.
Design: Scoping review conducted in accordance with the Preferred Reporting Items for Systematic Reviews and Meta-Analyses extension for Scoping Reviews (PRISMA-ScR).
Data Sources: PubMed, Scopus, and Web of Science were searched on June 25, 2026.
Eligibility Criteria: Studies conducted in African countries that assessed medication adherence among individuals with chronic diseases using any adherence measurement tool were eligible. Studies focusing exclusively on acute conditions, non-medication adherence behaviours, reviews, editorials, protocols, and conference abstracts were excluded.
Data Extraction: Data were extracted using a standardised charting form and summarized descriptively. Information on study characteristics, adherence measurement tools, validation and cultural adaptation procedures, administration methods, disease-specific applicability, supplementary adherence assessment methods, and reported adherence rates was synthesised narratively.
Results: Ninety-eight studies conducted across 27 African countries were included. Ethiopia (n=36) and Nigeria (n=22) contributed the largest number of studies. Type 2 Diabetes (n=29), Hypertension (n=18), and HIV (n=13) were the most frequently investigated conditions. The 8-item Morisky Medication Adherence Scale (MMAS-8) was the most used instrument, appearing in 50 studies. Only 15 studies (15.3%) reported local validation and/or cultural adaptation of adherence instruments. Most studies relied on generic self-report measures administered by interviewers, while relatively few incorporated supplementary methods such as pharmacy refill records, pill counts, electronic monitoring, viral load assessment, or biological markers. Reported medication adherence rates varied substantially across diseases, ranging from 0% to 96.8%.
Conclusions: Medication adherence assessment in Africa is characterized by considerable methodological variability and limited evidence of local validation and cultural adaptation. The widespread use of generic self-report instruments and the scarcity of objective adherence measures highlight the need for greater standardisation and context-specific validation of adherence assessment tools across diverse African healthcare settings.

Keywords: Adherence scales, adherence to treatment, chronic diseases and Africa

For peer review only - http://bmjopen.bmj.com/site/about/guidelines.xhtml

Strengths and Limitations of this Study

- This scoping review followed the PRISMA-ScR guidelines and systematically mapped medication adherence assessment tools used across diverse African healthcare settings and chronic disease conditions.
- The review provides a comprehensive methodological overview of adherence measurement approaches, including validation status, cultural adaptation, administration mode, and the use of complementary adherence assessment methods.
- Studies from multiple African countries and a broad range of chronic diseases were included, enabling identification of methodological patterns and evidence gaps across the continent.
- As recommended for scoping reviews, no formal appraisal of methodological quality or risk of bias was conducted, limiting assessment of the robustness of individual studies.
- Restriction to studies published in English and French, together with the possibility that some studies indexed exclusively in regional databases were not captured, may have resulted in the omission of relevant evidence.

Introduction

Medication adherence generally refers to the extent to which patients take medications as prescribed and continue treatment according to healthcare recommendations [1, 2]. Adherence to prescribed treatment regimens is essential for achieving successful therapeutic outcomes, especially in chronic disease management, where it enhances chronic disease prognosis and reduces long-term healthcare expenditures [3, 4]. Non-adherence constitutes a major public health challenge associated with poorer clinical outcomes, reduced quality of life, increased morbidity and mortality, and substantial healthcare costs [5, 6]. Current evidence indicates that approximately 50% of patients receiving long-term treatment for chronic conditions do not adhere adequately to prescribed therapies, with lower adherence rates being frequently observed in low and middle-income countries, including Sub-Saharan Africa, where both communicable and non-communicable disease burdens remain high [3, 4, 7].

For peer review only - http://bmjopen.bmj.com/site/about/guidelines.xhtml

Appropriate tools are essential for measuring medication adherence accurately. Various methods have been described, including direct methods, such as measurement of drugs or metabolites in biological samples, and indirect methods, such as pill counts, pharmacy refill records, electronic monitoring systems, and self-reported questionnaires [5, 8]. The selection of an appropriate adherence assessment approach should consider the clinical context and characteristics of the target population, particularly in African settings where socioeconomic and cultural factors may significantly influence adherence behaviors and reporting accuracy [4, 9]. However, despite the importance of adherence assessment in African countries, there is limited availability of validated and culturally adapted scales. The most frequent adherence scales, such as the Morisky-Green scale (MGS), the Morisky Medication Adherence Scale (MMAS-4 and MMAS-8 (MMAS) the Medication Adherence Rating Scale (MARS), and the Medication Compliance Questionnaire (MCQ) [4, 8, 9], were developed and validated outside Africa, raising concerns about their direct applicability in African populations due to cultural, linguistic, and socioeconomic differences[10, 11].

Although previous studies have reported medication adherence levels across different African countries and disease conditions, there remains a substantial gap in the systematic synthesis of the performance, validity, and diversity of instruments used to assess medication adherence across the continent have not been comprehensive evaluated. In fact, to date, no effort has been made to map the available adherence assessment tools while simultaneously identifying operational and methodological challenges associated with their use in African populations. Such processes are essential steps toward identifying (i) the best tools to measure adherence in the African context, and (ii) gaps that need to be overcome by the development of standardized and contextually appropriate adherence assessment protocols.

Therefore, this scoping review we aimed to map and characterize medication adherence assessment tools used in African populations with chronic diseases, including their validation status, cultural adaptation processes, administration characteristics, and reported adherence outcomes.

For peer review only - http://bmjopen.bmj.com/site/about/guidelines.xhtml

Methods

This scoping review was conducted following the Preferred Reporting Items for Systematic Reviews and Meta-Analyses extension for Scoping Reviews (PRISMA-ScR) guidelines [12]. The completed PRISMA-ScR checklist is provided as Supplementary Material 1. The methodological approach was based on the framework proposed by Arksey and O'Malley[13], further refined by Levac et al [14], and included: identification of the research question, to explore and map the tools available for assessing medication adherence across Africa; identification of relevant studies through a systematic search strategy across multiple electronic databases and grey literature sources; selection of studies using predefined inclusion and exclusion criteria based on relevance to the topic and objectives; data extraction using a structured form to collect key characteristics, types of adherence tools, settings, populations, and implementation contexts; and synthesis of results through narrative and thematic approaches, highlighting patterns, gaps, and methodological diversity among the identified tools to ensure transparency and reproducibility.

A scoping review of literature was chosen because it allows for a comprehensive and detailed mapping of the various medication adherence tools used in Africa, a field that is still emerging and methodologically diverse. This approach facilitates the identification of knowledge gaps and conceptual differences, providing an essential foundation for future research and policies tailored to the complexity and heterogeneity of African contexts.

Research Question

This scoping review addressed the following research questions: (i)which medication adherence tools have been used in African populations with chronic diseases? (ii) what are the methodological characteristics of these tools, including validation status, cultural adaptation, administration mode, and disease-specific applicability? (iii) what medication adherence rates have been reported using these tools across African settings and chronic conditions? (iv) what methodological challenges and gaps exist in medication adherence assessment in Africa?

For peer review only - http://bmjopen.bmj.com/site/about/guidelines.xhtml

Eligibility Criteria

Studies were included if they involved populations of any age from African countries and described the use or validation of instruments to measure medication adherence. The review considered all healthcare settings and focused exclusively on peer-reviewed articles published in English and French. Due to feasibility constraints, studies published in other languages were not included. There were no limitations on the year of publication.

Grey literature, conference abstracts, opinion pieces, editorials, and other non-peer-reviewed sources were excluded.

Information Sources and Search Strategy

The literature search was carried out across three major databases: PubMed, Scopus, and Web of Science. The final search was completed on December 10, 2024, and updated in June 25, 2026. Search terms were selected based on MeSH terms and relevant keywords to ensure comprehensive coverage of the topic. The search strategy combined geographic keywords related to African countries with terms describing medication adherence (e.g., "medication compliance", "treatment nonadherence") and terms related to measurement instruments (e.g., "scale", "tool", "survey", "assessment"). Boolean operators like AND, OR, and NOT were used to combine and refine the search terms. No filters were applied regarding publication date or study type beyond the language and peer-review criteria. The complete search strategy is available in the supplementary material 2.

Study Selection Process

The screening process was carried out in three sequential stages: initial screening of titles, followed by abstracts, and subsequently full-text articles. Two reviewers independently assessed the eligibility of studies at each stage. Any discrepancies were resolved through consultation with a third reviewer to reach consensus. The screening and selection process was facilitated using Rayyan [15], a web-based tool designed to streamline systematic and scoping review workflows.

For peer review only - http://bmjopen.bmj.com/site/about/guidelines.xhtml

Data Extraction

Data extraction was performed independently by two reviewers (I.M and L.M) using a structured extraction form developed for this review. Any disagreements between the reviewers were resolved through discussion and consensus. The form was piloted to ensure clarity and consistency. Extracted data included article title, authorship and year of publication, country, study design, setting, population characteristics population (including sample size, gender distribution, and mean age), disease condition, adherence assessment tool, validation status, translation or cultural adaptation procedures, administration mode (self-administered or interviewer-administered), timepoint of administration when reported, disease-specific applicability of the instrument, use of additional adherence assessment methods (pill count, pharmacy refill, electronic monitoring), and reported adherence rates.

Data Analysis and Synthesis

The findings were synthesised descriptively and organised into tables to facilitate interpretation and comparison. Given the scope of the review, no subgroup analyses or thematic categorisations were planned or conducted.

Quality Assessment

Consistent with PRISMA-ScR recommendations and the exploratory nature of scoping reviews, formal methodological quality appraisal or risk of bias assessment was not conducted. In fact, the objective of this study was to map evidence rather than determine intervention effectiveness.

Ethics Considerations

This review did not involve the collection of primary data or engagement with human participants and therefore did not require ethical approval.

Patient and Public Involvement

None. Patients and the public were not involved in the design, conduct, reporting, or dissemination plans of this scoping review.

For peer review only - http://bmjopen.bmj.com/site/about/guidelines.xhtml

Results

In total, 6622 records were identified from the following databases: PubMed (n = 666), Web of Science (n = 3522), and Scopus (n = 2434) (Figure 1). Before the initial screening, 1879 duplicate records were removed, leaving 4743 records for the screening stage. No records were excluded by automation tools or for other reasons at this stage. During the screening of the 4743 records, 4452 records were excluded for not meeting the established inclusion criteria. Consequently, 291 full-text reports were selected for retrieval and detailed eligibility assessment. After this stage, 98 studies performed in 27 African countries met all eligibility criteria and were included in the review.

Figure 1: PRISMA flow chart diagram for scoping review on Mapping Medication Adherence Tools in Africa

Study characteristics

Population Demographics

Demographically, the average population size across the studies was approximately 355 participants, exhibiting substantial variation (minimum of 67 and maximum of 4489). The average percentage of female participants was 59.3%. The average age of participants was approximately 46.2 years, with a range of 6.9 to 69.7 years. The reported medication adherence rates varied widely, with an average of 51.8%.

Geographical Distribution

The studies included in this scoping review were conducted across a range of African countries, reflecting the regional diversity of adherence research on chronic conditions. The majority of studies were conducted in Ethiopia (36 studies) [16-51] and Nigeria (22 studies) [52-74], highlighting these countries as key contributors to the evidence base (Figure 2). Egypt accounted for 5 studies [39, 75-79], while Kenya and Ghana were the setting for 5 studies [80-88], Sudan and Cameron were the setting for 4 studies [10, 89-93]. South Africa was the setting for 3 studies [94-96] , Uganda, Senegal, Benim, Togo and Algeria each contributed 2 studies [10, 84-87, 89-92, 95-98]. Single studies were conducted in Eritrea,

For peer review only

For peer review only - http://bmjopen.bmj.com/site/about/guidelines.xhtml

Rwanda, Libya, Benin, Togo, Tanzania, Mozambique, Morocco, Gabon, Guinea, Côte d'Ivoire, Mauritania, Niger, Democratic Republic of Congo, and the Republic of Congo [11, 99-106]. Additionally, one multicountry study spanned Benin, Cameroon, Congo, Democratic Republic of Congo, Gabon, Guinea, Côte d'Ivoire, Mauritania, Mozambique, Niger, Senegal, and Togo [107].

Figure 2:Map of Geographical Distribution of the Studies in Africa

For peer review only

Disease Conditions

The studies included in this scoping review covered a wide range of chronic conditions. The most frequently investigated condition was type 2 diabetes mellitus, reported in 29 studies [11, 17, 28, 33, 34, 36, 37, 43, 48, 60, 64-66, 72, 76-78, 83, 86, 87, 92, 97, 100-102, 104, 108, 109], followed by hypertension, assessed in 18 studies [16, 22, 29, 35, 52, 54, 61, 62, 71, 73, 74, 81, 82, 89, 94, 98, 105, 107, 110]. HIV was examined in 13 studies [44, 50, 53, 63, 68, 80, 82, 85, 88, 95, 99, 103, 111], while epilepsy was the focus of 7 studies [18-21, 25, 90, 96]. Other conditions addressed included type 1 diabetes mellitus (6 studies) [11, 28, 34, 37, 60, 108], depressive disorders (5 studies) [31, 32, 42, 57, 70], schizophrenia (5 studies) [23, 24, 41, 59, 106], asthma (3 studies) [40, 49, 91], glaucoma (3 studies) [45, 55, 84], heart failure (3 studies) [39, 46, 112], rheumatoid arthritis (2 studies) [10, 58], bipolar disorder (1 study) [75], breast cancer (1 study) [27], chronic kidney disease (1 study) [56], Behçet's disease (1 study) [79], kidney transplant recipients (1 study) [38], and psychiatric out-patients (1 study) [67]. Additionally, several studies examined adherence in populations with mixed chronic illnesses or complex comorbidities, such as combinations of hypertension, diabetes mellitus, arthritis, cardiac disease, renal disease, and mental illness [32, 51, 70, 89, 93, 113].

Adherence Assessment Tools

Within the 98 studies included in this scoping review (Supplementary Table 1), a variety of adherence measurement tools were employed. The most frequently used

For peer review only - http://bmjopen.bmj.com/site/about/guidelines.xhtml

instrument was the 8-item Morisky Medication Adherence Scale (MMAS-8), applied in 50 studies [10, 16, 18, 20, 22, 26, 28-30, 34, 37, 42, 43, 45-48, 50, 52, 54, 60-63, 68, 70-72, 75-78, 81, 83, 85, 89, 91, 94, 96, 97, 100, 101, 104, 105, 107]. Other versions of the Morisky scale were also reported, including the 4-item MMAS (MMAS-4, used in 5 studies) [17, 41, 90, 102, 112], the 9-item MMAS (MMAS-9), 1 study [73], and unspecified MMAS versions (5 studies) [21, 25, 55, 56, 59]. The Medication Adherence Report Scale (MARS) was used in 10 studies [23, 24, 31, 32, 40, 57, 65, 87, 106, 110], either alone or combined with other tools. The 5-item MARS (MARS-5) was employed in 6 studies [19, 33, 39, 49, 58, 84]. The Brief Medication Questionnaire (BMQ) appeared in 2 studies, sometimes in combination with MARS or MMAS [11, 66]. Additional tools included the General Medication Adherence Scale (GMAS, 2 studies) [36, 113], Morisky Green Levine Scale (MGLS, 2 studies) [51, 93],the Simplified Medication Adherence Questionnaire (SMAQ, 5 studies) [27, 38, 44, 95, 103], the Medication Adherence Questionnaire (MAQ, 1 study) [86], the Patient Medication Adherence Questionnaire (PMAQ, 1 study) [80], the Compliance Questionnaire on Rheumatology (CQR, 1 study) [79], the Medication Adherence Scale (MAS-8, 1 study) [107], and several studies relied on self-reported adherence, electronic medication monitoring (Mems), pharmacy refill data, or combined methods [67, 82, 88, 98, 99, 111].

Medication Adherence Rates

Medication adherence rates (see online supplementar Table 1) showed wide variation across different diseases analyzed in studies conducted in African countries. This variability may partially reflect differences in adherence measurement tools, cut-off values, administration methods, and lack of local validation. Overall, adherence to treatment was found to be strongly influenced by the type of disease, population context, assessment tools used, and individual patient characteristics.

In the case of asthma, adherence rates ranged from 24.5% to 44.8%, indicating generally low adherence to controller therapy. Among individuals with mixed chronic diseases, values ranged from 48.6% to 50.6%, reflecting moderate levels of therapeutic compliance. For glaucoma, adherence ranged between 46.3% and 60.8%, while among patients with heart failure, rates varied from 36.4% to 76.3%, suggesting differences associated with clinical severity and treatment regimens.

For peer review only

For peer review only - http://bmjopen.bmj.com/site/about/guidelines.xhtml

On HIV, studies reported adherence rates between 26.7% and 96.3%, with higher adherence observed among pediatric populations and lower rates among adolescents and young adults. In cases of arterial hypertension, adherence ranged widely from 3.0% to 96.8%, demonstrating substantial heterogeneity between countries and measurement methodologies.

For other conditions, a study on Behçet's disease reported an adherence rate of 69.2%, while bipolar I disorder showed considerably low adherence at only 19.0%. Among patients with breast cancer undergoing endocrine therapy, adherence reached 70.0%, and in cardiovascular diseases, a rate of 49.0% was observed. Patients with chronic kidney disease demonstrated an adherence rate of 48.6%, whereas in kidney transplant cases, adherence reached 81.9%, reflecting a strong commitment to immunosuppressive therapy.

In mental disorders, adherence ranged from 59.0% to 62.3% for general psychiatric conditions and was 44.3% in combined severe mental disorders. Among patients with depressive disorders, rates ranged from 9.5% to 74.0%, showing high interindividual variability. In epilepsy, adherence varied widely from 4.0% to 65.9%, reflecting specific challenges in disease management.

In rheumatologic conditions, rheumatoid arthritis showed the lowest adherence rates, ranging from 0.0% to 11.8%, indicating significant difficulties in treatment compliance. Schizophrenia exhibited intermediate adherence levels, with rates between 45.0% and 68.4%. In type 1 and type 2 diabetes mellitus, adherence rates were highly variable, ranging from 5.6% to 86.8%, with the lowest values reported in studies conducted in Nigeria and the highest in Ghanaian and Ethiopian contexts.

Methodological characteristics of medication adherence assessment tools

The methodological characteristics of medication adherence assessment tools used in African studies are presented in online supplementary Table 2. Across the included studies, the 8-item Morisky Medication Adherence Scale (MMAS-8) was the most frequently used

For peer review only

For peer review only - http://bmjopen.bmj.com/site/about/guidelines.xhtml

instrument, followed by MARS, SMAQ, MMAS-4, MARS-5 and other less commonly used tools such as GMAS, PMAQ, MCQ, and disease-specific adherence scales.

Interviewer-administered questionnaires represented the predominant mode of administration. Self-administered approaches were less common and included paper questionnaires and electronic adherence tools. Electronic administration methods were reported primarily in HIV-related studies.

Most instruments were generic rather than disease-specific. Disease-specific tools were identified mainly in studies involving HIV, rheumatologic conditions and selected chronic diseases. Additionally, only a small number of studies employed supplementary adherence assessment methods such as pill counts, pharmacy refill records, medication possession ratios, viral load monitoring, biological markers, therapeutic drug monitoring and electronic medication monitoring systems.

Validation and Cultural Adaptation of Adherence Tools

Within the 98 studies included in this scoping review, only 15 (only 15.3% of the studies) reported having undertaken translation and/or cultural adaptation of the instruments to the local context. Among the included studies, eight validated the MMAS-8 across different African countries [22, 31, 52, 75, 76, 97, 104, 105], while one study validated the MMAS-4 [112],two validated the MARS-5 [39, 49], two validated the MGLS [51, 93] and two validated the MARS [23, 32].

Translation and cultural adaptation procedures varied considerably across studies. 40/98 (40.8%) studies translated tools into local languages such as Amharic, Afaan Oromo, Arabic, Yoruba, Kiswahili, Runyankore/Rukiga, Igbo, French and Lingala, while 43/98 (43.9%) studies did not report any adaptation process. Translation was frequently mentioned without detailed descriptions of forward backward translation procedures or psychometric reassessment after adaptation. Discussion

This scoping review mapped medication adherence assessment tools used across African populations with chronic diseases and identified substantial methodological

For peer review only

For peer review only - http://bmjopen.bmj.com/site/about/guidelines.xhtml

heterogeneity in adherence measurement. A total of 98 studies conducted in 27 African countries were identified, with the MMAS-8 being the most frequently used scales, followed by MARS and other scales such as SMAQ, MCQ, PMAQ and CQR, used in specific contexts.

Adherence rates showed high variability among the 98 studies included in this scoping review, ranging from 0% to 96.8%, depending on the country, the health condition, and the instrument used. This wide discrepancy may reflect both methodological challenges such as the use of non-standardised instruments and contextual factors, such as the type of disease, the age group of participants, and the characteristics of the health system in place [107]. Chronic diseases such as hypertension and diabetes generally showed lower adherence rates, which is consistent with African studies [104, 105]. On the other hand, conditions such as infection with the HIV infection demonstrated relatively higher adherence rates in some contexts, possibly due to greater investment in adherence programs in countries like Kenya and South Africa [114, 115].

The widespread use of the MMAS-8 adherence scale was consistent with findings from studies conducted in various African contexts, as well as international studies, which highlight its simplicity, practicality, reliability, and applicability across multiple clinical conditions [116, 117]. This scale remains one of the most widely used self-report adherence measures due to its simplicity, low implementation cost, and ease of administration [118]. However, the large number of instruments identified, including MMAS-4, MARS, SMAQ, GMAS, BMQ, MAQ, PMAQ, CQR, and several indirect methods, reflects the absence of a standardized approach for adherence measurement in African settings [2]. This methodological diversity may partly explain the substantial variability observed in adherence estimates across studies.

In most studies, the absence of validation of adherence tools within local African contexts was identified [117]. Many scales were applied directly or merely translated, without proper linguistic, sociocultural adaptation or local psychometric validation, disregarding the linguistic diversity and cultural specificities of the continent [104, 119]. For example, one study conducted in Ethiopia used the MMAS-8 translated into the local language without formal psychometric validation [116]. This practice may introduce interpretation bias, particularly among populations with low health literacy, making it

For peer review only - http://bmjopen.bmj.com/site/about/guidelines.xhtml

difficult to understand terms such as "adherence" or "regular use." Although adherence scales that are translated, culturally adapted, and locally validated can be feasible and reliable, there was a notable prevalence of imported tools used without local validation. In Morocco and Sudan, for instance, the GMAS was validated; however, other scales still lack regional and local adaptations [119, 120].

Despite the widespread use of the MMAS-8 adherence scale, only eight studies conducted in Africa have reported formal psychometric and cultural validation of this adherence tool [22, 31, 52, 75, 76, 97, 104, 105]. Formal validation is essential to ensure that the instrument truly measures the construct of adherence within the diverse African contexts, which are characterized by linguistic diversity, health literacy barriers, and cultural influence [105, 116, 120, 121]. In most studies, the MMAS-8 was only directly translated, without consistent psychometric validation, raising concerns about the reliability of the information produced and its applicability in monitoring multiple clinical conditions in African settings. Studies conducted in Ethiopia and Nigeria reported semantic comprehension difficulties with the MMAS-8, requiring modifications to the scale's content to ensure clarity without compromising the essence of the original instrument [104, 122-124]. This diversity reflects the lack of standardisation in adherence assessment and highlights the need for careful consideration when comparing results across studies.

Most of the adherence scales identified were developed outside the African continent, with cultural and linguistic validations still insufficiently established in the populations where they were applied. This raises concerns regarding the cultural sensitivity and contextual appropriateness of these scales to accurately measure treatment adherence across diverse African contexts.

Beyond validation status, the findings raise important questions regarding the appropriateness of adherence instruments across different disease conditions. Most studies relied on generic self-report tools regardless of the clinical condition under investigation [2]. While instruments such as MMAS and MARS are practical and easy to administer, they may not adequately capture disease-specific adherence challenges [125]. Medication-taking behaviour among individuals living with HIV differs considerably from that observed in patients with diabetes, hypertension, epilepsy, or severe mental illness. Disease-specific tools such as SMAQ for HIV or CQR for rheumatologic diseases may provide more clinically

For peer review only - http://bmjopen.bmj.com/site/about/guidelines.xhtml

relevant information because they address treatment characteristics unique to these conditions[126]. The widespread use of generic instruments identified in this review suggests that important dimensions of adherence may remain insufficiently assessed in several African contexts, where cultural adaptations and disease-specific validation remain sparse [116].

Most studies on adherence scales included in this scoping review focus on the regions of West and East Africa, with emphasis on Ethiopia, Nigeria, Egypt, Kenya, and Cameroon, while countries in the North and South of the continent, such as Libya, Tunisia, Angola, Mozambique, and South Africa, were underrepresented or not represented at all. This may indicate a disparity in the geographical distribution of scientific production related to adherence scales across different African countries, with some countries having higher research output than others possibly due to more developed research infrastructures [119]. This unequal distribution may create gaps in regional understanding of the barriers and facilitators to adherence, compromising the development of public policies tailored to each context [127].

The predominance of studies conducted in hospital settings, rather than in community or rural environments, may limit the generalizability of the results, especially on a continent where most of the population lives outside urban centres. This type of study requires clinical infrastructure to ensure strict protocol control, which favours research conducted in hospital environments [128].

The findings of this review have important implications for both research and clinical practice. Future studies should prioritize the development and validation of culturally appropriate adherence instruments capable of capturing the realities of African populations. Greater standardization of adherence measurement would improve comparability across studies and support evidence-based decision-making. In addition, combining self-reported measures with objective methods such as pharmacy refill records, biological markers, or electronic monitoring may improve measurement accuracy.

This review has several strengths. It represents, the first comprehensive mapping of medication adherence assessment tools used across African populations with chronic diseases. The review followed PRISMA-ScR recommendations and included evidence from a broad range of countries, diseases, and healthcare settings.

For peer review only - http://bmjopen.bmj.com/site/about/guidelines.xhtml

This study has some limitations, although a systematic search strategy was conducted across various databases for this scoping review, it is possible that some relevant studies were not identified, the studies published in English and French were included, potentially excluding relevant evidence published in other languages. As recommended for scoping reviews, no formal methodological quality appraisal or risk-of-bias assessment was conducted. The heterogeneity among the studies in terms of clinical conditions and populations assessed, application contexts, and languages used may limit comparisons between scales and the generalization of results to different African settings. The geographical distribution of studies was uneven, with a strong concentration in Ethiopia and Nigeria and limited evidence from several African countries, which may affect the generalizability of findings across the continent.

In addition, although major international databases such as PubMed were included, some studies indexed exclusively in African Journals Online (AJOL), may not have been captured.

Conclusion

In conclusion, this review has identified that important challenges remain in terms of tools to assess adherence in Africa due to the lack of standardised, validated, and culturally adapted tools. The predominant use of the MMAS-8 adherence scale, without formal psychometric validation, raises concerns about the reliability and applicability of the data obtained across different linguistic, cultural, and social contexts on the continent.

Future research should prioritize the contextual adaptation, psychometric validation, and methodological standardization of existing adherence assessment tools before developing entirely new instruments for African settings. Such tools should be integrated into national and regional health strategies, with guidelines to direct adaptation and psychometric validation, in order to strengthen systems for monitoring treatment adherence, improve the guidance of therapeutic interventions, and promote more accurate and equitable public policies tailored to the diverse contexts across the continent.

For peer review only

Acknowledgements

Acknowledgments are extended to the Faculties of Medicine and Pharmacy of the University of Porto for the scientific support that made this scoping review possible.

Special thanks are given to the Universidade São Tomás de Moçambique for the moral support during the continuation of the doctoral studies.

Author contribuitions

Conceptualization: Izaidino Muchanga, Luís Midão, Elísio Costa and Bernardo Sousa-Pinto

Data curation: Izaidino Muchanga, Luís Midão, Avelino Mazuze, Daniela Melo, Leovaldo Alcântara, and Teodora Figueiredo.

Writing-original draft: Izaidino Muchanga and Luís Midão

Supervision: Elísio Costa and Bernardo Sousa-Pinto

Writing-review & editing: Izaidino Muchanga, Luís Midão, Avelino Mazuze, Daniela Melo, Leovaldo Alcântara, Teodora Figueiredo, Elísio Costa and Bernardo Sousa-Pinto

Izaidino Muchanga is the guarantor and accepts full responsibility for the overall content of the work, including the integrity of the data, the accuracy of the analyses, and the decision to submit the manuscript for publication.

Funding

This work was supported by the Fundação para a Ciência e a Tecnologia (FCT), Portugal, through a PhD scholarship granted to Izaidino Jaime Muchanga (PRT/BD/154774/2022-https://doi.org/10.54499/PRT/BD/154774/2022), funded by national funds, within the scope of the PhD program in Clinical and Health Services Research at the Faculty of Medicine of the University of Porto.

The funder had no role in the design of the study; the collection, analysis, or interpretation of data; the writing of the manuscript; or the decision to submit the article for publication.

Competing Interests

The authors declare that they have no competing interests.

For peer review only - http://bmjopen.bmj.com/site/about/guidelines.xhtml

References

1. Al-Moamary M, Aggarwal B, Al-Ahmad M, Sriprasart T, Koenig S, Levy G, et al. Are Treatment Adherence Factors Apparent in Patients with Asthma and to Physicians? Results from the APPaRENT 3 Survey. Adv Ther. 2025;42(3):1506–21.
2. Kardas P, Bennett B, Borah B, Burnier M, Daly C, Hiligsmann M, et al. Medication non-adherence: reflecting on two decades since WHO adherence report and setting goals for the next twenty years. Front Pharmacol. 2024;15:1444012.
3. Berardinelli D, Conti A, Hasnaoui A, Casabona E, Martin B, Campagna S, et al. Nurse-Led Interventions for Improving Medication Adherence in Chronic Diseases: A Systematic Review. Healthcare (Basel). 2024;12(23).
4. Egekeze CO, Chukwu C, Vieira D, Gyamfi J, Allegrante JP. Assessing implementation outcomes from studies addressing medication adherence for hypertension management in Sub-Saharan Africa: a systematic review. BMC Public Health. 2025;25(1):4085.
5. Baby B, McKinnon A, Patterson K, Patel H, Sharma R, Carter C, et al. Tools to measure barriers to medication management capacity in older adults: a scoping review. BMC Geriatr. 2024;24(1):285.
6. Achterbosch M, Aksoy N, Obeng GD, Ameyaw D, Agh T, van Boven JFM. Clinical and economic consequences of medication nonadherence: a review of systematic reviews. Front Pharmacol. 2025;16:1570359.
7. Gouda HN, Charlson F, Sorsdahl K, Ahmadzada S, Ferrari AJ, Erskine H, et al. Burden of non-communicable diseases in sub-Saharan Africa, 1990-2017: results from the Global Burden of Disease Study 2017. Lancet Glob Health. 2019;7(10):e1375–e87.
8. Dunbar-Jacob J, Zhao J. Medication Adherence Measurement in Chronic Diseases: A State-of-the-Art Review of the Literature. Nurs Rep. 2025;15(10).
9. Ukoaka BM, Ugwuanyi EA, Ukueku KO, Ajah KU, Udam NG, Daniel FM, et al. Digital tools for improving antiretroviral adherence among people living with HIV in Africa. Journal of Medicine, Surgery, and Public Health. 2024;2.
10. Ali ZAH, Abd El-Raheem GOH, Noma M. Status of rheumatoid arthritis practice and treatment in Sudan. Scientific African. 2023;22.
11. Olorunfemi O. Health literacy, medication belief, and demographic factors as correlates of medication adherence in person with diabetes mellitus. Journal of Diabetology. 2023;14(2):100–6.
12. Page MJ, McKenzie JE, Bossuyt PM, Boutron I, Hoffmann TC, Mulrow CD, et al. The PRISMA 2020 statement: an updated guideline for reporting systematic reviews. BMJ. 2021;372:n71.
13. Arksey H, O'Malley L. Scoping studies: towards a methodological framework. International Journal of Social Research Methodology. 2005;8(1):19–32.
14. O'Brien LDHCKK. Scoping studies: advancing the methodology. Implementation Science. 2010.
15. Ouzzani M, Hammady H, Fedorowicz Z, Elmagarmid A. Rayyan-a web and mobile app for systematic reviews. Syst Rev. 2016;5(1):210.

For peer review only - http://bmjopen.bmj.com/site/about/guidelines.xhtml

16. D GT, Berhane Y, Worku A. Adherence to Antihypertensive Treatment and Associated Factors in Central Ethiopia. Int J Hypertens. 2020;2020:9540810.

17. Jemal A, Abdela J. Adherence to Oral Antidiabetic Medications among Type 2 Diabetic (T2DM) Patients in Chronic Ambulatory Wards of Hiwot Fana Specialized University Hospital, Harar, Eastern Ethiopia: A Cross Sectional Study. Journal of Diabetes & Metabolism. 2017;08(01).

18. Hasiso TY, Desse TA. Adherence to Treatment and Factors Affecting Adherence of Epileptic Patients at Yirgalem General Hospital, Southern Ethiopia: A Prospective Cross-Sectional Study. PLoS One. 2016;11(9):e0163040.

19. Shumet S, Wondie M, Ayano G, Asfaw H, Kassew T, Mesafint G. Antiepileptic Drug Adherence and Its Associated Factors among Epilepsy Patients on Follow-ups at Amanuel Mental Specialized Hospital, Ethiopia. Ethiop J Health Sci. 2022;32(5):913–22.

20. Dima SA, Shibeshi MS. Antiepileptic drug adherence in children in southern Ethiopia: A cross sectional study. PLoS One. 2022;17(2):e0263821.

21. Getnet A, Woldeyohannes SM, Bekana L, Mekonen T, Fekadu W, Menberu M, et al. Antiepileptic Drug Nonadherence and Its Predictors among People with Epilepsy. Behav Neurol. 2016;2016:3189108.

22. Asgedom SW, Atey TM, Desse TA. Antihypertensive medication adherence and associated factors among adult hypertensive patients at Jimma University Specialized Hospital, southwest Ethiopia. BMC Res Notes. 2018;11(1):27.

23. Tareke M, Tesfaye S, Amare D, Belete T, Abate A. Antipsychotic medication non-adherence among schizophrenia patients in Central Ethiopia. S Afr J Psychiatr. 2018;24:1124.

24. Mohammed F, Geda B, Yadeta TA, Dessie Y. Antipsychotic medication non-adherence and factors associated among patients with schizophrenia in eastern Ethiopia. BMC Psychiatry. 2024;24(1):108.

25. Kassahun G, Moges G, Demessie Y. Assessment of Patients' Adherence to Antiepileptic Medications at Dessie Referral Hospital, Chronic Follow-Up, South Wollo, Amhara Region, North East Ethiopia. Neurol Res Int. 2018;2018:5109615.

26. Abebe K, Deresse B, Negeri KG. Non adherence to treatment and the associated factors in patients with epilepsy in Southern Ethiopia. Healthcare in Low-resource Settings. 2022;10(1).

27. Getachew S, Addissie A, Seife E, Wakuma T, Unverzagt S, Jemal A, et al. Breast Nurse Intervention to Improve Adherence to Endocrine Therapy Among Breast Cancer Patients in South Ethiopia. Oncologist. 2022;27(8):e650–e60.

28. Demoz GT, Berha AB, Alebachew Woldu M, Yifter H, Shibeshi W, Engidawork E. Drug therapy problems, medication adherence and treatment satisfaction among diabetic patients on follow-up care at Tikur Anbessa Specialized Hospital, Addis Ababa, Ethiopia. PLoS One. 2019;14(10):e0222985.

29. Berhe DF, Taxis K, Haaijer-Ruskamp FM, Mulugeta A, Mengistu YT, Burgerhof JGM, et al. Impact of adverse drug events and treatment satisfaction on patient adherence with antihypertensive medication - a study in ambulatory patients. Br J Clin Pharmacol. 2017;83(9):2107–17.

30. Shimels T, Asrat Kassu R, Bogale G, Bekele M, Getnet M, Getachew A, et al. Magnitude and associated factors of poor medication adherence among diabetic and

For peer review only - http://bmjopen.bmj.com/site/about/guidelines.xhtml

hypertensive patients visiting public health facilities in Ethiopia during the COVID-19 pandemic. PLoS One. 2021;16(4):e0249222.

31. Alemayehu Y, Asfaw H, Girma M. Magnitude and factors associated with treatment non-adherence among patients with depressive disorders at St. Amanuel Mental Specialized Hospital, Addis Ababa, Ethiopia, 2019: A cross sectional study. PLoS One. 2022;17(7):e0271713.

32. Gudeta DB, Leta K, Alemu B, Kandula UR. Medication adherence and associated factors among psychiatry patients at Asella Referral and Teaching Hospital in Oromia, Ethiopia: Institution based cross sectional study. PLoS One. 2023;18(4):e0283829.

33. Yosef T, Nureye D, Tekalign E, Assefa E, Shifera N. Medication Adherence and Contributing Factors Among Type 2 Diabetes Patients at Adama Hospital Medical College in Eastern Ethiopia. SAGE Open Nurs. 2023;9:23779608231158975.

34. Ali M, Alemu T, Sada O. Medication adherence and its associated factors among diabetic patients at Zewditu Memorial Hospital, Addis Ababa, Ethiopia. BMC Res Notes. 2017;10(1):676.

35. Teshome DF, Bekele KB, Habitu YA, Gelagay AA. Medication adherence and its associated factors among hypertensive patients attending the Debre Tabor General Hospital, northwest Ethiopia. Integr Blood Press Control. 2017;10:1–7.

36. Sendekie AK, Netere AK, Kasahun AE, Belachew EA. Medication adherence and its impact on glycemic control in type 2 diabetes mellitus patients with comorbidity: A multicenter cross-sectional study in Northwest Ethiopia. PLoS One. 2022;17(9):e0274971.

37. Abate TW. Medication non-adherence and associated factors among diabetes patients in Felege Hiwot Referral Hospital, Bahir Dar city administration, Northwest Ethiopia. BMC Res Notes. 2019;12(1):175.

38. Derejie MN, Dereje EN, Alemu DM, Tesfay YG, Hunduma F, Temie NM. Medication non-adherence and its associated factors among kidney transplant patients in a large teaching hospital in Ethiopia. BMC Nephrol. 2024;25(1):187.

39. Seid MA, Toleha HN, Sema FD. Medication Nonadherence and Associated Factors among Heart Failure Patients at University of Gondar Comprehensive Specialized Hospital, Northwest Ethiopia. Int J Chronic Dis. 2023;2023:1824987.

40. Belachew EA, Netere AK, Sendekie AK. Medication regimen complexity and its impact on medication adherence and asthma control among patients with asthma in Ethiopian referral hospitals. Asthma Res Pract. 2022;8(1):7.

41. Desalegn D, Girma S, Abdeta T. Quality of life and its association with current substance use, medication non-adherence and clinical factors of people with schizophrenia in Southwest Ethiopia: a hospital-based cross-sectional study. Health Qual Life Outcomes. 2020;18(1):82.

42. Abegaz TM, Sori LM, Toleha HN. Self-Reported Adverse Drug Reactions, Medication Adherence, and Clinical Outcomes among Major Depressive Disorder Patients in Ethiopia: A Prospective Hospital Based Study. Psychiatry J. 2017;2017:5812817.

43. Erku DA, Ayele AA, Mekuria AB, Belachew SA, Hailemeskel B, Tegegn HG. The impact of pharmacist-led medication therapy management on medication adherence in patients with type 2 diabetes mellitus: a randomized controlled study. Pharm Pract (Granada). 2017;15(3):1026.

For peer review only

For peer review only - http://bmjopen.bmj.com/site/about/guidelines.xhtml

44. Wedajo S, Degu G, Deribew A, Ambaw F. The role of health facility and individual level characteristics on medication adherence among PLHIV on second-line antiretroviral therapy in Northeast Ethiopia: use of multi-level model. AIDS Res Ther. 2022;19(1):17.
45. Birhanie SA, Getie GA, Tesfa M, Mulugeta H, Gedfew M, Mekete YS, et al. Treatment adherence and associated factors among glaucoma patients attending Ophthalmic units of referral hospitals in North West Ethiopia, 2019. Front Ophthalmol (Lausanne). 2022;2:985893.
46. Mohammed AS, Kemal WS, Kolbay AS, Yesuf MH, Abebe SS, Solomon B, et al. Medication Adherence and Its Determinants Among Adults With Heart Failure: A Study in Selected Hospitals of North Shewa Zone, Oromia, Ethiopia (2024)-A Multicenter Institutional-Based Cross-Sectional Study. Adv Rehabil Sci Pract. 2026;15:27536351251391632.
47. Kassaw AT, Tarekegn TB, Derbie A, Ashagrie G, Girmaw F, Mengesha A. Non-adherence to antidiabetic medications and associated factors among adult type 2 diabetes mellitus patients in Northeast Ethiopia: institutional based cross-sectional study. BMJ Open. 2025;15(4):e093413.
48. Kassaw EA, Sendekie AK, Enyew BM, Abate BB. Machine learning applications to classify and monitor medication adherence in patients with type 2 diabetes in Ethiopia. 2025;16.
49. Gebremariam GT, Hayiso MH, Moon Z, Horne R, Yusuf H, Kebede RA, et al. Reliability of the Amharic Version of the Medication Adherence Report Scale and Beliefs about Medicines Questionnaire in Patients with Asthma in Ethiopia. 2025;35(1):17–28.
50. Tarekegn TB, Gebrie D, Kassaw AT, Adela B, Lulseged K, Girmaw F, et al. Cultural adaptation and psychometric validation of the Amharic adherence barriers questionnaire for HIV patients on antiretroviral therapy in Ethiopia. Sci Rep. 2026;16(1).
51. Tegegn HG, Wark S, Tursan D'espaignet É, Spark MJ. Validation of Amharic versions of medication adherence tools among outpatients with cardiovascular diseases: a cross-sectional study. 2025;16(1).
52. Okwuonu Chimezie G UNV, Chimezie Oluchi Jb , Ogah Okechukwu S , Mbanaso Augustus U , Odigwe Clement O. ADHERENCE TO ANTIHYPERTENSIVE MEDICATION AND ITS CORRELATES AMONG INDIVIDUALS WITH HYPERTENSION IN A SEMI-URBAN COMMUNITY

OF SOUTHERN NIGERIA. Journal of Biomedical Sciences. 2015;14:12.
53. Abah IO, Ojeh VB, Musa J, Ugoagwu P, Agaba PA, Agbaji O, et al. Clinical Utility of Pharmacy-Based Adherence Measurement in Predicting Virologic Outcomes in an Adult HIV-Infected Cohort in Jos, North Central Nigeria. J Int Assoc Provid AIDS Care. 2016;15(1):77–83.
54. Okunrinboye HI, Otakpor AN, Ilesanmi OS. Depression and medication-adherence in patients with hypertension attending a tertiary health facility in South-West Nigeria. Pan Afr Med J. 2019;33:27.
55. Bello SI, Ojieabu WA, Yusuf H. Drug utilization, medication adherence and treatment satisfaction amongst glaucoma patients receiving care at secondary health facilities. Journal of Pharmaceutical Health Services Research. 2023;14(1):71–8.

For peer review only - http://bmjopen.bmj.com/site/about/guidelines.xhtml

56. Okoro RN, Ummate I, Ohieku JD, Yakubu S, Adibe MO, Okonta MJ. Evaluation of medication adherence and predictors of sub-optimal adherence among pre-dialysis patients with chronic kidney disease. Med Access Point Care. 2020;4:2399202620954089.
57. Yusuf H, Magaji MG, Maiha BB, Yakubu SI, Haruna WC, Mohammed S. Impact of pharmacist intervention on antidepressant medication adherence and disease severity in patients with major depressive disorder in fragile north-east Nigeria. Journal of Pharmaceutical Health Services Research. 2021;12(3):410–6.
58. Ubaka CM, Ibe OG, Amorha KC, Isah A, Okonta MJ. Medication adherence among Nigerian patients with rheumatoid arthritis: a two instruments survey. Journal of Pharmaceutical Health Services Research. 2021;12(1):11–7.
59. Ogunnubi OP, Olagunju AT, Aina OF, Okubadejo NU. Medication Adherence Among Nigerians with Schizophrenia: Correlation Between Clinico-Demographic Factors and Quality of Life. Ment Illn. 2017;9(1):6889.
60. Fadare J, Olamoyegun M, Gbadegesin BA. Medication adherence and direct treatment cost among diabetes patients attending a tertiary healthcare facility in Ogbomosho, Nigeria. Malawi Med J. 2015;27(2):65–70.
61. Oluwole EO, Osibogun O, Adegoke O, Adejimi AA, Adewole AM, Osibogun A. Medication adherence and patient satisfaction among hypertensive patients attending outpatient clinic in Lagos University Teaching Hospital, Nigeria. Niger Postgrad Med J. 2019;26(2):129–37.
62. Olaniran GO, Akodu BA, Olaniran AA, Bamidele JO, Ogunyemi AO, Idowu OO. Medication Adherence and Perceived Family Support Among Elderly Patients with Hypertension Attending a Specialty Clinic in Lagos, Nigeria. Annals of Health Research. 2023;9(1):30–42.
63. Aduloju OP, Aduloju, T., Ade-Ojo, I. P., & Akintayo, A. A. Medication adherence in HIV-positive pregnant women on antiretroviral therapy attending antenatal clinics in Ado metropolis, south-west Nigeria: A multicentre study. S Afr J Obstet Gynaecol. 2020;26.
64. Jackson IL, Adibe MO, Okonta MJ, Ukwe CV. Medication adherence in type 2 diabetes patients in Nigeria. Diabetes Technol Ther. 2015;17(6):398–404.
65. Awodele O, Osuolale JA. Medication adherence in type 2 diabetes patients: study of patients in Alimosho General Hospital, Igando, Lagos, Nigeria. Afr Health Sci. 2015;15(2):513–22.
66. Olorunfemi O, Ojewole F. Medication belief as correlate of medication adherence among patients with diabetes in Edo State, Nigeria. Nurs Open. 2019;6(1):197–202.
67. Okpataku CI, Kwanashie HO, Ejiofor JI, Olisah VO. Medication compliance behavior in psychiatric out-patients with psychoactive substance use comorbidity in a Nigerian tertiary hospital. Niger J Clin Pract. 2015;18(3):371–6.
68. Seb-Akahomen O, Lawani A, James B. Mood and anxiety disorders adversely influence medication adherence to antiretroviral therapy among people living with HIV/AIDS in Nigeria. HIV & AIDS Review. 2018;17(2):91–7.
69. Heestermans T, Browne JL, Aitken SC, Vervoort SC, Klipstein-Grobusch K. Determinants of adherence to antiretroviral therapy among HIV-positive adults in sub-Saharan Africa: a systematic review. BMJ Glob Health. 2016;1(4):e000125.

For peer review only - http://bmjopen.bmj.com/site/about/guidelines.xhtml

70. Ibrahim AW, Yahya S, Pindar SK, Wakil MA, Garkuwa A, Sale S. Prevalence and predictors of sub-optimal medication adherence among patients with severe mental illnesses in a tertiary psychiatric facility in Maiduguri, North-eastern Nigeria. Pan Afr Med J. 2015;21:39.
71. Ipinnimo TM, Abodunrin OL, Durowade KA. Relationship between Cost of Care and Medication Adherence among Hypertensive Patients Attending a Tertiary Health Facility in Ekiti State, Nigeria. Journal of Community Medicine and Primary Health Care. 2022;34(1):38–57.
72. Ajibola SS, Timothy FO. The Influence of National Health Insurance on Medication Adherence Among Outpatient Type 2 Diabetics in Southwest Nigeria. J Patient Exp. 2018;5(2):114–9.
73. Adisa R, Ilesanmi OA, Fakeye TO. Treatment adherence and blood pressure outcome among hypertensive out-patients in two tertiary hospitals in Sokoto, Northwestern Nigeria. BMC Cardiovasc Disord. 2018;18(1):194.
74. Akpor OA, Olowolaju FO. Treatment satisfaction and medication adherence among hypertensive patients in a teaching hospital in Ekiti State, Nigeria. Journal of Integrative Nursing. 2022;4(2):59–67.
75. Okasha TA, Radwan DN, Elkholy H, Hendawy H, Shourab E, Teama RRA, et al. Psycho-demographic and clinical predictors of medication adherence in patients with bipolar I disorder in a university hospital in Egypt. S Afr J Psychiatr. 2020;26:1437.
76. Salama HM, Saudi RA. Effect of patients beliefs about medications on adherence to drugs in diabetic patients attending family medicine outpatient clinic in Ismailia, Egypt. J Diabetes Metab Disord. 2020;19(2):951–8.
77. Saudi RA, Abbas RA, Nour-Eldein H, Sayed Ahmed HA. Illness perception, medication adherence and glycemic control among primary health-care patients with type 2 diabetes mellitus at Port Said City, Egypt. Diabetol Int. 2022;13(3):522–30.
78. Sayed Ahmed HA, Abdelsalam NE, Joudeh AI, Abdelrahman AG, Eldahshan NA. Association of treatment satisfaction and physician trust with glycemic control among primary care patients with type 2 diabetes in Egypt. Diabetol Int. 2024;15(1):67–75.
79. Zayed HS, Medhat BM, Seif EM. Evaluation of treatment adherence in patients with Behcet's disease: its relation to disease manifestations, patients' beliefs about medications, and quality of life. Clin Rheumatol. 2019;38(3):761–8.
80. Kioko MT, Pertet AM. Factors contributing to antiretroviral drug adherence among adults living with HIV or AIDS in a Kenyan rural community. Afr J Prim Health Care Fam Med. 2017;9(1):e1–e7.
81. Xiong S, Peoples N, Ostbye T, Olsen M, Zhong X, Wainaina C, et al. Family support and medication adherence among residents with hypertension in informal settlements of Nairobi, Kenya: a mixed-method study. J Hum Hypertens. 2023;37(1):74–9.
82. Vreeman RC, Nyandiko WM, Liu H, Tu W, Scanlon ML, Slaven JE, et al. Measuring adherence to antiretroviral therapy in children and adolescents in western Kenya. J Int AIDS Soc. 2014;17(1):19227.
83. Waari G, Mutai J, Gikunju J. Medication adherence and factors associated with poor adherence among type 2 diabetes mellitus patients on follow-up at Kenyatta National Hospital, Kenya. Pan Afr Med J. 2018;29:82.

For peer review only - http://bmjopen.bmj.com/site/about/guidelines.xhtml

84. Abu EK, Antiri EO, Ocansey S, Ntodie M, Abokyi S, Abraham CH. Associations between personality traits and adherence to treatment in patients with primary open-angle glaucoma in an African population. Clin Exp Optom. 2023;106(5):509–15.

85. Adu C, Mensah KA, Ahinkorah BO, Osei D, Tetteh AW, Seidu AA. Socio-demographic factors associated with medication adherence among People Living with HIV in the Kumasi Metropolis, Ghana. AIDS Res Ther. 2022;19(1):50.

86. Afaya RA, Bam V, Azongo TB, Afaya A, Kusi-Amponsah A, Ajusiyine JM, et al. Medication adherence and self-care behaviours among patients with type 2 diabetes mellitus in Ghana. PLoS One. 2020;15(8):e0237710.

87. Kretchy IA, Koduah A, Ohene-Agyei T, Boima V, Appiah B. The Association between Diabetes-Related Distress and Medication Adherence in Adult Patients with Type 2 Diabetes Mellitus: A Cross-Sectional Study. J Diabetes Res. 2020;2020:4760624.

88. Mayamba WO, Onguru D, Ogolla S. FACTORS INFLUENCING ADHERENCE TO ANTIRETROVIRAL THERAPY AMONG ADOLESCENTS AT BUSIA COUNTY REFERRAL HOSPITAL (KENYA). 2025;102(10):8784–97.

89. Awad A, Osman N, Altayib S. Medication adherence among cardiac patients in Khartoum State, Sudan: a cross-sectional study. Cardiovasc J Afr. 2017;28(6):350–5.

90. Elsayed MA, El-Sayed NM, Badi S, Ahmed MH. Factors affecting adherence to antiepileptic medications among Sudanese individuals with epilepsy: A cross-sectional survey. J Family Med Prim Care. 2019;8(7):2312–7.

91. Ngahane BH, Pefura-Yone EW, Mama M, Tengang B, Nganda MM, Wandji A, et al. Evaluation of factors affecting adherence to asthma controller therapy in chest clinics in a sub-Saharan African setting: a cross-sectional study. Afr Health Sci. 2016;16(1):194–200.

92. Aminde LN, Tindong M, Ngwasiri CA, Aminde JA, Njim T, Fondong AA, et al. Adherence to antidiabetic medication and factors associated with non-adherence among patients with type-2 diabetes mellitus in two regional hospitals in Cameroon. BMC Endocr Disord. 2019;19(1):35.

93. Mahmoud AA, Saeed AA, Elnour AA, Nasreldin EMO, Menon V, Beshir SA, et al. A cross-sectional study on the assessment of adherence to cardiovascular medications in Sudan heart center. PLOS ONE. 2025;20(1).

94. Murendeni F. Sikhau MJM, Gert J.O. Marincowitz and Clara Marincowitz. Adherence to hypertension management at a rural hospital in Limpopo: A cross-sectional study. South African Family Practice. 2025:7.

95. Hirasen K, Evans D, Jinga N, Grabe R, Turner J, Mashamaite S, et al. Using a Self-Administered Electronic Adherence Questionnaire to Identify Poor Adherence Amongst Adolescents and Young Adults on First-Line Antiretroviral Therapy in Johannesburg, South Africa. Patient Prefer Adherence. 2020;14:133–51.

96. Egenasi C, Steinberg WJ, Raubenheimer JE. Beliefs about medication, medication adherence and seizure control among adult epilepsy patients in Kimberley, South Africa. South African Family Practice. 2015;57(5):326–32.

97. Achouri MY, Mammeri M, Sehanine Y, Selka MA, Ghomari WI, Lahmer A, et al. [Factors associated with medication non-adherence in patients with type 2 diabetes mellitus: The first Algerian survey]. Ann Pharm Fr. 2019;77(6):506–15.

98. Kara S. General self-efficacy and hypertension treatment adherence in Algerian private clinical settings. J Public Health Afr. 2022;13(3):2121.

For peer review only - http://bmjopen.bmj.com/site/about/guidelines.xhtml

99. Musumari PM, Wouters E, Kayembe PK, Kiumbu Nzita M, Mbikayi SM, Suguimoto SP, et al. Food insecurity is associated with increased risk of non-adherence to antiretroviral therapy among HIV-infected adults in the Democratic Republic of Congo: a cross-sectional study. PLoS One. 2014;9(1):e85327.

100. Ashur ST, Shah SA, Bosseri S, Morisky DE, Shamsuddin K. Illness perceptions of Libyans with T2DM and their influence on medication adherence: a study in a diabetes center in Tripoli. Libyan J Med. 2015;10(1):29797.

101. Doya IF, Yahaya JJ, Ngaiza AI, Bintabara D. Low medication adherence and its associated factors among patients with type 2 diabetes mellitus attending Amana Hospital in Dar es Salaam, Tanzania: a cross-sectional study. Int Health. 2024;16(2):200–7.

102. Murwanashyaka JD, Ndagijimana A, Biracyaza E, Sunday FX, Umugwaneza M. Non-adherence to medication and associated factors among type 2 diabetes patients at Clinique Medicale Fraternite, Rwanda: a cross-sectional study. BMC Endocr Disord. 2022;22(1):219.

103. Mbengue MAS, Sarr SO, Diop A, Ndour CT, Ndiaye B, Mboup S. Prevalence and determinants of adherence to antiretroviral treatment among HIV patients on first-line regimen: a cross-sectional study in Dakar, Senegal. Pan Afr Med J. 2019;33:95.

104. Tandon S, Chew M, Eklu-Gadegbeku CK, Shermock KM, Morisky DE. Validation and psychometric properties of the 8-item Morisky Medication Adherence Scale (MMAS-8) in Type 2 diabetes patients in sub-Saharan Africa. Diabetes Res Clin Pract. 2015;110(2):129–36.

105. Okello S, Nasasira B, Muiru AN, Muyingo A. Validity and Reliability of a Self-Reported Measure of Antihypertensive Medication Adherence in Uganda. PLoS One. 2016;11(7):e0158499.

106. Doufik J, Lahlou L, El Oumary O, Salehddine Z, Tbatou A, El Abbani M, et al. Impact of sociodemographic factors and depression on medication adherence in schizophrenia: Cross sectional study in Morocco. Arch Psychiatr Nurs. 2024;53:62–70.

107. Macquart de Terline D, Kane A, Kramoh KE, Ali Toure I, Mipinda JB, Diop IB, et al. Factors associated with poor adherence to medication among hypertensive patients in twelve low and middle income Sub-Saharan countries. PLoS One. 2019;14(7):e0219266.

108. Solomon Mekonnen Abebe* YBaAW. Barriers to diabetes medication adherence in North West Ethiopia. Springer Open Journal. 2014.

109. Kassaw AT, Tarekegn TB, Derbie A, Ashagrie G, Girmaw F, Mengesha A. Non-adherence to antidiabetic medications and associated factors among adult type 2 diabetes mellitus patients in Northeast Ethiopia: Institutional based cross-sectional study. 2025;15(4).

110. Mebrahtu G, M MM, Okoth Achila O, Seyoum Y, Adnoy ET, Ovberedjo M. Antihypertensive Medication Adherence and Associated Factors: A Cross-Sectional Analysis of Patients Attending a National Referral Hospital in Asmara, Eritrea. Patient Prefer Adherence. 2021;15:2619–32.

111. Denison JA, Koole O, Tsui S, Menten J, Torpey K, van Praag E, et al. Incomplete adherence among treatment-experienced adults on antiretroviral therapy in Tanzania, Uganda and Zambia. AIDS. 2015;29(3):361–71.

For peer review only

For peer review only - http://bmjopen.bmj.com/site/about/guidelines.xhtml

112. Tsige AW, Kotiso TA, Ayenew KD, Ayele SG. Pharmacist intervention to improve adherence to medication among heart failure patients at North East Ethiopia hospital. Pharmacol Res Perspect. 2024;12(3):e1199.

113. Ahmed EH, Abdel-Aziz HR, Ahmed S. Medication adherence and its influencing factors in community-dwelling older adults with chronic illnesses in a rural area. Nursing Practice Today. 2023.

114. Vreeman RC, Scanlon ML, Tu W, Slaven JE, McAteer CI, Kerr SJ, et al. Validation of a self-report adherence measurement tool among a multinational cohort of children living with HIV in Kenya, South Africa and Thailand. J Int AIDS Soc. 2019;22(5):e25304.

115. Duong M, Piroth L, Grappin M, Forte F, Peytavin G, Buisson M, et al. Evaluation of the Patient Medication Adherence Questionnaire as a tool for self-reported adherence assessment in HIV-infected patients on antiretroviral regimens. HIV Clin Trials. 2001;2(2):128–35.

116. Jose M, Rajmohan P, Sulfath TS, Varma RP, Mohan M, Jose NK, et al. Medication adherence scales in non-communicable diseases: A scoping review of design gaps, constructs and validation processes. PLoS One. 2025;20(5):e0321423.

117. Awwad O, AlMuhaissen S, Al-Nashwan A, AbuRuz S. Translation and validation of the Arabic version of the Morisky, Green and Levine (MGL) adherence scale. PLoS One. 2022;17(10):e0275778.

118. Chan AHY, Cooper V, Lycett H, Horne R. Practical Barriers to Medication Adherence: What Do Current Self- or Observer-Reported Instruments Assess? Front Pharmacol. 2020;11:572.

119. Maryem A, Younes I, Yassmine M, Morad G, Karima B, Amal K, et al. Translation, cultural adaptation and validation of the General Medication Adherence Scale (GMAS) in moroccan patients with type-2 diabetes. BMC Nurs. 2023;22(1):302.

120. Mahmoud MA, Islam MA, Ahmed M, Bashir R, Ibrahim R, Al-Nemiri S, et al. Validation of the Arabic Version of General Medication Adherence Scale (GMAS) in Sudanese Patients with Diabetes Mellitus. Risk Manag Healthc Policy. 2021;14:4235–41.

121. Merten S, Kenter E, McKenzie O, Musheke M, Ntalasha H, Martin-Hilber A. Patient-reported barriers and drivers of adherence to antiretrovirals in sub-Saharan Africa: a meta-ethnography. Trop Med Int Health. 2010;15 Suppl 1:16–33.

122. De Las Cuevas C, Penate W. Psychometric properties of the eight-item Morisky Medication Adherence Scale (MMAS-8) in a psychiatric outpatient setting. Int J Clin Health Psychol. 2015;15(2):121–9.

123. Cabral AC, Moura-Ramos M, Castel-Branco M, Fernandez-Llimos F, Figueiredo IV. Cross-cultural adaptation and validation of a European Portuguese version of the 8-item Morisky medication adherence scale. Rev Port Cardiol (Engl Ed). 2018;37(4):297–303.

124. Evangeli M, Foster C, Musiime V, Fidler S, Seeley J, Frize G, et al. Cultural Adaption, Translation, Preliminary Reliability and Validity of Key Psychological and Behavioural Measures for 18 to 25 Year-Olds Living with HIV in Uganda: A Multi-Stage Approach. AIDS Behav. 2024;28(3):924–35.

125. De AraÚJo VE, Resende CL, Melo-Souza CE, Viana-Oliveira CR, Oliveira-Filho AD. Instrumentos de autorrelato da adesão à medicação validados no Brasil: uma revisão de escopo. Journal of Hospital Pharmacy and Health Services. 2025;16(1).

For peer review only - http://bmjopen.bmj.com/site/about/guidelines.xhtml

126. Isah A, Ugochukwu EJ, Ikeanyi C, Onwujekwe O. Measuring health-related quality of life in Africa: a systematic review of validated disease-specific and generic measurement tools. Front Psychol. 2025;16:1667712.
127. Lane J, Nakambale H, Kadakia A, Dambisya Y, Stergachis A, Odoch WD. A systematic scoping review of medicine availability and affordability in Africa. BMC Health Serv Res. 2024;24(1):91.
128. Adagba KO, Aliyu AA, Ejembi CL, Olorukooba AA, Joshua IA. Comparative Study of Patients' Adherence between Hospital-based and Communitybased Treatment for Multidrug Resistant Tuberculosis (MDR-TB) in Kaduna State. Journal of Community Medicine and Primary Health Care. 2023;35(1):112–24.

For peer review only

For peer review only - http://bmjopen.bmj.com/site/about/guidelines.xhtml

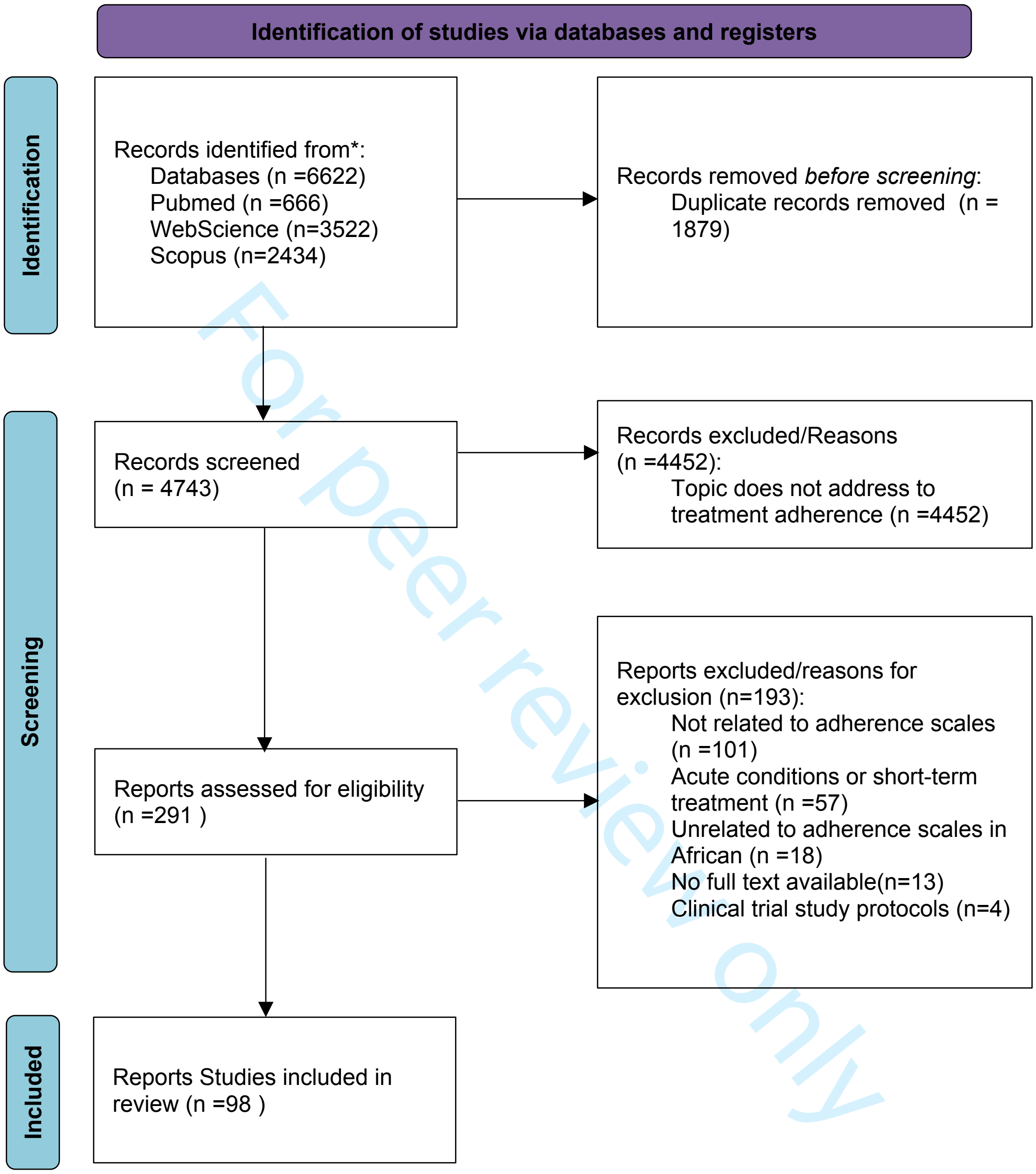

For peer review only

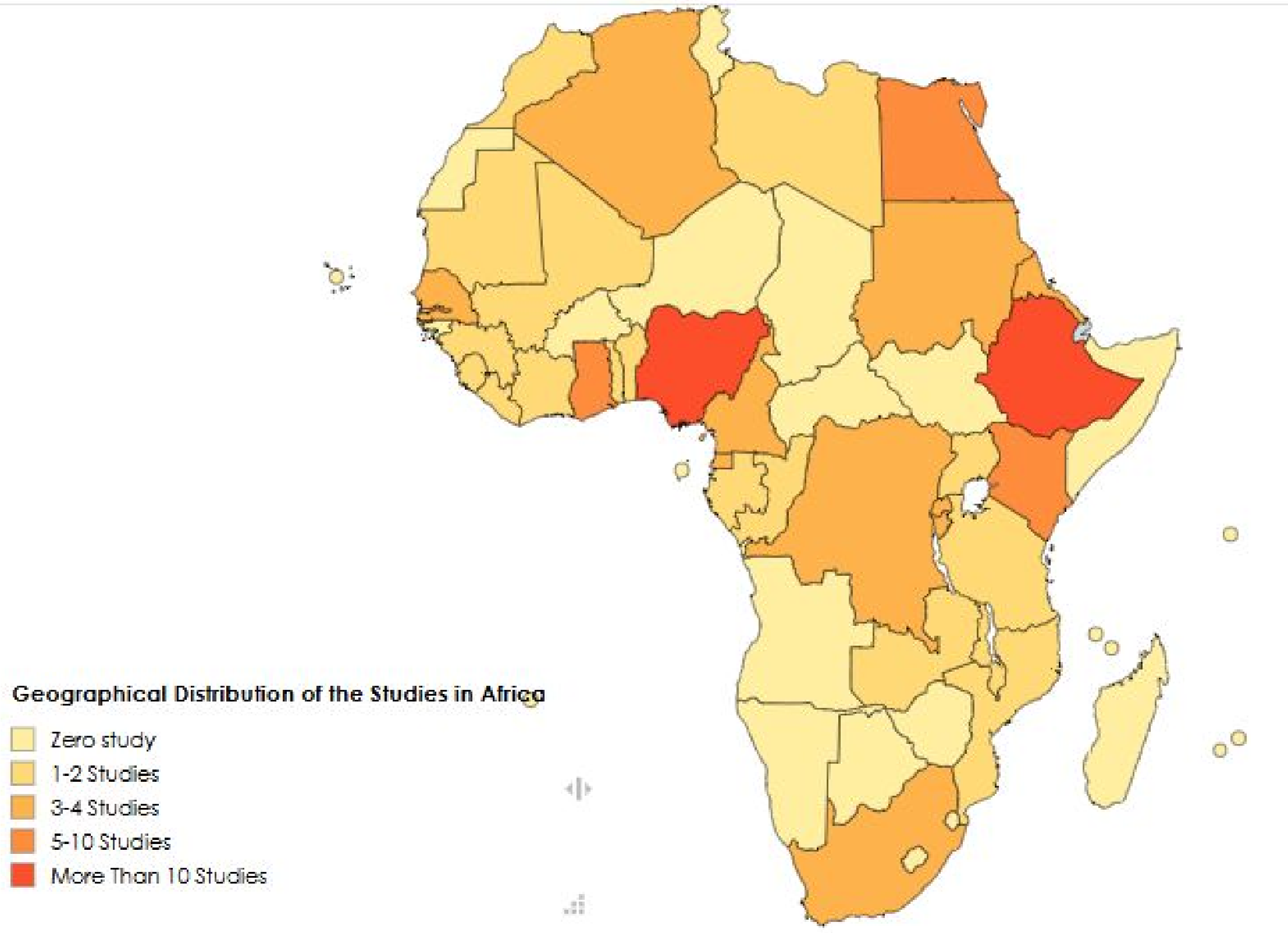
Geographical Distribution of the Studies in Africa
Zero study
1-2 Studies
3-4 Studies
5-10 Studies
More Than 10 Studies

review only

Preferred Reporting Items for Systematic reviews and Meta-Analyses extension for Scoping Reviews (PRISMA-ScR) Checklist

SECTION	ITEM	PRISMA-ScR CHECKLIST ITEM	REPORTED ON PAGE #
**TITLE**			
Title	1	Identify the report as a scoping review.	**Title Section (Page 1):** The report is explicitly identified as such: *"Mapping Medication Adherence Tools in Africa: A Scoping Review"*
**ABSTRACT**			
Structured summary	2	Provide a structured summary that includes (as applicable): background, objectives, eligibility criteria, sources of evidence, charting methods, results, and conclusions that relate to the review questions and objectives.	Page 1
**INTRODUCTION**			
Rationale	3	Describe the rationale for the review in the context of what is already known. Explain why the review questions/objectives lend themselves to a scoping review approach.	*:* **Pages 2 and 3**
Objectives	4	Provide an explicit statement of the questions and objectives being addressed with reference to their key elements (e.g., population or participants, concepts, and context) or other relevant key elements used to conceptualize the review questions and/or objectives.	Page 3
**METHODS**			
Protocol and registration	5	Indicate whether a review protocol exists; state if and where it can be accessed (e.g., a Web address); and if available, provide registration information, including the registration number.	No protocol was registered for this study
Eligibility criteria	6	Specify characteristics of the sources of evidence used as eligibility criteria (e.g., years considered, language, and publication status), and provide a rationale.	Page 5 (Eligibility Criteria)
Information sources*	7	Describe all information sources in the search (e.g., databases with dates of coverage and contact with authors to identify additional sources), as well as the date the most recent search was executed.	Page 5 (Information Sources and Search Strategy)
Search	8	Present the full electronic search strategy for at least 1 database, including any limits used, such that it could be repeated.	The literature search was carried out across three major

For peer review only - http://bmjopen.bmj.com/site/about/guidelines.xhtml

SECTION	ITEM	PRISMA-ScR CHECKLIST ITEM	REPORTED ON PAGE #
			databases: PubMed, Scopus, and Web of Science. The final search was completed on December 10, 2024. Search terms were selected based on MeSH terms and relevant keywords to ensure comprehensive coverage of the topic. The search strategy combined geographic keywords related to African countries with terms describing medication adherence (e.g., "medication compliance", "treatment nonadherence") and terms related to measurement instruments (e.g., "scale", "tool", "survey", "assessment"). Boolean operators like AND, OR, and NOT were used to combine and refine the search terms. No filters were applied regarding publication date or study type beyond the language and peer-review criteria. The complete search strategy is available in the supplementary materials. Page 5
Selection of sources of evidence†	9	State the process for selecting sources of evidence (i.e., screening and eligibility) included in the scoping review.	Page 5 (Information Sources and Search Strategy and Study Selection Process**)**.
Data charting process‡	10	Describe the methods of charting data from the included sources of evidence (e.g., calibrated forms or forms that have been tested by the team before their use, and whether data charting was done independently or in duplicate) and	Data Extraction (Page 6)<br><br>Data extraction was performed independently by two reviewers (I.M and L.M) using a structured extraction form developed for this review. Any disagreements

For peer review only

SECTION	ITEM	PRISMA-ScR CHECKLIST ITEM	REPORTED ON PAGE #
		any processes for obtaining and confirming data from investigators.	between the reviewers were resolved through discussion and consensus. The form was piloted to ensure clarity and consistency
Data items	11	List and define all variables for which data were sought and any assumptions and simplifications made.	Data Extraction (Page 6)
Critical appraisal of individual sources of evidence§	12	If done, provide a rationale for conducting a critical appraisal of included sources of evidence; describe the methods used and how this information was used in any data synthesis (if appropriate).	Page 6 (Quality Assessment).
Synthesis of results	13	Describe the methods of handling and summarizing the data that were charted.	Page 6 (Data Analysis and Synthesis)
**RESULTS**			
Selection of sources of evidence	14	Give numbers of sources of evidence screened, assessed for eligibility, and included in the review, with reasons for exclusions at each stage, ideally using a flow diagram.	Pages 7 and 8 (Results / Figure 1).
Characteristics of sources of evidence	15	For each source of evidence, present characteristics for which data were charted and provide the citations.	Pages 15-41 (Study Characteristics / Tables 1 and 2)
Critical appraisal within sources of evidence	16	If done, present data on critical appraisal of included sources of evidence (see item 12).	Not applicable
Results of individual sources of evidence	17	For each included source of evidence, present the relevant data that were charted that relate to the review questions and objectives.	**Pages 15-23 (Table1)**.
Synthesis of results	18	Summarize and/or present the charting results as they relate to the review questions and objectives.	Page 11 and 12 (Adherence Assessment Tools / Medication Adherence Rates)
**DISCUSSION**			
Summary of evidence	19	Summarize the main results (including an overview of concepts, themes, and types of evidence available), link to the review questions and objectives, and consider the relevance to key groups.	**Pages 42-45 (Discussion)**
Limitations	20	Discuss the limitations of the scoping review process.	Page 45 (Strengths and Limitations of this Study)
Conclusions	21	Provide a general interpretation of the results with respect to the review questions and objectives, as	Page 45 (Conclusion)

For peer review only - http://bmjopen.bmj.com/site/about/guidelines.xhtml

SECTION	ITEM	PRISMA-ScR CHECKLIST ITEM	REPORTED ON PAGE #
		well as potential implications and/or next steps.	
**FUNDING**			
Funding	22	Describe sources of funding for the included sources of evidence, as well as sources of funding for the scoping review. Describe the role of the funders of the scoping review.	Funding, Page 46

JBI = Joanna Briggs Institute; PRISMA-ScR = Preferred Reporting Items for Systematic reviews and Meta-Analyses extension for Scoping Reviews.

* Where *sources of evidence* (see second footnote) are compiled from, such as bibliographic databases, social media platforms, and Web sites.

† A more inclusive/heterogeneous term used to account for the different types of evidence or data sources (e.g., quantitative and/or qualitative research, expert opinion, and policy documents) that may be eligible in a scoping review as opposed to only studies. This is not to be confused with *information sources* (see first footnote).

‡ The frameworks by Arksey and O'Malley (6) and Levac and colleagues (7) and the JBI guidance (4, 5) refer to the process of data extraction in a scoping review as data charting.

§ The process of systematically examining research evidence to assess its validity, results, and relevance before using it to inform a decision. This term is used for items 12 and 19 instead of "risk of bias" (which is more applicable to systematic reviews of interventions) to include and acknowledge the various sources of evidence that may be used in a scoping review (e.g., quantitative and/or qualitative research, expert opinion, and policy document).

From: Tricco AC, Lillie E, Zarin W, O'Brien KK, Colquhoun H, Levac D, et al. PRISMA Extension for Scoping Reviews (PRISMAScR): Checklist and Explanation. Ann Intern Med. 2018;169:467–473. doi: 10.7326/M18-0850.

For peer review only

PUBMED SEARCH STRATEGY

(Africa[Title/Abstract] OR Nigeria[Title/Abstract] OR Ethiopia[Title/Abstract] OR Egypt[Title/Abstract] OR Congo[Title/Abstract] OR Tanzania[Title/Abstract] OR South Africa[Title/Abstract] OR Kenya[Title/Abstract] OR Uganda[Title/Abstract] OR Sudan[Title/Abstract] OR Algeria[Title/Abstract] OR Morocco[Title/Abstract] OR Angola[Title/Abstract] OR Ghana[Title/Abstract] OR Mozambique[Title/Abstract] OR Madagascar[Title/Abstract] OR "Côte d'Ivoire"[Title/Abstract] OR Cameroon[Title/Abstract] OR Niger[Title/Abstract] OR Mali[Title/Abstract] OR "Burkina Faso"[Title/Abstract] OR Malawi[Title/Abstract] OR Zambia[Title/Abstract] OR Chad[Title/Abstract] OR Somalia[Title/Abstract] OR Senegal[Title/Abstract] OR Zimbabwe[Title/Abstract] OR Guinea[Title/Abstract] OR Rwanda[Title/Abstract] OR Benin[Title/Abstract] OR Burundi[Title/Abstract] OR Tunisia[Title/Abstract] OR South Sudan[Title/Abstract] OR Togo[Title/Abstract] OR "Sierra Leone" OR "Libya"[Title/Abstract] OR "Congo"OR "Central African Republic"[Title/Abstract] OR Liberia[Title/Abstract] OR Mauritania[Title/Abstract] OR Eritrea[Title/Abstract] OR Gambia[Title/Abstract] OR Botswana[Title/Abstract] OR Namibia[Title/Abstract] OR Gabon[Title/Abstract] OR Lesotho [Title/Abstract] OR "Guinea-Bissau"[Title/Abstract] OR "Equatorial Guinea"[Title/Abstract] OR Mauritius[Title/Abstract] OR Eswatini[Title/Abstract] OR Djibouti[Title/Abstract] OR Comoros[Title/Abstract] OR "Cape Verde"[Title/Abstract] OR "Sao Tome and Principe"[Title/Abstract] OR Seychelles[Title/Abstract])
AND
(medication compliance[Title/Abstract]) OR (medication adherence[Title/Abstract]) OR (medication persistance[Title/Abstract]) OR (medication noncompliance[Title/Abstract]) OR (medication nonadherence[Title/Abstract]) OR (medication nonpersistance[Title/Abstract]) OR (medication non-compliance[Title/Abstract]) OR (medication non-adherence[Title/Abstract]) OR (drug compliance[Title/Abstract]) OR (drug adherence[Title/Abstract]) OR (drug persistance[Title/Abstract]) OR (drug noncompliance[Title/Abstract]) OR (drug nonadherence[Title/Abstract]) OR (drug nonpersistance[Title/Abstract]) OR (drug non-compliance[Title/Abstract]) OR (drug non-adherence[Title/Abstract]) OR (treatment compliance[Title/Abstract]) OR (treatment adherence[Title/Abstract]) OR (treatment persistance[Title/Abstract]) OR (treatment noncompliance[Title/Abstract]) OR (treatment nonadherence[Title/Abstract]) OR (treatment nonpersistance[Title/Abstract]) OR (treatment non-compliance[Title/Abstract]) OR (treatment non-adherence[Title/Abstract])
AND
(instrument∗[Title/Abstract] OR tool∗[Title/Abstract] OR measure∗[Title/Abstract] OR metric∗[Title/Abstract] OR scale∗[Title/Abstract] OR survey∗[Title/Abstract] OR assessment∗[Title/Abstract] OR screen∗[Title/Abstract]

SCOPUS SEARCH STRATEGY

“Africa” OR “Nigeria” OR “Ethiopia” OR “Egypt” OR “Congo” OR “Tanzania” OR “South Africa” OR “Kenya” OR “Uganda” OR “Sudan” OR “Algeria” OR “Morocco” OR “Angola” OR “Ghana” OR “Mozambique” OR “Madagascar” OR “Côte d'Ivoire” OR “Cameroon” OR “Niger” OR “Mali” OR “Burkina Faso” OR “Malawi” OR “Zambia” OR “Chad” OR “Somalia” OR “Senegal” OR “Zimbabwe” OR “Guinea” OR

For peer review only - http://bmjopen.bmj.com/site/about/guidelines.xhtml

“Rwanda” OR “Benin” OR “Burundi” OR “Tunisia” OR “South Sudan” OR “Togo” OR “Sierra Leone” OR “Libya” OR “Congo” OR “Central African Republic” OR “Liberia” OR “Mauritania” OR “Eritrea” OR “Gambia” OR “Botswana” OR “Namibia” OR “Gabon” OR “Lesotho” OR “Guinea-Bissau” OR “Equatorial Guinea” OR “Mauritius” OR “Eswatini” OR ”Djibouti” OR “Comoros” OR “Cape Verde” OR “Sao Tome and Principe” OR “Seychelles”

“medication compliance” OR “medication adherence” OR “medication persistance” OR “medication noncompliance” OR “medication nonadherence” OR “medication nonpersistance” OR “medication non-compliance” OR “medication non-adherence” OR “drug compliance” OR “drug adherence” OR “drug persistance” OR “drug noncompliance” OR “drug nonadherence” OR “drug nonpersistance” OR “drug non-compliance” OR “drug non-adherence” OR “treatment compliance” OR “treatment adherence” OR “treatment persistance” OR “treatment noncompliance” OR “treatment nonadherence” OR “treatment nonpersistance” OR “treatment non-compliance” OR “treatment non-adherence”

“instrument” OR “tool” OR “measure” OR “metric” OR “scale” OR “survey” OR “assessment” OR “screen”OR “instruments” OR “tools” OR “measures” OR “measurement” OR “metrics” OR “scales” OR “surveys” OR “assessments” OR “screening”

WEBSCIENCE SEARCH STRATEGY

((Africa) OR (Nigeria) OR (Ethiopia) OR (Egypt) OR (Congo) OR (Tanzania) OR (South Africa) OR (Kenya) OR (Uganda) OR (Sudan) OR (Algeria) OR (Morocco) OR (Angola) OR (Ghana) OR (Mozambique) OR (Madagascar) OR (Côte d'Ivoire) OR (Cameroon) OR (Niger) OR (Mali) OR (Burkina Faso) OR (Malawi) OR (Zambia) OR (Chad) OR (Somalia) OR (Senegal) OR (Zimbabwe) OR (Guinea) OR (Rwanda) OR (Benin) OR (Burundi) OR (Tunisia) OR (South Sudan) OR (Togo) OR (Sierra Leone) OR (Libya) OR (Congo) OR (Central African Republic) OR (Liberia) OR (Mauritania) OR (Eritrea) OR (Gambia) OR (Botswana) OR (Namibia) OR (Gabon) OR (Lesotho) OR (Guinea-Bissau) OR (Equatorial Guinea) OR (Mauritius) OR (Eswatini) OR(Djibouti) OR (Comoros) OR (Cape Verde) OR (Sao Tome and Principe) OR (Seychelles))

((medication compliance) OR (medication adherence) OR (medication persistance) OR (medication noncompliance) OR (medication nonadherence) OR (medication nonpersistance) OR (medication non-compliance) OR (medication non-adherence) OR (drug compliance) OR (drug adherence) OR (drug persistance) OR (drug noncompliance) OR (drug nonadherence) OR (drug nonpersistance) OR (drug non-compliance) OR (drug non-adherence) OR (treatment compliance) OR (treatment adherence) OR (treatment persistance) OR (treatment noncompliance) OR (treatment nonadherence) OR (treatment nonpersistance) OR (treatment non-compliance) OR (treatment non-adherence))

For peer review only - http://bmjopen.bmj.com/site/about/guidelines.xhtml

((instrument∗) OR (tool∗) OR (measure∗) OR (metric∗) OR (scale∗) OR (survey∗) OR (assessment∗) OR (screen∗))

For peer review only

For peer review only - http://bmjopen.bmj.com/site/about/guidelines.xhtml

Supplemental Table 1: Characteristics of Studies on Medication Adherence in Patients with Chronic Diseases in Africa

1

Title	Author & Year	Country	Disease	Population (n)	% of Female	Population Mean Age	Settings	Study Design	Adherence Scale Tool	Adherence Rate (%)
Evaluation of factors affecting adherence to asthma controller therapy in chest clinics in a sub-Saharan African setting: a cross-sectional study [91]	Ngahane et al.2016	Cameroon	Asthma	210	63.3	41.2	Hospital	Cross-Sectional	MMAS-8	44.8
Medication regimen complexity and its impact on medication adherence and asthma control among patients with asthma in Ethiopian referral hospitals [40]	Belachew et al.2022	Ethiopia	Asthma	396	60.1	49.4	Hospital	Cross-Sectional	MARS	24.5
Reliability of the Amharic Version of the Medication Adherence Report Scale and Beliefs about Medicines Questionnaire in Patients with Asthma in Ethiopia[49]	Gebremariam et al.2025	Ethiopia	Asthma	120	55.8	44.6	Hospital	Cross-Sectional	MARS-5	20.2
Evaluation of treatment adherence in patients with Behçet's disease: its relation to disease manifestations, patients' beliefs about medications, and quality of life [79]	Zayed et al.2019	Egypt	Behçet's Disease	67	14.9	35.0	Hospital	Cross-Sectional	CQR	69.2
Psycho-demographic and clinical predictors of medication adherence in patients with bipolar I disorder in a university hospital in Egypt [75]	Okasha et al.2020	Egypt	Bipolar I Disorder	110	32.7	36.6	Hospital	Longitudinal Prospective	MMAS-8	19.0
Breast Nurse Intervention to Improve Adherence to Endocrine Therapy Among Breast Cancer Patients in South Ethiopia [27]	Getachew et al.2022	Ethiopia	Breast Cancer	162	100.0	41.8	Hospital	Cluster Randomized	SMAQ	70.0
Validation of Amharic versions of medication adherence tools among outpatients with cardiovascular diseases: a cross-sectional study[51]	Tegegn et al.2025	Ethiopia	Cardiovascular diseases	315	45.4	54.8	Hospital	Cross-Sectional	MGLS	52.1
Medication adherence among cardiac patients in Khartoum State, Sudan: a cross-sectional study [89]	Awad et al.2017	Sudan	Cardiovascular Diseases ((hypertension, dyslipidaemia, ischaemic heart disease, chronic heart failure, Arrhythmia, cardiac valve replacement, rheumatic heart disease,	386	57.0		Hospital	Cross-Sectional	MMAS-8	49.0

			cerebrovascular disease)							
A cross-sectional study on the assessment of adherence to cardiovascular medications in Sudan heart center[93]	Mahmoud et al.2025	Sudan	Cardiovascular Diseases (Hypertension, Ischemic Heart Disease, and Heart Failure)	353	40.5	58.7	Hospital	Cross-Sectional	MGLS-4	36.5
Medication adherence and its influencing factors in community-dwelling older adults with chronic illnesses in a rural area [113]	Ahmed et al.2023	Egypt	Chronic Illnesses (hypertension, cardiac disease, renal disease, arthritis, diabetes, neurological, hepatic, osteoporosis, respiratory, gastrointestinal, thyroid)	310	52.6	69.7	Hospital	Cross-Sectional	GMAS	50.6
Evaluation of medication adherence and predictors of sub-optimal adherence among pre-dialysis patients with chronic kidney disease [56]	Okoro et al.2020	Nigeria	Chronic Kidney Disease	220	61.4	52.7	Hospital	Prospective	MMAS	48.6
Impact of pharmacist intervention on antidepressant medication adherence and disease severity in patients with major depressive disorder in fragile north-east Nigeria [57]	Yusuf et al.2021	Nigeria	Depressive Disorders	101	80.2		Hospital	Prospective Interventional	MARS	9.5
Magnitude and factors associated with treatment non-adherence among patients with depressive disorders at St. Amanuel Mental Specialized Hospital, Addis Ababa, Ethiopia, 2019: A cross sectional study [31]	Alemayehu et al. 2022	Ethiopia	Depressive Disorders	415	53.0	36.0	Hospital	Cross-Sectional	MARS	74.0
Self-Reported Adverse Drug Reactions, Medication Adherence, and Clinical Outcomes among Major Depressive Disorder Patients in Ethiopia: A Prospective Hospital Based Study [42]	Abegaz et al. 2017	Ethiopia	Depressive Disorders	217	43.8	30.9	Hospital	Cross-Sectional	MMAS-8	10.6
Effect of patients beliefs about medications on adherence to drugs in diabetic patients attending family medicine outpatient clinic in Ismailia, Egypt [76]	Salama & Saudi. 2020	Egypt	Diabetes Mellitus	82	85.4	18.6	Hospital	Cross-Sectional	MMAS-8	45.1
Medication belief as correlate of medication adherence among patients with diabetes in Edo State, Nigeria [66]	Olorunfemi and Ojewo	Nigeria	Diabetes Mellitus	180	55.6	57.2	Hospital	Descriptive Correlational	BMQ and MMAS	5.6

For peer review only

	le.2019									
Adherence to Treatment and Factors Affecting Adherence of Epileptic Patients at Yirgalem General Hospital, Southern Ethiopia: A Prospective Cross-Sectional Study [18]	Hasiso and Desse.2016	Ethiopia	Epilepsy	194	43.8	33.6	Hospital	Cross-Sectional	MMAS-8	32.0
Antiepileptic Drug Adherence and Its Associated Factors among Epilepsy Patients on Follow-ups at Amanuel Mental Specialized Hospital, Ethiopia [19]	Shum et et al.2022	Ethiopia	Epilepsy	439	35.3	16.4	Hospital	Cross-Sectional	MARS-5	16.4
Antiepileptic drug adherence in children in southern Ethiopia: A cross sectional study [20]	Dima and Shibesh. 2022	Ethiopia	Epilepsy	192	33.3	6.9	Hospital	Cross-Sectional	MMAS-8	65.0
Antiepileptic Drug Nonadherence and Its Predictors among People with Epilepsy [21]	Getnet et al.2016	Ethiopia	Epilepsy	450	41.3	27.0	Hospital	Cross-Sectional	MMAS	62.2
Assessment of Patients' Adherence to Antiepileptic Medications at Dessie Referral Hospital, Chronic Follow-Up, South Wollo, Amhara Region, North East Ethiopia [25]	Kassahun et al.2018	Ethiopia	Epilepsy	88	37.5		Hospital	Cross-Sectional	MMAS	65.9
Beliefs about medication, medication adherence and seizure control amongadult epilepsy patients in Kimberley, South Africa [96]	Egenas et al.2015	South Africa	Epilepsy	197	39.1	40.0	Hospital	Descriptive Observational	MMAS-8	54.6
Factors affecting adherence to antiepileptic medications among Sudanese individuals with epilepsy: A cross-sectional survey [90]	Elsayed et al.2019	Sudan	Epilepsy	96	68.8	29.0	Hospital	Cross-Sectional	MMAS-4	4.0
Associations Between Personality Traits and Adherence to Treatment in Patients with Primary Open Angle Glaucoma in an African Population [84]	Antir et al.2021	Ghana	Glaucoma	400	52.8	56.2	Hospital	Cross-Sectional	MARS-5	60.8
Drug utilization, medication adherence and treatment satisfaction amongst glaucoma patients receiving care at secondary health facilities [55]	Bello et al.2023	Nigeria	Glaucoma	311	59.2	63.8	Hospital	Cross-Sectional	MMAS	46.3
Treatment adherence and associated factors among glaucoma patients attending Ophthalmic units of referral hospitals in North West Ethiopia, 2019 [45]	Birhanie et al.2022	Ethiopia	Glaucoma	382	30.9	57.7	Hospital	Cross-Sectional	MMAS-8	49.5

Medication Adherence and Its Determinants Among Adults With Heart Failure: A Study in Selected Hospitals of North Shewa Zone, Oromia, Ethiopia (2024)-A Multicenter Institutional-Based Cross-Sectional Study[46]	Mohammed et al.2026	Ethiopia	Heart Failure	327	50.5	43.0	Hospital	Cross-Sectional	MMAS-8	44.7
Medication Nonadherence and Associated Factors among Heart Failure Patients at University of Gondar Comprehensive Specialized Hospital, Northwest Ethiopia [39]	Seid et al.2023	Ethiopia	Heart Failure	245	66.5	47.8	Hospital	Cross-Sectional	MARS-5	76.3
Pharmacist intervention to improve adherence to medication among heart failure patients at North East Ethiopia hospital [112]	Tsige et al.2024	Ethiopia	Heart Failure	412	53.6	44.7	Hospital	Pre-Post Interventional	MMAS-4	36.4
Factors Influencing Adherence to Antiretroviral Therapy Among Adolescents at Busia County Referral Hospital[88]	Mayamba et al.2025	Kenya	HIV	154	52.6	14.1	Hospital	Cross-Sectional	Self-report	73.4
Cultural adaptation and psychometric validation of the Amharic adherence barriers questionnaire for HIV patients on antiretroviral therapy in Ethiopia[50]	TAregn et al.2026	Ethiopia	HIV	200	59.5	42.1	Hospital	Cross-Sectional	MMAS-8	61.5
Clinical Utility of Pharmacy-Based Adherence Measurement in Predicting Virologic Outcomes in an Adult HIV-Infected Cohort in Jos, North Central Nigeria [53]	Abah et al.2016	Nigeria	HIV	926	66.0	34.0	Hospital	Retrospective	Drug-Refill Visits	57.5
Factors contributing to antiretroviral drug adherence among adults living with HIV or AIDS in a Kenyan rural community [80]	Kioko et al.2017	Kenya	HIV	301	62.8	40.4	Hospital	Cross-Sectional	PMAQ	86.0
Food Insecurity Is Associated with Increased Risk of NonAdherence to Antiretroviral Therapy among HIV-Infected Adults in the Democratic Republic of Congo: A CrossSectional Study [99]	Musumari et al.2014	Democratic Republic of Congo	HIV	898	72.2	44.0	Hospital	Cross-Sectional	Self-reported Adherence	79.1
Incomplete adherence among treatment-experienced adults on antiretroviral therapy in Tanzania, Uganda and Zambia [111]	Denison et al.2015	Tanzania, Uganda And Zambia	HIV	4489	68.0	40.0	Hospital	Cross-Sectional	Self-report, provider report and pharmacy refill	85.5
Measuring adherence to antiretroviral therapy in children and adolescents in western Kenya [82]	Vreeman et	Kenya	HIV	210	100.0	8.2	Hospital	Prospective Cohort	Electronic Medicati	96.3

	al.2014								on Monitoring Using Mems	
Medication adherence in HIV-positive pregnant women on antiretroviral therapy attending antenatal clinics in ado metropolis, south-west nigeria: A multicentre study [63]	Aduloju et al.2020	Nigeria	HIV	170	100.0	29.8	Hospital	Cross-Sectional	MMAS-8	75.3
Mood and anxiety disorders adversely influence medication adherence to antiretroviral therapy among people living with HIV/AIDS in Nigeria [68]	Omonefe Seb-Akahomen et al.2018	Nigeria	HIV	410	75.9	40.4	Hospital	Cross-Sectional	MMAS-8	63.2
Prevalence and determinants of adherence to antiretroviral treatment among HIV patients on first-line regimen: a cross-sectional study in Dakar, Senegal [103]	Mbengue et al.2019	Senegal	HIV	150	75.3	43.0	Hospital	Cross-Sectional	SMAQ and Self-reporting tool	30.6
Socio-demographic factors associated with medication adherence among People Living with HIV in the Kumasi Metropolis, Ghana [85]	Adu et al.2022	Ghana	HIV	420	80.0	45.0	Hospital	Cross-Sectional	MMAS-8	53.1
The role of health facility and individual level characteristics on medication adherence among PLHIV on second-line antiretroviral therapy in Northeast Ethiopia: use of multi-level model [44]	Wedajo et al.2022	Ethiopia	HIV	714	56.0		Hospital	Cross-Sectional	SMAQ	69.5
Using a Self-Administered Electronic Adherence Questionnaire to Identify Poor Adherence Amongst Adolescents and Young Adults on First-Line Antiretroviral Therapy in Johannesburg, South Africa [95]	Hirasen et al. 2020	South Africa	HIV	278	78.8	31.0	Hospital	Cross-Sectional	Self-reported Adherence tool and SMAQ	26.7
Adherence to hypertension management at a rural hospital in Limpopo: A cross-sectional study[94].	Murendeni et al.2025	South Africa	Hypertension	258	71.3	58.6	Hospital	Cross-Sectional	MMAS-8	39.5
Adherence to antihypertensive medication and its correlates among individuals with hypertension in a semi-urban community of Southern Nigeria [52]	Okwuonu et al.2015	Nigeria	Hypertension	252	43.3	56.6	Hospital	Cross-Sectional	MMAS-8	31.8

For peer review only - http://bmjopen.bmj.com/site/about/guidelines.xhtml

Adherence to Antihypertensive Treatment and Associated Factors in Central Ethiopia [16]	Tsadik, Berhane,and Worku.2020	Ethiopia	Hypertension	989	52.7	57.6	Hospital	Cross-Sectional	MMAS-8	36.0
Antihypertensive medication adherence and associated factors among adult hypertensive patients at Jimma University Specialized Hospital, southwest Ethiopia [22]	Asgedom et al.2018	Ethiopia	Hypertension	280	46.8	57.7	Hospital	Cross-Sectional	MMAS-8	61.8
Antihypertensive Medication Adherence and Associated Factors: A Cross-Sectional Analysis of Patients Attending a National Referral Hospital in Asmara, Eritrea [110]	Mebrahtu et al.2021	Eritrea	Hypertension	335	66.3	59.7	Hospital	Cross-Sectional	MARS	27.2
Factors associated with poor adherence to medication among hypertensive patients in twelve low and middle income Sub-Saharan countries [107]	Terline et al.2019	Benin, Cameroon, Congo, Democratic Republic of Congo, Gabon, Guinea, Costa de Marfim, Mauritania, Mozambique, Niger, Senegal and Togo	Hypertension	2198	88.2	57.7	Hospital	Cross-Sectional	Medication Adherence Scale-8 (MAS-8)	30.8
Family support and medication adherence among residents with hypertension in informal settlements of Nairobi, Kenya: a mixed-method study [81]	Xiong et al.2023	Kenya	Hypertension	93	65.6	57.0	Urban Settlement	Mixed-Methods	MMAS-8	43.0
General self-efficacy and hypertension treatment adherence in Algerian private clinical settings [98]	Said Kara.2022	Algeria	Hypertension	216	52.8	25.3	Hospital	Cross-Sectional	Treatment Adherence Scale[98]	
Impact of adverse drug events and treatment satisfaction on patient adherence with antihypertensive	Berhe et	Ethiopia	Hypertension	925	61.6	57.0	Hospital	Cross-Sectional	MMAS-8	21.0

peer review only

medication – a study in ambulatory patients [29]	al.2017									
Medication adherence and its associated factors among hypertensive patients attending the Debre Tabor General Hospital, northwest Ethiopia [35]	Teshome et al.2017	Ethiopia	Hypertension	337	51.6	58.3	Hospital	Cross-Sectional	MGLS	75.1
Medication Adherence and Patient Satisfaction among Hypertensive Patients Attending Outpatient Clinic in Lagos University Teaching Hospital, Nigeria [61]	Oluwole et al.2019	Nigeria	Hypertension	500	56.8	58.9	Hospital	Cross-Sectional	MMAS-8	90.2
Medication Adherence and Perceived Family Support Among Elderly Patients with Hypertension Attending a Specialty Clinic in Lagos, Nigeria [62]	Olaniran et al.2023	Nigeria	Hypertension	293	73.0	69.3	Hospital	Cross-Sectional	MMAS-8	14.3
Relationship between Cost of Care and Medication Adherence among Hypertensive Patients Attending a Tertiary Health Facility in Ekiti State, Nigeria [71]	Ipinnimo et al.2022	Nigeria	Hypertension	138	54.4	52.6	Hospital	Cross-Sectional	MMAS-8	34.1
Treatment adherence and blood pressure outcome among hypertensive out-patients in two tertiary hospitals in Sokoto, Northwestern Nigeria [73]	Adisa et al.2018	Nigeria	Hypertension	605	59.2	54.5	Hospital	Cross-Sectional	MMAS-9	8.9
Treatment satisfaction and medication adherence among hypertensive patients in a teaching hospital in Ekiti State, Nigeria [74]	Akpor and Olowolaju.2022	Nigeria	Hypertension	270	63.0		Hospital	Cross-Sectional	MMAS-8	9.3
Validity and Reliability of a Self-Reported Measure of Antihypertensive Medication Adherence in Uganda [105]	Okello et al.2016	Uganda	Hypertension	329	69.3	55.0	Hospital	Cross-Sectional	MMAS-8	3.0
Depression and medication-adherence in patients with hypertension attending a tertiary health facility in South-West Nigeria [54]	Okunrinboye et al.2019	Nigeria	Hypertension And Depression	400	62.0	59.6	Hospital	Cross-Sectional	MMAS-8	96.8
Magnitude and associated factors of poor medication adherence among diabetic and hypertensive patients visiting public health facilities in Ethiopia during the COVID-19 pandemic [30]	Shimels et al.2021	Ethiopia	Hypertension and Diabetes Mellitus	409	56.0	56.5	Hospital	Cross-Sectional	MMAS-8	28.0
Medication non-adherence and its associated factors among kidney transplant patients in a large teaching hospital in Ethiopia [38]	Dereje et al.2024	Ethiopia	Kidney Transplant	270	28.5	37.0	Hospital	Cross-Sectional	SMAQ	81.9

For peer review only

Medication adherence and associated factors among psychiatry patients at Asella Referral and Teaching Hospital in Oromia, Ethiopia: Institution based cross sectional study [32]	Gudeta et al.2023	Ethiopia	Mental Illness (Major depressive disorder, schizophrenia, bipolar disorders, Brief psychotic disorder and schizophreniform disorder)	395	58.0	33.0	Hospital	Cross-Sectional	MARS	62.3
Medication compliance behavior in psychiatric out-patients with psychoactive substance use comorbidity in a Nigerian tertiary hospital [67]	Okpataku et al.2015	Nigeria	Psychiatric Out-Patient	208	47.1	36.7	Hospital	Cross-Sectional	Self-Administered Medication Adherence Scale	59.0
Medication adherence among Nigerian patients with rheumatoid arthritis: a two instruments survey [58]	Ubaka et al.2021	Nigeria	Rheumatoid Arthritis	169	62.7		Hospital	Cross-Sectional	MARS-5	11.8
Status of rheumatoid arthritis practice and treatment in Sudan [10]	Ali et al. 2023	Sudan	Rheumatoid Arthritis	90	93.3	55.0	Hospital	Cross-Sectional Descriptive	MMAS-8	0.0
Impact of sociodemographic factors and depression on medication adherence in schizophrenia: Cross sectional study in Morocco [106]	Doufik et al.2024	Morocco	Schizophrenia	305	21,3	35.5	Hospital	Cross-Sectional	MARS	78.7
Antipsychotic medication non-adherence among schizophrenia patients in Central Ethiopia [23]	Tareke et al. 2018	Ethiopia	Schizophrenia	412	30.3	35.3	Hospital	Cross-Sectional	MARS	59.0
Antipsychotic medication non-adherence and factors associated among patients with schizophrenia in eastern Ethiopia [24]	Mohammed et al.2024	Ethiopia	Schizophrenia	893	18.4	34.2	Hospital	Cross-Sectional	MARS	59.0
Medication adherence among Nigerians with schizophrenia: correlation between clinico-demographic factors and quality of life [59]	Ogunnubi et al.2017	Nigeria	Schizophrenia	160	53.8	38.5	Hospital	Cross-Sectional	MMAS	45.0
Quality of life and its association with current substance use, medication nonadherence and clinical factors of people with schizophrenia in Southwest Ethiopia: a hospital-based cross-sectional study [41]	Desalegn et al. 2020	Ethiopia	Schizophrenia	351	31.1	33.0	Hospital	Cross-Sectional	MMAS-4	68.4

Prevalence and predictors of sub-optimal medication adherence among patients with severe mental illnesses in a tertiary psychiatric facility in Maiduguri, North-eastern Nigeria [70]	Ibrahim et al.2015	Nigeria	Severe Mental Illnesses (schizophrenia, bipolar disorder or severe depression)	370	43.5	35.0	Hospital	Cross-Sectional	MMAS-8	44.3
Barriers to diabetes medication adherence in North West Ethiopia [108]	Abebe et al.2014	Ethiopia	Type 1 and Type 2 Diabetes Mellitus	391	52.9	50.4	Hospital	Cross-Sectional	MMAS-8	71.2
Drug therapy problems, medication adherence and treatment satisfaction among diabetic patients on follow-up care at Tikur Anbessa Specialized Hospital, Addis Ababa, Ethiopia Gebre Teklemariam D [28]	Demoz et al.2019	Ethiopia	Type 1 and Type 2 Diabetes Mellitus	418	52.9	53.0	Hospital	Cross-Sectional	MMAS-8	45.7
Health literacy, medication belief, and demographic factors as correlates of medication adherence in person with diabetes mellitus [11]	Olorunfemi and Olaolorunpo. 2023	Benin	Type 1 and Type 2 Diabetes Mellitus	180	55.6	57.2	Hospital	Descriptive Correlational	MARS and BMQ	5.6
Medication adherence and direct treatment cost among diabetes patients attending a tertiary healthcare facility in Ogbomosho, Nigeria [60]	Fadare et al.2015	Nigeria	Type 1 and Type 2 Diabetes Mellitus	129	60.5	61.4	Hospital	Cross-Sectional	MMAS-8	40.6
Medication adherence and its associated factors among diabetic patients at Zewditu Memorial Hospital, Addis Ababa, Ethiopia [34]	Muhammed et al.2017	Ethiopia	Type 1 and Type 2 Diabetes Mellitus	146	54.1	46.5	Hospital	Cross-Sectional	MMAS-8	54.8
Medication non-adherence and associated factors among diabetes patients in Felege Hiwot Referral Hospital, Bahir Dar city administration, Northwest Ethiopia [37]	Teshager and Abate. 2019	Ethiopia	Type 1 and Type 2 Diabetes Mellitus	416	41.8	45.4	Hospital	Cross-Sectional	MMAS-8	31.2
Non-adherence to antidiabetic medications and associated factors among adult type 2 diabetes mellitus patients in Northeast Ethiopia: institutional based cross-sectional study[47]	Kassaw et al.2025	Ethiopia	Type 2 Diabetes Mellitus	401	42.4	53.6	Hospital	Cross-Sectional	MMAS-8	68.8
Machine learning applications to classify and monitor medication adherence in patients with type 2 diabetes in Ethiopia[48]	Kassaw et al.2025	Ethiopia	Type 2 Diabetes Mellitus	595	45.4	55.4	Hospital	Cross-Sectional	MMAS-8	55.3
Adherence to antidiabetic medication and factors associated with non-adherence among patients with type-2	Aminde et al.2019	Cameroon	Type 2 Diabetes Mellitus	195	69.7	60.5	Hospital	Cross-Sectional	MCQ	45.6

diabetes mellitus in two regional hospitals in Cameroon [92]										
Adherence to Oral Antidiabetic Medications among Type 2 Diabetic (T2DM) Patients in Chronic Ambulatory Wards of Hiwot Fana Specialized University Hospital, Harar, Eastern Ethiopia: A Cross Sectional Study [17]	Jemal et al.2017	Ethiopia	Type 2 Diabetes Mellitus	196	45.4	61.1	Hospital	Cross-Sectional	MMAS-4	70.4
Association of treatment satisfaction and physician trust with glycemic control among primary care patients with type 2 diabetes in Egypt [78]	Ahmed et al.2024	Egypt	Type 2 Diabetes Mellitus	319	57.1	59.7	Hospital	Cross-Sectional	MMAS-8 and MKQ	79.9
Factors associated with medication non-adherence in patients with type 2 diabetes mellitus: The first Algerian survey [97]	Achouri et al.2019	Algeria	Type 2 Diabetes Mellitus	403	0	60.0	Hospital	Cross-Sectional	MMAS-8	68.7
Illness perceptions of Libyans with T2DM and their influence on medication adherence: a study in a diabetes center in Tripoli [100]	Ashur et al.2015	Libya	Type 2 Diabetes Mellitus	523	58.9	54.4	Hospital	Cross-Sectional	MMAS-8	63.9
Ilness perception, medication adherence and glycemic control among primary health-care patients with type 2 diabetes mellitus at Port Said City, Egypt [77]	Saudi et al.2022	Egypt	Type 2 Diabetes Mellitus	265	58.5	58.8	Hospital	Cross-Sectional	MMAS-8	32.5
Low medication adherence and its associated factors among patients with type 2 diabetes mellitus attending Amana Hospital in Dar es Salaam, Tanzania: a cross-sectional study [101]	Doyaa et al.2024	Tanzania	Type 2 Diabetes Mellitus	248	70.2	59.8	Hospital	Cross-Sectional	MMAS-8	26.2
Medication Adherence and Contributing Factors Among Type 2 Diabetes Patients at Adama Hospital Medical College in Eastern Ethiopia [33]	Yosef et al.2023	Ethiopia	Type 2 Diabetes Mellitus	245	44.9	48.6	Hospital	Cross-Sectional	MARS-5	29.4
Medication adherence and factors associated with poor adherence among type 2 diabetes mellitus patients on follow-up at Kenyatta National Hospital, Kenya [83]	Waari et al.2018	Kenya	Type 2 Diabetes Mellitus	290	67.6	56.6	Hospital	Cross-Sectional	MMAS-8	45.5
Medication adherence and its impact on glycemic control in type 2 diabetes mellitus patients with comorbidity: A multicenter cross-sectional study in Northwest Ethiopia [36]	Sendekie et al.2022	Ethiopia	Type 2 Diabetes Mellitus	403	45.2	45.2	Hospital	Cross-Sectional	GMAS	23.1
Medication adherence and self-care behaviours among patients with type 2 diabetes mellitus in Ghana [86]	Afaya et al.2020	Ghana	Type 2 Diabetes Mellitus	330	68.2	57.5	Hospital	Cross-Sectional	MAQ	84.5

Medication Adherence in Type 2 Diabetes Patients in Nigeria [64]	Jackson et al.2015	Nigeria	Type 2 Diabetes Mellitus	303	56.5		Hospital	Cross-Sectional	MMAS-8	19.8
Medication adherence in type 2 diabetes patients: Study of patients in alimosho general hospital, Igando, Lagos, Nigeria [65]	Olufunsho and Osuolale.2015	Nigeria	Type 2 Diabetes Mellitus	152	72.4		Hospital	Descriptive (Retrospective & Prospective)	MARS	86.8
Non-adherence to medication and associated factors among type 2 diabetes patients at Clinique Medicale Fraternite, Rwanda: a cross-sectional study [102]	Murwanashyaka et al.2022	Rwanda	Type 2 Diabetes Mellitus	200	53.0		Hospital	Cross-Sectional	MMAS-4	46.5
The Association between Diabetes-Related Distress and Medication Adherence in Adult Patients with Type 2 Diabetes Mellitus: A Cross-Sectional Study [87]	Kretchy et al.2020	Ghana	Type 2 Diabetes Mellitus	188	72.3	59.3	Hospital	Cross-Sectional	MARS	34.5
The impact of pharmacist-led medication therapy management on medication adherence in patients with type 2 diabetes mellitus: a randomized controlled study [43]	ERKU et al.2017	Ethiopia	Type 2 Diabetes Mellitus	107	43.0	60.6	Hospital	Randomized Controlled	MMAS-8	9.2
The Influence of National Health Insurance on Medication Adherence Among Outpatient Type 2 Diabetics in Southwest Nigeria [72]	Ajibola et al.2018	Nigeria	Type 2 Diabetes Mellitus	110	50.9		Hospital	Cross-Sectional	MMAS-8	10.9
Validation and psychometric properties of the 8-item Morisky Medication Adherence Scale (MMAS-8) in Type 2 diabetes patients in sub-Saharan Africa [104]	Tandon et al.2015	Togo	Type 2 Diabetes Mellitus	308	72.7	57.5	Hospital	Cross-Sectional	MMAS-8	18.8

MMAS-8 (Morisky Medication Adherence Scale – 8 items), SMAQ (Simplified Medication Adherence Questionnaire), MARS (Medication Adherence Report Scale), MCQ (Medication Compliance Questionnaire), PMAQ (Patient Medication Adherence Questionnaire), CQR (Compliance Questionnaire on Rheumatology), GMAS (General Medication Adherence Scale), MGLS (Morisky-Green-Levine Scale), MAQ (Medication Adherence Questionnaire), MAS (Medication Adherence Scale), MKQ (Medication Knowledge Questionnaire), BMQ (Brief medication Questionnaire).

1

Supplemental Table 2: Methodological Characteristics of Medication Adherence Assessment Tools Used in African Studies

Study	Tool	Validated locally	Translation/Cultural adaptation	Administration mode	Disease-specific tool	Additional adherence method
Evaluation of treatment adherence in patients with Behçet's disease: its relation to disease manifestations, patients' beliefs about medications, and quality of life [79]	CQR	No	Arabic translation	Interview	Yes	None
Clinical Utility of Pharmacy-Based Adherence Measurement in Predicting Virologic Outcomes in an Adult HIV-Infected Cohort in Jos, North Central Nigeria[53]	Drug-Refill Visits	Clinical utility and predictive validity of pharmacy refill metrics in a local Nigerian public care facility	Not applicable	Electronic record review	Yes	None
Measuring adherence to antiretroviral therapy in children and adolescents in western Kenya[82]	Electronic Medication Monitoring Using Mems	No	Kiswahili or English translation	Interview	Yes	Electronic Dose Monitoring and Plasma Drug Concentrations
Medication adherence and its influencing factors in community-dwelling older adults with chronic illnesses in a rural area [113]	GMAS	No	Arabic translation	Interview	No	None
Medication adherence and its impact on glycemic control in type 2 diabetes mellitus patients with comorbidity: A multicenter cross-sectional study in Northwest Ethiopia[36]	GMAS	No	Afan Oromo and Amharic	Interview	Yes	None
Medication adherence and self-care behaviours among patients with type 2 diabetes mellitus in Ghana[86]	MAQ	No	Akan	Interview	Yes	None
Medication regimen complexity and its impact on medication adherence and asthma control among patients with asthma in Ethiopian referral hospitals [40]	MARS	No	Not specified	Interview	Yes	None
Impact of pharmacist intervention on antidepressant medication adherence and disease severity in patients with major depressive disorder in fragile north-east Nigeria [57]	MARS	No	Not specified	Interview	No	None
Antihypertensive Medication Adherence and Associated Factors: A Cross-Sectional Analysis of	MARS	No	Not specified	Interview	No	None

For peer review only

Patients Attending a National Referral Hospital in Asmara, Eritrea [110]						
Medication adherence and associated factors among psychiatry patients at Asella Referral and Teaching Hospital in Oromia, Ethiopia: Institution based cross sectional study[32]	MARS	Yes	Amharic or Afaan Oromo	Interview	No	None
Antipsychotic medication non-adherence among schizophrenia patients in Central Ethiopia[23]	MARS	Yes	Not specified	Interview	No	None
Antipsychotic medication non-adherence and factors associated among patients with schizophrenia in eastern Ethiopia [24]	MARS	No	Afan Oromo and Amharic	Interview	No	None
Health literacy, medication belief, and demographic factors as correlates of medication adherence in person with diabetes mellitus [11]	MARS	No	Not specified	Interview	No	BMQ
Medication adherence in type 2 diabetes patients: Study of patients in alimosho general hospital, Igando, Lagos, Nigeria[65]	MARS	No	Not specified	Interview	Yes	None
The Association between Diabetes-Related Distress and Medication Adherence in Adult Patients with Type 2 Diabetes Mellitus: A Cross-Sectional Study [87]	MARS	No	Not specified	Interview	Yes	None
Impact of sociodemographic factors and depression on medication adherence in schizophrenia: Cross sectional study in Morocco[106]	MARS	No	Translated from English to Moroccan Arabic dialect	Interview	No	None
Antiepileptic Drug Adherence and Its Associated Factors among Epilepsy Patients on Follow-ups at Amanuel Mental Specialized Hospital, Ethiopia[19]	MARS-5	No	Translated into Amharic	Interview	No	None
Associations Between Personality Traits and Adherence to Treatment in Patients with Primary Open Angle Glaucoma in an African Population [84]	MARS-5	No	No	Interview	No	None
Medication Nonadherence and Associated Factors among Heart Failure Patients at University of Gondar Comprehensive Specialized Hospital, Northwest Ethiopia[39]	MARS-5	Yes	Translated into Amharic	Interview	No	None

For peer review only - http://bmjopen.bmj.com/site/about/guidelines.xhtml

Medication adherence among Nigerian patients with rheumatoid arthritis: a two instruments survey[58]	MARS-5	No	Not specified	Interview	No	None
Medication Adherence and Contributing Factors Among Type 2 Diabetes Patients at Adama Hospital Medical College in Eastern Ethiopia[33]	MARS-5	No	Not specified	Interview	No	None
Reliability of the Amharic Version of the Medication Adherence Report Scale and Beliefs about Medicines Questionnaire in Patients with Asthma in Ethiopia[49]	MARS-5	Yes	English to the local language (Amharic)	Interview	No	None
Adherence to antidiabetic medication and factors associated with non-adherence among patients with type-2 diabetes mellitus in two regional hospitals in Cameroon[92]	MCQ	No	Not specified	Self-Administered	No	None
Medication adherence and its associated factors among hypertensive patients attending the Debre Tabor General Hospital, northwest Ethiopia[35]	MGLS	No	Not specified	Interview	No	None
A cross-sectional study on the assessment of adherence to cardiovascular medications in Sudan heart center[93]	MGLS	Yes	English to the local language (Sudanese)	Interview	No	None
Validation of Amharic versions of medication adherence tools among outpatients with cardiovascular diseases: a cross-sectional study[51]	MGLS	Yes	English to the local language (Amharic)	Interview	No	None
Evaluation of medication adherence and predictors of sub-optimal adherence among pre-dialysis patients with chronic kidney disease [56]	MMAS	No	Not specified	Self-reported questionnaire or Interview	No	None
Medication belief as correlate of medication adherence among patients with diabetes in Edo State, Nigeria[66]	MMAS	No	Not specified	Questionnaires	No	BMQ
Antiepileptic Drug Nonadherence and Its Predictors among People with Epilepsy [21]	MMAS	No	Translated into Amharic	Interview	No	None
Assessment of Patients' Adherence to Antiepileptic Medications at Dessie Referral Hospital, Chronic Follow-Up, South Wollo, Amhara Region, North East Ethiopia[25]	MMAS	No	Translated into Amharic	Interview	No	None

Medication adherence among Nigerians with schizophrenia: correlation between clinico-demographic factors and quality of life [59]	MMAS	No	Not specified	Interview	No	None
Factors affecting adherence to antiepileptic medications among Sudanese individuals with epilepsy: A cross-sectional survey[90]	MMAS-4	No	Arabic translation	Interview	No	None
Drug utilization, medication adherence and treatment satisfaction amongst glaucoma patients receiving care at secondary health facilities[55]	MMAS-4	No	No	Interview	No	None
Pharmacist intervention to improve adherence to medication among heart failure patients at North East Ethiopia hospital[112]	MMAS-4	Yes	Translated into Amharic	Interview	No	None
Quality of life and its association with current substance use, medication nonadherence and clinical factors of people with schizophrenia in Southwest Ethiopia: a hospital-based cross-sectional study[41]	MMAS-4	No	Afaan Oromo and Amharic	Interview	No	None
Adherence to Oral Antidiabetic Medications among Type 2 Diabetic (T2DM) Patients in Chronic Ambulatory Wards of Hiwot Fana Specialized University Hospital, Harar, Eastern Ethiopia: A Cross Sectional Study[17]	MMAS-4	No	Afan Oromo and Amharic	Interview	No	None
Non-adherence to medication and associated factors among type 2 diabetes patients at Clinique Medicale Fraternite, Rwanda: a cross-sectional study[102]	MMAS-4	No	Not specified	Interview	No	None
Evaluation of factors affecting adherence to asthma controller therapy in chest clinics in a sub-Saharan African setting: a cross-sectional study [91]	MMAS-8	No	Not specified	Interview	No	None
Psycho-demographic and clinical predictors of medication adherence in patients with bipolar I disorder in a university hospital in Egypt [75]	MMAS-8	Yes	Arabic translation	Interview	No	None
Medication adherence among cardiac patients in Khartoum State, Sudan: a cross-sectional study [89]	MMAS-8	No	Translated from English into Arabic	Self-administered questionnaire	No	None
Magnitude and factors associated with treatment non-adherence among patients with depressive disorders at St. Amanuel Mental Specialized	MMAS-8	Yes	Translated into Amharic	Interview	No	None

For peer review only - http://bmjopen.bmj.com/site/about/guidelines.xhtml

Hospital, Addis Ababa, Ethiopia, 2019: A cross sectional study [31]						
Self-Reported Adverse Drug Reactions, Medication Adherence, and Clinical Outcomes among Major Depressive Disorder Patients in Ethiopia: A Prospective Hospital Based Study [42]	MMAS-8	No	Translated into Amharic	Interview	No	None
Effect of patients beliefs about medications on adherence to drugs in diabetic patients attending family medicine outpatient clinic in Ismailia, Egypt [76]	MMAS-8	Yes	Arabic translation	Questionnaires	No	None
Adherence to Treatment and Factors Affecting Adherence of Epileptic Patients at Yirgalem General Hospital, Southern Ethiopia[18]	MMAS-8	No	Translated into Amharic and Sidama	Interview	No	None
Antiepileptic drug adherence in children in southern Ethiopia: A cross-sectional study[20]	MMAS-8	No	Translated into Amharic and Sidama	Interview	No	None
Beliefs about medication, medication adherence and seizure control among adult epilepsy patients in Kimberley, South Africa[96]	MMAS-8	No	No	Interview	No	None
Treatment adherence and associated factors among glaucoma patients attending Ophthalmic units of referral hospitals in North West Ethiopia [45]	MMAS-8	No	Translated into Amharic	Interview	No	None
Medication adherence in HIV-positive pregnant women on antiretroviral therapy attending antenatal clinics in ado metropolis, south-west nigeria: A multicentre study[63]	MMAS-8	No	Not specified	Interview	No	Pill Count Method
Mood and anxiety disorders adversely influence medication adherence to antiretroviral therapy among people living with HIV/AIDS in Nigeria[68]	MMAS-8	No	Not specified	Interview	No	None
Socio-demographic factors associated with medication adherence among People Living with HIV in the Kumasi Metropolis, Ghana[85]	MMAS-8	No	Not specified	Structured questionnaire	No	None
Adherence to antihypertensive medication and its correlates among individuals with hypertension in a semi-urban community of Southern Nigeria[52]	MMAS-8	Yes	Igbo language	Interview	No	None
Adherence to Antihypertensive Treatment and Associated Factors in Central Ethiopia[16]	MMAS-8	No	Amharic or Afaan Oromo	Interview	No	None

For peer review only

Antihypertensive medication adherence and associated factors among adult hypertensive patients at Jimma University Specialized Hospital, southwest Ethiopia [22]	MMAS-8	Yes	Amharic or Afaan Oromo	Interview	No	None
Factors associated with poor adherence to medication among hypertensive patients in twelve low and middle income Sub-Saharan countries [107]	MMAS-8	No	Via verbal translation	Self-administered with physician assistance / Interviewer-supported	No	None
Family support and medication adherence among residents with hypertension in informal settlements of Nairobi, Kenya: a mixed-method study [81]	MMAS-8	No	Not specified	Interview	No	None
Impact of adverse drug events and treatment satisfaction on patient adherence with antihypertensive medication – a study in ambulatory patients [29]	MMAS-8	No	Amharic and Tigrigna	Interview	No	None
Medication Adherence and Patient Satisfaction among Hypertensive Patients Attending Outpatient Clinic in Lagos University Teaching Hospital, Nigeria [61]	MMAS-8	No	Not specified	Interview	No	None
Medication Adherence and Perceived Family Support Among Elderly Patients with Hypertension Attending a Specialty Clinic in Lagos, Nigeria [62]	MMAS-8	No	Not specified	Interview	No	None
Relationship between Cost of Care and Medication Adherence among Hypertensive Patients Attending a Tertiary Health Facility in Ekiti State, Nigeria[71]	MMAS-8	No	Not specified	Interview	No	None
Treatment satisfaction and medication adherence among hypertensive patients in a teaching hospital in Ekiti State, Nigeria[74]	MMAS-8	No	Not specified	Questionnaire	Yes	Pill counts or pharmacy refill records
Validity and Reliability of a Self-Reported Measure of Antihypertensive Medication Adherence in Uganda[105]	MMAS-8	Yes	Runyankore/Rukiga	Questionnaire	Yes	None
Depression and medication-adherence in patients with hypertension attending a tertiary health facility in South-West Nigeria [54]	MMAS-8	No	Yoruba language	Interview	Yes	None
Magnitude and associated factors of poor medication adherence among diabetic and	MMAS-8	No	Amharic	Interview	Yes	None

hypertensive patients visiting public health facilities in Ethiopia during the COVID-19 pandemic[30]						
Status of rheumatoid arthritis practice and treatment in Sudan[10]	MMAS-8	No	Not specified	Interview	No	None
Prevalence and predictors of sub-optimal medication adherence among patients with severe mental illnesses in a tertiary psychiatric facility in Maiduguri, North-eastern Nigeria [70]	MMAS-8	No	Not specified	Interview	No	None
Barriers to diabetes medication adherence in North West Ethiopia[108]	MMAS-8	No	Amharic	Interview	No	None
Drug therapy problems, medication adherence and treatment satisfaction among diabetic patients on follow-up care at Tikur Anbessa Specialized Hospital, Addis Ababa, Ethiopia Gebre Teklemariam D[28]	MMAS-8	No	Not specified	Interview	No	None
Medication adherence and direct treatment cost among diabetes patients attending a tertiary healthcare facility in Ogbomosho, Nigeria [60]	MMAS-8	No	Not specified	Interview	No	None
Medication adherence and its associated factors among diabetic patients at Zewditu Memorial Hospital, Addis Ababa, Ethiopia[34]	MMAS-8	No	Not specified	Interview	No	None
Medication non-adherence and associated factors among diabetes patients in Felege Hiwot Referral Hospital, Bahir Dar city administration, Northwest Ethiopia[37]	MMAS-8	No	Amharic	Interview	No	None
Association of treatment satisfaction and physician trust with glycemic control among primary care patients with type 2 diabetes in Egypt [78]	MMAS-8	No	Arabic	Interview	No	None
Factors associated with medication non-adherence in patients with type 2 diabetes mellitus: The first Algerian survey[97]	MMAS-8	Yes	Arabic/French	Questionnaire	No	None
Illness perceptions of Libyans with T2DM and their influence on medication adherence: a study in a diabetes center in Tripoli	MMAS-8	No	Arabic	Self-administered / Self-reported questionnaire	No	None

llness perception, medication adherence and glycemic control among primary health-care patients with type 2 diabetes mellitus at Port Said City, Egypt [100]	MMAS-8	No	Arabic	Self-administered	No	None
Low medication adherence and its associated factors among patients with type 2 diabetes mellitus attending Amana Hospital in Dar es Salaam, Tanzania: a cross-sectional study [101]	MMAS-8	No	Not specified	Self-administered	No	None
Medication adherence and factors associated with poor adherence among type 2 diabetes mellitus patients on follow-up at Kenyatta National Hospital, Kenya[83]	MMAS-8	No	kiswahili	Interview	No	None
Medication Adherence in Type 2 Diabetes Patients in Nigeria[64]	MMAS-8	No	Not specified	Interview	No	None
The impact of pharmacist-led medication therapy management on medication adherence in patients with type 2 diabetes mellitus: a randomized controlled study [43]	MMAS-8	No	Not specified	Interview	No	None
The Influence of National Health Insurance on Medication Adherence Among Outpatient Type 2 Diabetics in Southwest Nigeria [72]	MMAS-8	No	Not specified	Interview	No	None
Validation and psychometric properties of the 8-item Morisky Medication Adherence Scale (MMAS-8) in Type 2 diabetes patients in sub-Saharan Africa [104]	MMAS-8	Yes	Translated from English to French and Ewe following international guidelines (forward translation, review, reverse translation, and pilot testing)	Interview	No	MMAS-4
Medication Adherence and Its Determinants Among Adults With Heart Failure: A Study in Selected Hospitals of North Shewa Zone, Oromia, Ethiopia (2024)-A Multicenter Institutional-Based Cross-Sectional Study [46]	MMAS-8	No	English to the local language (Amharic)	Interview	No	None
Non-adherence to antidiabetic medications and associated factors among adult type 2 diabetes mellitus patients in Northeast Ethiopia: institutional based cross-sectional study[47]	MMAS-8	No	English to the local language (Amharic)	Questionnaire, administered	No	None

Machine learning applications to classify and monitor medication adherence in patients with type 2 diabetes in Ethiopia[48]	MMAS-8	No	English to the local language (Amharic)	Interview	No	None
Cultural adaptation and psychometric validation of the Amharic adherence barriers questionnaire for HIV patients on antiretroviral therapy in Ethiopia[50]	MMAS-8	No	English to the local language (Amharic)	Interview	No	None
Adherence to hypertension management at a rural hospital in Limpopo: A cross-sectional study[94]	MMAS-8	No	English to the local language (Amharic)	Interview	No	None
Treatment adherence and blood pressure outcome among hypertensive out-patients in two tertiary hospitals in Sokoto, Northwestern Nigeria [73]	MMAS-9	No	Not specified	Interview	Yes	Modified Morisky Adherence Predictor Scale (MMAPS), Brief Illness Perception Questionnaire (BIPQ) and Beliefs about Medicine Questionnaire (BMQ)
Factors contributing to antiretroviral drug adherence among adults living with HIV or AIDS in a Kenyan rural community[80]	PMAQ	No	Kamba translation	Interview	Yes	Self-reports / Patient recall and Pill counts
Incomplete adherence among treatment-experienced adults on antiretroviral therapy in Tanzania, Uganda and Zambia[111]	Provider report and pharmacy refill	No	Not specified	Interview	Yes	Pharmacy Medication Possession Ratio (MPR), Healthcare Provider Reports and Biological Marker
Medication compliance behavior in psychiatric out-patients with psychoactive substance use comorbidity in a Nigerian tertiary hospital[67]	Self-Administered Medication Adherence Scale	No	Not specified	Interview	No	None
Factors Influencing Adherence to Antiretroviral Therapy Among Adolescents at Busia County Referral Hospital [88]	Self-report questionnaire	Not applicable	English and administered in a bilingual	Interview	Yes	Pharmacy refill registries and clinic appointment

For peer review only

			format/adapted to Kiswahili			attendance records
Food Insecurity Is Associated with Increased Risk of NonAdherence to Antiretroviral Therapy among HIV-Infected Adults in the Democratic Republic of Congo: A Cross-Sectional Study[99]	Self-reported Adherence	No	Translated into French or Lingala	Interview	No	None
Breast Nurse Intervention to Improve Adherence to Endocrine Therapy Among Breast Cancer Patients in South Ethiopia [27]	SMAQ	No	Translated from English to Amharic	Interview	No	Monitoring of drug refill registration forms (Medication Possession Ratio - MPR
Prevalence and determinants of adherence to antiretroviral treatment among HIV patients on first-line regimen: a cross-sectional study in Dakar, Senegal [103]	SMAQ	No	Not specified	Interview	No	Self-reporting tool and Visual Analogue Scale (VAS)
The role of health facility and individual level characteristics on medication adherence among PLHIV on second-line antiretroviral therapy in Northeast Ethiopia: use of multi-level model[44]	SMAQ	No	Not specified	Interview	No	None
Medication non-adherence and its associated factors among kidney transplant patients in a large teaching hospital in Ethiopia[38]	SMAQ	No	Amharic	Interview	No	None
Using a Self-Administered Electronic Adherence Questionnaire to Identify Poor Adherence Amongst Adolescents and Young Adults on First-Line Antiretroviral Therapy in Johannesburg, South Africa[95]	SMAQ and South African National Department of Health (NDoH) adherence questionnaire	No	Not specified	Self-administered electronic questionnaire	Yes for NDoH and no for SMAQ	Viral Load Monitoring (Standard of Care) and Therapeutic Drug Monitoring (TDM)
General self-efficacy and hypertension treatment adherence in Algerian private clinical settings [98]	Treatment Adherence Scale	No	Not specified	Self-administered questionnaires	Yes	None

2

3

10

For peer review only - http://bmjopen.bmj.com/site/about/guidelines.xhtml
