# Supplementary material for: Mapping medication adherence tools in Africa: a scoping review
Source: BMJ Open. 2026 Sep 16;16(9):e112898. doi: 10.1136/bmjopen-2025-112898 (PMC13584023; doi:10.1136/bmjopen-2025-112898)
Supplement: Supplementary file 6 [file bmjopen-2025-112898.reviewer_comments.pdf]

PEER REVIEW HISTORY

BMJ Open publishes all reviews undertaken for accepted manuscripts. Reviewers are asked to complete a checklist review form and are provided with free text boxes to elaborate on their assessment. These free text comments are reproduced below.

ARTICLE DETAILS

Title (Provisional)

Mapping Medication Adherence Tools in Africa: A Scoping Review

Authors

Muchanga, Izaidino Jaime; Midão, Luís; Mazuze, Avelino; Melo, Daniela; Alcântara, Leovaldo; Figueiredo, Teodora; Costa, Elisio; Sousa-Pinto, Bernardo

---

VERSION 1 - REVIEW

**Reviewer**	**1**
**Name**	**Apte, Aditi**
**Affiliation**	**KEM Hospital Pune Research Centre**
**Date**	**18-Feb-2026**
**COI**	**None**

Introduction

1. The review is focused on objectives for mapping the medication adhrence tools for African settings. However, the authors have not provided appropriate background for need for this review and the gap in literature. The introduction section focuses more on the low adhrence rates

2. The introduction should be made more concise reducing text on low medication adhrence.

Objectives.

The objectives should be stated clearly (depending on the RQ)- because the results talk about 1. type of medication adherence tools (RQ1) , 2. Adherence rates (this was not mentioned as RQ2) , 3. Challenges to assess medication adherence tools (RQ3)

Results

The data extraction table is oversimplified and does not include crucial outcome details. It should be elaborate to include details about validation of medication adhrence tool for the given study, timepoint for administration, whether it was self administered or interviewer administered, appropriateness of the tool in terms of the disease condition.

Also, whether any other gold standard method for medication adherence was included in the study e.g. pill count. The data extraction can be divided as study characteristics table and outcome assessment table.

Page 11: Line 27- They have written systematic review instead of scoping review

There error in the total number of articles reviewed: Total number of records identified in the database should be 6514 and not 6541. Please check.

The risk of bias assessment has not been done.

The authors have captured data on rates of medication adherence, however, the relevance of these data for the given review is not clear. Do the numbers show hetergenecity which can be due lack of validation?

Discussion

1. The discussion is not focused on the results rather authors discuss about the lack of validation of the tools which is not included in the results.

2. Details of the scales used the studies reviewed in terms of their appropriateness needs to be discussed.

3. More seggregated results for different disease conditions should be discussed.

4. Limitations of the study need to be discussed.

**Reviewer**	**2**
**Name**	**Moges, Tilaye Arega**
**Affiliation**	**Debre Tabor University, Pharmacy**
**Date**	**21-Mar-2026**
**COI**	**None**

This scoping review effectively targets a significant gap in measuring treatment adherence in Africa and adheres to PRISMA-ScR guidelines, enhancing its credibility. However, it needs revisions: it should acknowledge potential biases from restricting studies to English and consider including regional sources like African Journals Online. Additionally, despite the lack of formal quality appraisal for scoping reviews, reliability conclusions require cautious wording. The overall conclusions would benefit from more precise language regarding the need for new tools versus validating existing ones. Acceptance is recommended after minor to moderate revisions.

VERSION 1 - AUTHOR RESPONSE

REVIEWER: 1

INTRODUCTION

Comment 1:

The authors have not provided appropriate background for the need for this review and the gap in literature.

Answer: The Introduction has been revised to better explain why it is important to comprehensively map existing tools for assessing medication adherence in the African context. Some examples of added information include:

Medication adherence generally refers to the extent to which patients take medications as prescribed and continue treatment according to healthcare recommendations. Adherence to prescribed treatment regimens is essential for achieving successful therapeutic outcomes, especially in chronic disease management, where it enhances chronic disease prognosis and reduces long-term healthcare expenditures. Non-adherence constitutes a major public health challenge associated with poorer clinical outcomes, reduced quality of life, increased morbidity and mortality, and substantial healthcare costs. Current evidence indicates that approximately 50% of patients receiving long-term treatment for chronic conditions do not adhere adequately to prescribed therapies, with lower adherence rates being frequently observed in low and middle-income countries, including Sub-Saharan Africa, where both communicable and non-communicable disease burdens remain high.

Appropriate tools are essential for measuring medication adherence accurately. Various methods have been described, including direct methods, such as measurement of drugs or metabolites in biological samples, and indirect methods, such as pill counts, pharmacy refill records, electronic monitoring systems, and self-reported questionnaires. The selection of an appropriate adherence assessment approach should consider the clinical context and characteristics of the target population, particularly in African settings where socioeconomic and cultural factors may significantly influence adherence behaviors and reporting accuracy. However, despite the importance of adherence assessment in African countries, there is limited availability of validated and culturally adapted scales. The most frequent adherence scales, such as the Morisky-Green scale (MGS), the Morisky Medication Adherence Scale (MMAS-4 and MMAS-8 (MMAS) the Medication Adherence

Rating Scale (MARS), and the Medication Compliance Questionnaire (MCQ), were developed and validated outside Africa, raising concerns about their direct applicability in African populations due to cultural, linguistic, and socioeconomic differences..

Although previous studies have reported medication adherence levels across different African countries and disease conditions, there remains a substantial gap in the systematic synthesis of the performance, validity, and diversity of instruments used to assess medication adherence across the continent have not been comprehensive evaluated. In fact, to date, no effort has been made to map the available adherence assessment tools while simultaneously identifying operational and methodological challenges associated with their use in African populations. Such processes are essential steps toward identifying (i) the best tools to measure adherence in the African context, and (ii) gaps that need to be overcome by the development of standardized and contextually appropriate adherence assessment protocols (Pages 2–3, Lines 60-96).

Comment 2:

The introduction should be made more concise.

Answer: The Introduction was substantially shortened and refocused on the rationale, evidence gap, and objectives of the review. Excessive discussion of medication adherence rates was removed (Pages 2–3, Lines 62-100).

Comment:

The objectives should be stated clearly.

Answer: The objectives were revised to align with the review questions and results. In particular, we now state that: Therefore, this scoping review we aimed to map and characterize medication adherence assessment tools used in African populations with chronic diseases, including their validation status, cultural adaptation processes, administration characteristics, and reported adherence outcomes (Page 3, Lines 97-100).

Comment:

Results

The data extraction table is oversimplified.

Answer: We completely revised the data extraction framework and divided the extracted information into two complementary tables. Table 1 now presents study characteristics, including country, disease condition, population, study design, adherence tool, and

adherence rates (Pages 15-23). Table 2 presents methodological characteristics of adherence assessment tools, including local validation status, translation and cultural adaptation procedures, administration mode, disease specificity, and use of additional adherence assessment methods such as pill counts, pharmacy refill records, biological markers, and electronic monitoring (Pages 23-41).

Comment:

Page 11 line 27 – systematic review instead of scoping review.

Answer: This typographical error has been corrected.

Comment:

Total number of records should be 6514 and not 6541.

Answer: Initially, the PRISMA flow diagram contained an error, indicating that the total number of records identified was 6.541 instead of the correct 6.514. However, following the editor's recommendation, the literature search was updated to include studies published up to 25 June 2026. As a result, the total number of records identified increased to 6.622. Accordingly, the database counts, PRISMA flow diagram, and all corresponding text throughout the manuscript were revised to reflect the updated search results, ensuring consistency across the manuscript. (PRISMA Flow Diagram, Page 8 and Results section, Page 7).

Comment:

The risk of bias assessment has not been done.

Answer: In accordance with PRISMA-ScR guidance, formal methodological quality appraisal and risk-of-bias assessment were not undertaken because the objective of this review was to map the available evidence rather than evaluate study effectiveness. This has now been explicitly stated: *Consistent with PRISMA-ScR recommendations and the exploratory nature of scoping reviews, formal methodological quality appraisal or risk of bias assessment was not conducted. In fact, the objective of this study was to map evidence rather than determine intervention effectiveness. (Page 6, Lines 172-175).*

DISCUSSION

Comment 1:

The discussion is not focused on the results.

Answer: The Discussion was extensively revised to focus more directly on the principal findings of the review, including the predominance of MMAS-8, methodological heterogeneity, geographic distribution of studies, and variation in

adherence rates. Some examples include: *This scoping review mapped medication adherence assessment tools used across African populations with chronic diseases and identified substantial methodological heterogeneity in adherence measurement. A total of 98 studies conducted in 27 African countries were identified, with the MMAS-8 being the most frequently used scales, followed by MARS and other scales such as SMAQ, MCQ, PMAQ and CQR, used in specific contexts.*

Adherence rates showed high variability among the 88 studies included in this scoping review, ranging from 4% to 96.8%, depending on the country, the health condition, and the instrument used. This wide discrepancy may reflect both methodological challenges such as the use of non-standardised instruments and contextual factors, such as the type of disease, the age group of participants, and the characteristics of the health system in place. Chronic diseases such as hypertension and diabetes generally showed lower adherence rates, which is consistent with african studies. On the other hand, conditions such as infection with the HIV demonstrated relatively higher adherence rates in some contexts, possibly due to greater investment in adherence programs in countries like Kenya and South Africa.

The widespread use of the MMAS-8 adherence scale was consistent with findings from studies conducted in various African contexts, as well as international studies, which highlight its simplicity, practicality, reliability, and applicability across multiple clinical conditions. This scale remains one of the most widely used self-report adherence measures due to its simplicity, low implementation cost, and ease of administration (118). However, the large number of instruments identified, including MMAS-4, MARS, SMAQ, GMAS, BMQ, MAQ, PMAQ, CQR, and several indirect methods, reflects the absence of a standardized approach for adherence measurement in African settings. This methodological diversity may partly explain the substantial variability observed in adherence estimates across studies (Pages 42–45).

Comment 2:

Appropriateness of scales should be discussed.

Answer: A new paragraph in the discussion was added addressing the appropriateness of generic versus disease-specific adherence instruments and their suitability for different clinical contexts: *Beyond validation status, the findings raise important questions regarding the appropriateness of adherence instruments across different disease conditions. Most studies relied on generic self-report tools regardless of the clinical condition under investigation. While instruments such as MMAS and MARS are practical and easy to administer, they may not adequately capture disease-specific adherence challenges* (Page 43-44, Lines 58-69) .

Comment 3: More segregated results for different disease conditions should be discussed.

Answer: We have now added a paragraph to the Discussion section, discussing differences observed across different diseases: *Medication-taking behaviour among individuals living with HIV differs considerably from that observed in patients with diabetes, hypertension, epilepsy, or severe mental illness. Disease-specific tools such as SMAQ for HIV or CQR for rheumatologic diseases may provide more clinically relevant information because they address treatment characteristics unique to these conditions. (Pages 43-44, Lines 62-67).*

Comment 4:

Limitations of the study need to be discussed.

Answer: A paragraph on the limitations of the study has been added*: This study has some limitations. Although a systematic search strategy was conducted across various databases for this scoping review, it is possible that some relevant studies were not identified, the studies published in English and French were included, potentially excluding relevant evidence published in other languages. As recommended for scoping reviews, no formal methodological quality appraisal or risk-of-bias assessment was conducted. The heterogeneity among the studies in terms of clinical conditions and populations assessed, application contexts, and languages used may limit comparisons between scales and the generalization of results to different African settings. The geographical distribution of studies was uneven, with a strong concentration in Ethiopia and Nigeria and limited evidence from several African countries, which may affect the generalizability of findings across the continent (Page 45, Lines 97-108).*

REVIEWER: 2

Comment:

The review should acknowledge potential biases from restricting studies to English and consider regional databases such as African Journals Online.

Answer: Thank you for this important comment. We acknowledge that restricting inclusion to English and French publications may have excluded relevant studies published in other languages. We also recognize that several regional and online African databases exist, including African Journals Online (AJOL), which may contain additional relevant studies. However, many African journals with higher scientific visibility and impact are also indexed in PubMed, which was included among the databases searched in this review. Therefore, we believe that the main body of high-impact and widely accessible African research was captured. Nevertheless, the possibility of missing studies indexed exclusively in regional databases remains a limitation, and this has been added to the manuscript (Page 45).

Comment:

Reliability conclusions require cautious wording.

Answer: The Discussion and Conclusion were revised to avoid overinterpretation of reliability findings. Conclusions now emphasise the limited evidence regarding validation and the need for further context-specific validation studies (Discussion, Pages 42–45 and Conclusion, Page 45).

Comment:

The conclusion should focus on validation of existing tools rather than development of new tools.

Answer: The Conclusion was revised accordingly. Rather than just advocating for the development of new instruments, we now emphasise the validation, cultural adaptation, and standardisation of existing medication adherence assessment tools within African contexts (Conclusion, Page 45).
